# Discovery of WRN inhibitor HRO761 with synthetic lethality in MSI cancers

**DOI:** 10.1038/s41586-024-07350-y

**Published:** 2024-04-24

**Authors:** Stephane Ferretti, Jacques Hamon, Ruben de Kanter, Clemens Scheufler, Rita Andraos-Rey, Stephanie Barbe, Elisabeth Bechter, Jutta Blank, Vincent Bordas, Ernesta Dammassa, Andrea Decker, Noemi Di Nanni, Marion Dourdoigne, Elena Gavioli, Marc Hattenberger, Alisa Heuser, Christelle Hemmerlin, Jürgen Hinrichs, Grainne Kerr, Laurent Laborde, Isabel Jaco, Eloísa Jiménez Núñez, Hans-Joerg Martus, Cornelia Quadt, Markus Reschke, Vincent Romanet, Fanny Schaeffer, Joseph Schoepfer, Maxime Schrapp, Ross Strang, Hans Voshol, Markus Wartmann, Sarah Welly, Frédéric Zécri, Francesco Hofmann, Henrik Möbitz, Marta Cortés-Cros

**Affiliations:** 1 Novartis BioMedical Research, Basel, Switzerland; 2 Novartis Pharma AG, Basel, Switzerland; 3 Novartis BioMedical Research, Cambridge, MA USA; 4 Present Address: Pierre Fabre Laboratories, Toulouse, France

**Keywords:** Targeted therapies, Drug development

## Abstract

The Werner syndrome RecQ helicase WRN was identified as a synthetic lethal target in cancer cells with microsatellite instability (MSI) by several genetic screens^1–6^. Despite advances in treatment with immune checkpoint inhibitors^7–10^, there is an unmet need in the treatment of MSI cancers^11–14^. Here we report the structural, biochemical, cellular and pharmacological characterization of the clinical-stage WRN helicase inhibitor HRO761, which was identified through an innovative hit-finding and lead-optimization strategy. HRO761 is a potent, selective, allosteric WRN inhibitor that binds at the interface of the D1 and D2 helicase domains, locking WRN in an inactive conformation. Pharmacological inhibition by HRO761 recapitulated the phenotype observed by WRN genetic suppression, leading to DNA damage and inhibition of tumour cell growth selectively in MSI cells in a p53-independent manner. Moreover, HRO761 led to WRN degradation in MSI cells but not in microsatellite-stable cells. Oral treatment with HRO761 resulted in dose-dependent in vivo DNA damage induction and tumour growth inhibition in MSI cell- and patient-derived xenograft models. These findings represent preclinical pharmacological validation of WRN as a therapeutic target in MSI cancers. A clinical trial with HRO761 (NCT05838768) is ongoing to assess the safety, tolerability and preliminary anti-tumour activity in patients with MSI colorectal cancer and other MSI solid tumours.

## Abstract (web summary)

HRO761 is a potent, selective, allosteric WRN inhibitor that binds at the interface of the D1 and D2 helicase domains, locking WRN in an inactive conformation.

## Main

**Extended Data Fig. 1:**
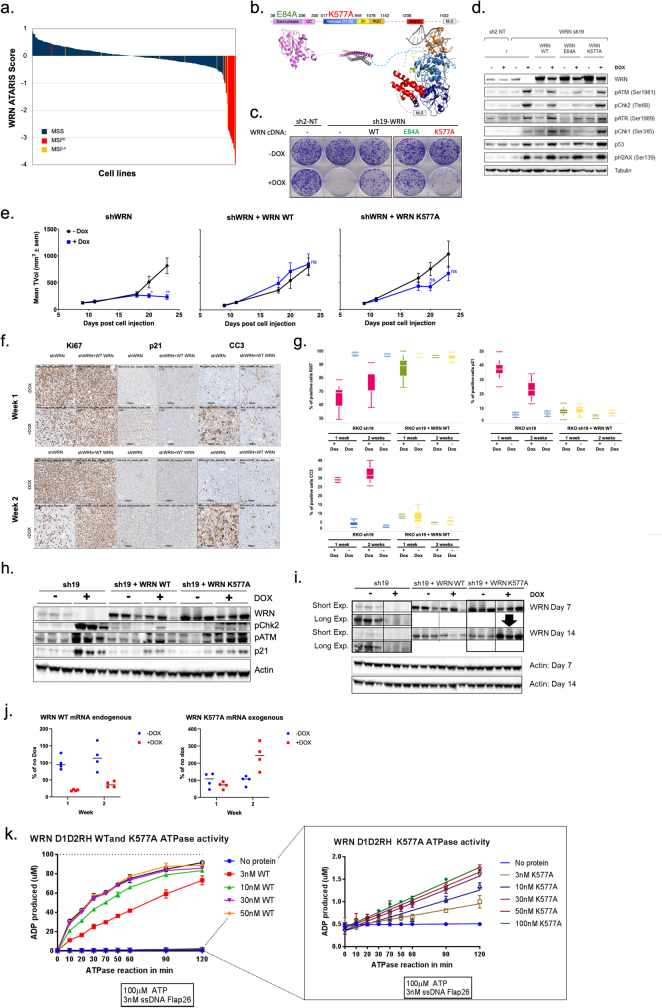
Genetic validation of WRN helicase dependence. **a**. WRN ATARIS dependency scores from DRIVE coloured by MSI (high-red and Low-orange) and MSS (blue) status. **b**. Schematic representation of WRN helicase domains. Clonogenic assay (**c**) or immunoblot (**d**) of RKO cells doxycycline induction of a non-targeting negative control (sh2-NT), and one shRNA against WRN (sh19) without (-) or with a wild type (WT), exonuclease mutant (E84A) or helicase mutant (K577A) WRN cDNA. Clonogenic assay shown 15 days and immunoblot 4 days after dox induction. **e**. RKO xenograft growth ± doxycycline induction of WRN sh19 with WT or helicase dead (K577A) WRN cDNA. Mice were randomized (n = 6). Differences between the means of TVol were assessed on the endpoint ∆TVol using a two-tailed t-test (^*^ P < 0.05, ^**^ P < 0.01 and ns is non-significant). **f**. Ki67, p21 and cleaved caspase 3 (CC3) expression were evaluated on RKO xenograft sections by immunohistochemistry 7 and 14 days after treatment ± doxycycline and ± WT WRN cDNA. **g**. Quantification of Ki67, p21 and CC3 positive cells from FFPE sections. The white centre line denotes the median value, while the filled boxes contain the first quartile (25th) and third quartile (75th) percentiles of the dataset. The coloured whiskers mark the 5th and 95th percentiles. **h**. Immunoblot for WRN, pCHK2, ATM, p21 and actin of RKO tumour ± doxycycline and ± WT WRN cDNA. **i**. Immunoblot for WRN from samples from panel h treated for 7 and 14 days at different exposures. **j**. RTqPCR for WRN WT (endogenous) and WRN K577A mutant from tumours treated with doxycycline for 1 and 2 weeks. Data are mean of expression compared to no Dox treatment ± SD, n = 4 tumours. **k**. Quantification of ADP production over time in an ATPase assay at indicated conditions for both WRN WT and WRN K577A D1D2RH helicase domain protein. Data represent mean ± SD, n = 3. Source Data

Loss of DNA mismatch repair (MMR) by either germline or somatic mutations and epigenetic alterations occurs in 10–30% of colorectal, endometrial, ovarian, gastric and other cancer types^15,16^. Cancers that are MMR deficient have a high mutational burden and frequent insertion and/or deletion events in repetitive DNA tracts—a phenotype known as MSI^17^. Several large-scale functional genomics screens, including project DRIVE with 390 cell lines from the Cancer Cell Line Encyclopedia, have identified WRN as a synthetic lethal target in MSI cells^1–6^ (Extended Data Fig. 1a). Genetic depletion of WRN was shown to lead to DNA damage, anti-proliferative effects, mitotic defects with cell cycle arrest, chromosome shattering and apoptosis in MSI cancer models, but not in cancer cells with an intact MMR pathway. Moreover, WRN depletion in MSI cells reduced xenograft growth and tumour formation in mice^6^. Recent studies offered insights into the mechanism of WRN dependence. WRN dependence in MSI cells was linked to WRN helicase-mediated resolution of secondary DNA structures resulting from large-scale expansions of dinucleotide TA repeats that otherwise result in chromosome breakage^18^. Although MSI cancers have a high response rate to immune checkpoint inhibitors^7–10^, a substantial fraction of patients with MSI colon cancers does not benefit from current treatment regimens^11–14^.

WRN is a multifunctional enzyme with both helicase and exonuclease activities and has roles in various cellular processes that are crucial for the maintenance of genome stability, including DNA replication, transcription, DNA repair and telomere maintenance^19–22^. Dissection of the helicase and exonuclease enzymatic activities of WRN using loss-of-function mutations showed that WRN dependency in vitro and in vivo in MSI cells is linked only to its helicase function (Extended Data Fig. 1b–e). Reintroduction of *WRN* WT cDNA after short hairpin RNA (shRNA) knockdown of *WRN* rescued the DNA damage and cell proliferation phenotype, whereas *WRN*^*K577A*^ cDNA did not. Notably, tumours expressing the helicase point mutant WRN(K577A) upregulated its expression to compensate for the loss of endogenous WRN as an escape mechanism and revealed that the K577A helicase mutant retains a very low level of activity^6,19^ (Extended Data Fig. 1f–k and Supplementary Information).

**Fig. 1:**
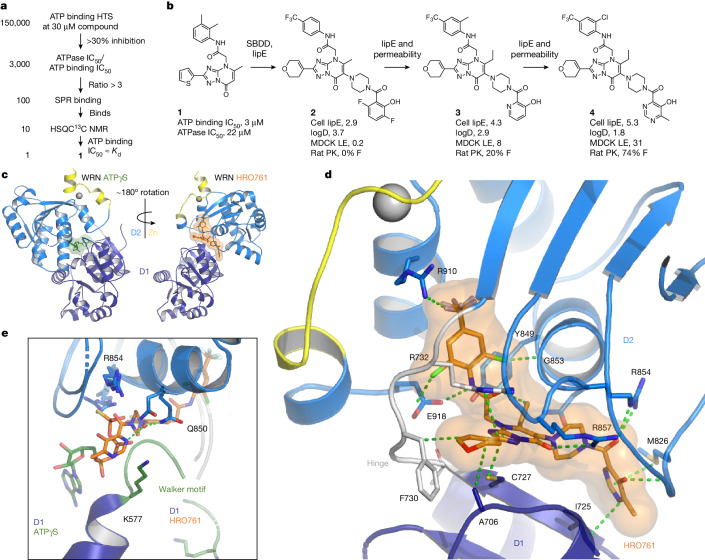
Identification and structural basis of HRO761, an allosteric WRN inhibitor. **a**, Screening funnel with hit count on the left and progression criteria on the right leading to the identification of hit **1**. HTS, high-throughput screening; *K*_d_, dissociation constant; NMR, nuclear magnetic resonance; SPR, surface plasmon resonance. **b**, The structure of hit **1** and medicinal chemistry optimization to clinical candidate **4**, HRO761, with key profiling data of compounds **2**–**4** (cell lipE calculated from SW48 proliferation GI_50_ and the distribution coefficient between 1-octanol and water at pH 7.4 (logD), apparent permeability in low-efflux Madin–Darby canine kidney cells (MDCK LE *P*_app_, 10^−6^ cm s^−1^), oral bioavailability (F) and structure based drug design (SBDD)). **c**, HRO761 is an allosteric inhibitor of the WRN helicase binding at the D1–D2 interface in a novel conformation involving a 180° rotation of the D1 and D2 domains relative to ATPγS-bound WRN (ligands are shown as sticks with transparent surface). **d**, Owing to the overlap with the D2 ATP half-site, the HRO761-binding site is unusually polar and rich in arginine residues. HRO761 makes extensive polar interactions and engages key residues of the flexible hinge (Thr728-Gly-Phe-Asp-Arg). **e**, Overlay of the D2 domains of ATPγS- and HRO761-bound WRN showing that HRO761 displaces the Walker motif (green) and its catalytic residue Lys577 through mimicry of the ATP γ-phosphate, including coordination of the hydrolytic water by Gln850.

**Extended Data Fig. 2:**
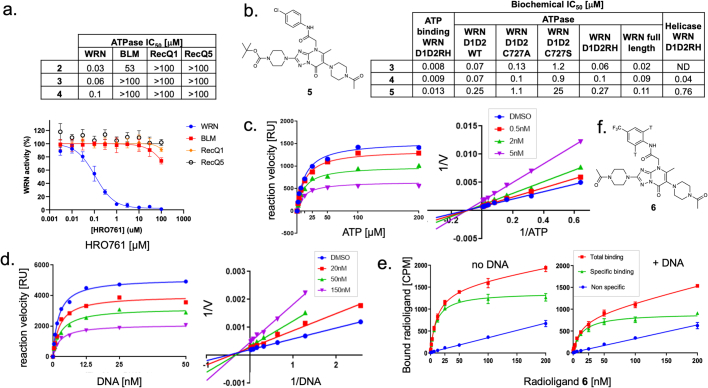
HRO761 and related WRN inhibitors are selective WRN inhibitors mixed competitive with ATP and uncompetitive with DNA. **a**. Biochemical ATPase IC_50_ activity and concentration response curves of the WRN inhibitors HRO761 (**4**), and analogues **2** and **3** on WRN as well as RecQ helicases BLM, RecQ1 and RecQ5. Data represent mean of quadruplicates expressed as %inhibition ± SD. **b**. Comparison of biochemical ATP binding, ATPase and helicase IC_50_ across different WRN constructs and mutants (reported IC_50_ values in **a** and **b** are the geometrical means of at least 2 independent experiments., structure of **5** shown on the left.). **c**. Michaelis-Menten plots of the WRN ATPase assay (0.5 nM WRN D1D2RH, 3 nM ssDNA FLAP26, 50 mM NaCl) at varying concentrations of **4** (for legend, see inset) revealed a non-competitive or mixed mode of action (Lineweaver-Burk plot on the right), interpreted as mixed ATP-competition due to competition in ATP binding assay (b.) **d**. K_DNA_ determination in the ATPase assay (0.5 nM WRN D1D2RH, 100 µM ATP, 150 mM NaCl, ssDNA: FLAP26) at varying concentrations of **5** (for legend, see inset) revealed a non-competitive or mixed mode of action (double reciprocal plot on the right), interpreted as a non-competitive mode of action due lack of DNA competition in a radioactive binding assay (e.). For **c**, **d**, Data represent initial Kobs determined by following the ATPase reaction for 20 min. **e**. Saturation binding experiments of radiolabeled **6** (structure shown in **f**.) performed with 10 nM WRN (D1D2RH) in absence (left) or in presence of 50 nM dsDNA (right) using a Scintillation Proximity Assay resulted in a similar K_d_ (10/14 nM). Data points represent the mean of triplicate ±SD (each experiment was repeated at least twice).

Our initial screens with WRN helicase assays (DNA unwinding, ATPase) identified only covalent hits, as reported for other WRN hit-finding campaigns^23–25^. We characterized them as allosteric, ATP-competitive inhibitors targeting Cys727. With the aim of identifying weaker, non-covalent hits that were not detected in the enzymatic screens, we developed an ATP-binding assay. Validated WRN inhibitors (for example, covalent hits, ATPγS) were around sevenfold more potent in this assay compared with in the ATPase assay at ATP *K*_M_, whereas false positives (for example, compounds losing potency after repurification) were equipotent in all of the assays. This observation was key to overcoming the main challenge—the abundance of false positives. To identify real hits among the primary hits from a 150,000-compound ATP-binding screen, we determined half-maximal inhibitory concentration (IC_50_) values in both formats and used a shift above threefold as an indicator of real binding to cherry pick hits for biophysical validation. This screening strategy resulted in a single, non-covalent hit, compound **1** (Fig. 1a). Improving the potency by structure-based design, guided by lipophilic efficiency (lipE, potency corrected for the distribution coefficient between water and *n*-octanol (logP)), could only be achieved at the expense of high molecular mass and poor physicochemical properties, as illustrated by cellular tool compound **2** (Fig. 1b). Our medicinal chemistry strategy was to improve permeability and lipE simultaneously, two properties that are typically anticorrelated, by using physics-based property prediction (logP, 3D polar surface area) to systematically search for outlier transformations^26^. Two examples of transformations that lower logP while improving permeability are the introduction of the ortho-methyl aniline in compound **3** and the hydroxy pyrimidine in compound **4**, HRO761. Despite its molecular mass of 702 Da, HRO761 has favourable physicochemical properties and pharmacokinetics (PK), as well as a clean off-target profile (Extended Data Table 1). A co-crystal structure of HRO761 in a complex with the core helicase of WRN (consisting of the two RecA-like helicase lobes D1 and D2) revealed that HRO761 binds to a non-conserved allosteric site at the D1–D2 interface (Fig. 1c,d), rationalizing the high selectivity over related RecQ helicases (Extended Data Fig. 2a). While HRO761 and ATP analogues both bind at the D1–D2 interface with overlap in the D2 residues, the relative orientation is rotated by approximately 180° (Fig. 1c) due to a conformational change of residues 728–732 (Thr-Gly-Phe-Asp-Arg), a flexible hinge between the D1 and D2 domains. Most hinge residues are engaged by HRO761 and almost every heavy atom of HRO761 is engaged in polar interactions with WRN (Fig. 1d). The involvement of ATP-binding residues makes the pocket unusually polar and rich in arginine residues. The structure also rationalizes the mode of action with regard to ATP and DNA (Extended Data Fig. 2c–e). The domain rotation induced by HRO761 splits the ATP-binding site in two and displaces the Walker motif, resulting in mixed ATP competition through allosteric binding of HRO761. An overlay of the D2 domains of ATPγS- and HRO761-bound WRN shows that the hydroxy pyrimidine moiety of HRO761 mimics the γ-phosphate of ATP and recapitulates coordination of the hydrolytic water by the catalytic residue Gln850 (Fig. 1e). Both DNA-binding sites on the D1 and D2 domains remain accessible, explaining the lack of DNA competition. As none of the published SF2 helicases in the Protein Data Bank (PDB) remotely resembles the inactive conformation induced by HRO761, we hypothesize that it represents a high-energy conformation stabilized by the complementarity of HRO761 with the D1–D2 interface.

**Fig. 2:**
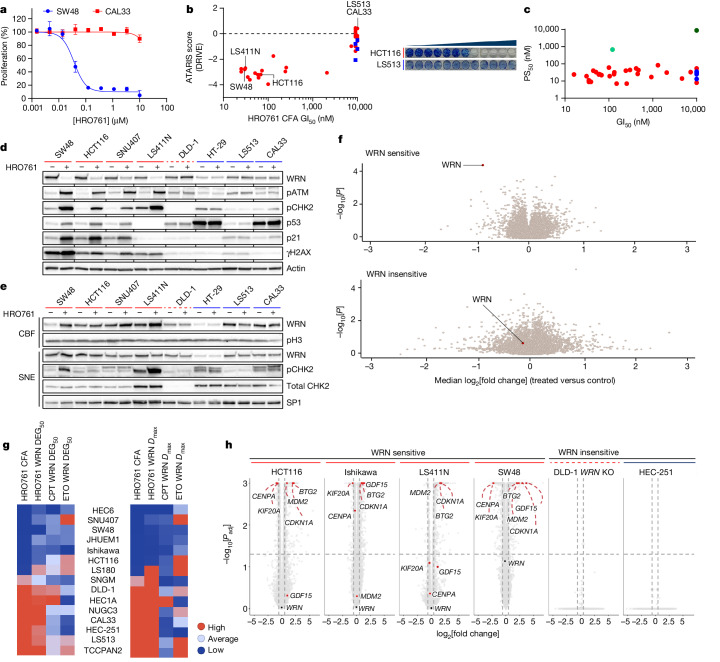
WRN inhibitors recapitulate synthetic lethality in MSI but not MSS cells and lead to DNA trapping. **a**, Representative survival curve of SW48 (MSI) and CAL33 (MSS) cells exposed to HRO761 for 4 days (CellTiter-Glo). Data are the mean ± s.d. percentage change compared with the DMSO control group. *n* = 3. **b**, The colony-formation assay (CFA) GI_50_ for HRO761 treatment in MSI (red, *n* = 20) and MSS (blue, *n* = 8) cells versus the ATARIS WRN dependency score (DRIVE). Right, representative CFA images of HTC116 and LS513 cells after 10 days. **c**, PS_50_ versus GI_50_ for HRO761 treatment in MSI (red) and MSS (blue) cells in the CellTiter-Glo assay. HCT116 and RKO cells with a C727A or C727S knock-in mutation at the *WRN* gene are shown in light and dark green, respectively. *n* = 28 (MSI) and *n* = 5 (MSS). **d**, Immunoblot analysis of WRN, phosphorylated ATM (pATM; Ser1981), pCHK2 (Thr68), p53, p21, γH2AX and actin after treatment with HRO761 for 24 h (WRN) or 8 h (other markers). **e**, Immunoblot analysis of the chromatin-bound fraction (CBF) and soluble nuclear extracts (SNE) of MSI and MSS cells treated with HRO761 at 10 µM for 1 h. **f**, Volcano plot showing *P* values (paired *t*-tests) plotted against median log_2_[fold change] of protein levels of three MSI cells (top) and one MSS cell (bottom) after treatment with compound **3** for 24 h (*n* = 2). **g**, The sensitivity to HRO761 was determined using a CFA, and WRN degradation was analysed after treatment with HRO761, camptothecin (CPT) or etoposide (ETO) in 15 cell lines. The compound concentration needed to achieve 50% WRN degradation (DEG_50_; left) and maximal degradation (*D*_max_; right) are shown. **h**, Differentially expressed genes in bulk RNA-seq data from cells treated with compound **2** (cases) compared with untreated cells (controls). Statistical significance was determined using two-sided Wald tests (in DEseq2), followed by Benjamini–Hochberg multiple-testing correction (*n* = 3). *P*_ad__j_, adjusted *P*.

**Extended Data Fig. 3:**
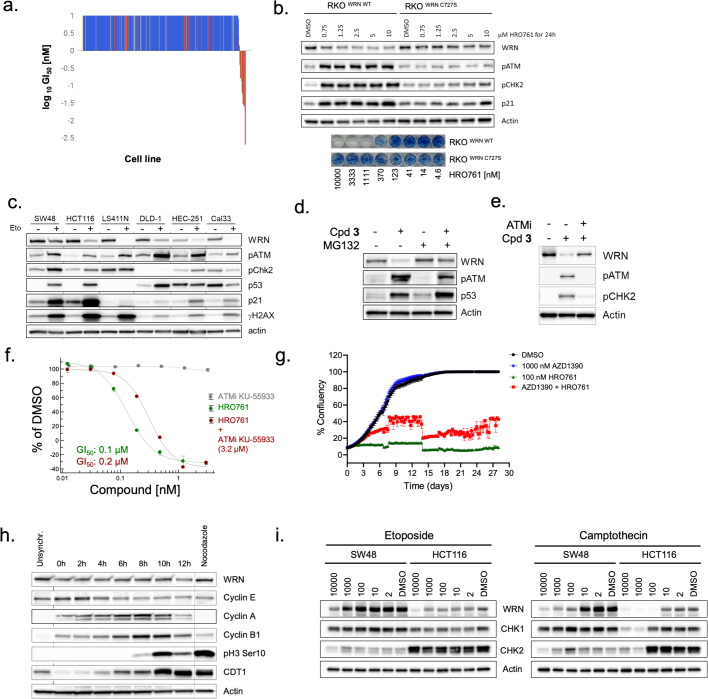
WRN degradation is proteasome and DNA damage mediated. **a**. Waterfall plot bar graph display of GI_50_ values of the 301 Horizon OncoSignature cell line panel. Cells were exposed to HRO761 for 120 h. MSI: red, MSS: blue. **b**. Immunoblot for WRN, pATM (S1981), pCHK2 (T68), p21 and actin of RKO parental and RKO WRN C727S knock-in cells after treatment of HRO761 for 24 h. Bottom. Representative image of a 15-day CFA of the same cells treated with HRO761. **c**. Immunoblot for WRN, pATM (S1981), pCHK2 (T68), p53, p21, γH2AX and actin after treatment with etoposide for 24 h. **d**. Immunoblot for WRN, pATM (S1981), p53 and actin from HCT116 cells treated with compound **3** at 10 µM for 24 h with or without proteasome inhibitor MG132 at 1 µM for 24 h. **e**. Immunoblot for WRN, pATM (S1981), pCHK2 (T68) and actin after treatment with co-treatment with 1 µM **3** and 1 µM KU-55933 (ATMi) for 24 h in HCT116 cells. **f**. Survival curves of SW48 cells treated with 96 h with the indicated concentrations of HRO761 (green) and ATMi KU-55933 (grey) alone or the combination of HRO761 dose response with 3.2 µM of KU-55933. Data represent mean ± SD, n = 3 **g**. Incucyte generated confluence plot from SW48 cells exposed to a combination of HRO761 and ATMi AZD1390 at the indicated concentrations. Data represent mean ± SD, n = 2. Graphs from **f** and **g** are from a representative experiment (n = 3). **h**. Immunoblot after double thymidine block and release of HCT116 cells harvested at the indicated timepoints after release as described in the methods section. Cells were treated with 330 nM nocodazole for 18 h as a control. **i**. Immunoblot of SW48 and HCT116 cells treated with etoposide or camptothecin at the indicated concentrations (nM) for 24 h.

Concomitant inhibition of helicase and ATPase activity is a general feature of our series with comparable IC_50_ values across a range of protein constructs, including full-length WRN and the minimal D1–D2 helicase core (WRN 517–945) (Extended Data Fig. 2b). The biochemical IC_50_ of 100 nM for HRO761 in an ATPase assay at high ATP (20-fold *K*_M_) translated to a half-maximal growth inhibitory concentration (GI_50_) of 40 nM in a 4 day proliferation assay in SW48 cells (Fig. 2a). WRN helicase inhibition by HRO761 was sufficient to impair the viability of MSI cancer cells with GI_50_ values in the range of 50–1,000 nM, while there was no effect in microsatellite-stable (MSS) cells in a 10-to-14-day clonogenic assay. Sensitivity to HRO761 inhibition correlated with genetic dependence (for example, DRIVE score; Fig. 2b). By contrast, HRO761 binding was similar across all MSI and MSS cell lines tested and in the range of 10–100 nM half-maximal protein stabilization (PS_50_) as determined in a target engagement assay measuring WRN protein stabilization in lysates (Fig. 2c). The introduction of Cys727 knock-in mutations at the HRO761-binding site of WRN that reduce biochemical potency was correlated with significant PS_50_ reduction in two engineered models (HCT116-C727A (PS_50_ of 0.7 µM); RKO-C727S (PS_50_ 8.8 µM)) as well as impaired proliferation and pharmacodynamic (PD) modulation (Fig. 2c and Extended Data Figs. 2b and 3b). These data demonstrate that, although HRO761 binds equally to WRN in all of the cells studied, WRN inhibition leads to an anti-proliferative effect only in MSI cells. This is further supported by data from a proliferation screen in 301 cells from the Horizon OncoSignature panel (Extended Data Fig. 3a). In this panel, several MSI cells appear insensitive as they do not reach 50% growth inhibition in the 5 day assay period. We hypothesize that WRN inhibition leads to the accumulation of DNA damage over multiple cell cycles, explaining why antiproliferative effects increase with treatment duration (Supplementary Table 1).

Consistent with the selective viability effects in MSI cells, HRO761 elicited DNA damage response (DDR) in cell lines that are sensitive to WRN inhibition, but not in cells that are insensitive to WRN inhibition or *WRN* knockdown (Fig. 2d). Coincident with DNA damage, we observed p53 activation and WRN degradation only in MSI cells, and we also observed an increase in the amount of WRN bound to chromatin only in these cells (Fig. 2e). We confirmed that the loss of WRN occurs at the protein level and is not due to the loss of antibody recognition, as WRN protein is the most significantly down-modulated protein in three MSI cell lines but not modulated in an MSS cell line (Fig. 2f). WRN has been described to undergo post-translational modifications leading to ubiquitination and subsequent degradation after DNA damage and replication stress^27,28^. Drugs such as camptothecin or etoposide lead to WRN degradation and activation of the DDR irrespective of their MSI/MSS status, whereas HRO761 led to proteasome-mediated WRN protein degradation only in MSI cells (Fig. 2g and Extended Data Fig. 3c,d). Degradation of WRN as a consequence of WRN inhibition and subsequent DDR activation is supported by a partial rescue of the levels of WRN protein as well as rescue of viability after inhibition of ATM in addition to treatment with HRO761 (Extended Data Fig. 3e–g).

**Extended Data Fig. 4:**
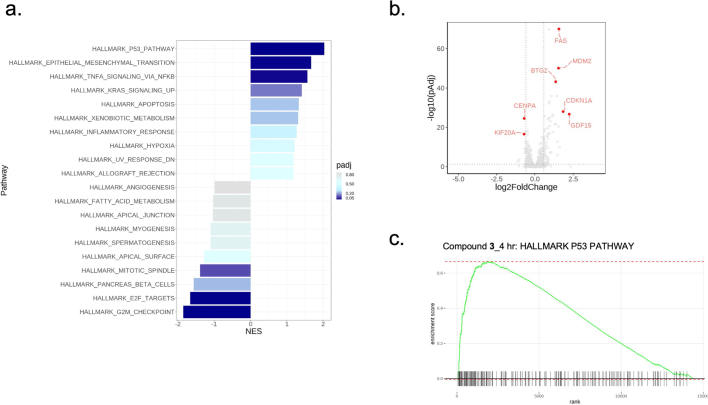
WRN inhibition induces apoptosis, a p53 response and cell cycle arrest. **a**. Results of GSEA Hallmark analysis showing the enriched gene sets from bulk RNASeq analysis of SW48 tumours from mice treated once with **3** at 150 mg/kg and sacrificed/sampled 4 h post treatment. Listed are the top 10 positively and negatively enriched gene sets based on normalized enrichment scores (NES), coloured by statistical significance. Gene sets p-values were calculated using an adaptive, multilevel splitting Monte Carlo approach implemented in the fsgea R package and adjusted for multiple hypothesis testing by the Benjamini–Hochberg method (pAdj). **b**. P values (pAdj) plotted against log_2_ fold change of expression from bulk RNASeq analysis of tumours from mice treated once at 150 mg/kg and sacrificed/sampled 4 h post treatment. Statistical significance was determined using a two-sided Wald test statistical analysis (in DEseq2), followed by Benjamini-Hochberg multiple-correction testing (n = 5 mice). **c**. Signature enrichment plot for p53 hallmark gene set. Source Data

Assessment of gene expression using RNA-sequencing (RNA-seq) analysis in four MSI cell lines treated with analogue **2** revealed modulation of the expression of multiple genes, including p53-target genes such as *CDKN1A*, *MDM2*, *BTG2*, *GDF15*, *CENPA* and *KIF20A*, while no genes were significantly modulated after treatment in MSS cell lines or *WRN*-knockout cells (Fig. 2h). Moreover, SW48 tumours treated with analogue **3** showed an enrichment of the p53 pathway in vivo and confirmed the genes identified by RNA-seq (Extended Data Fig. 4a–c).

**Fig. 3:**
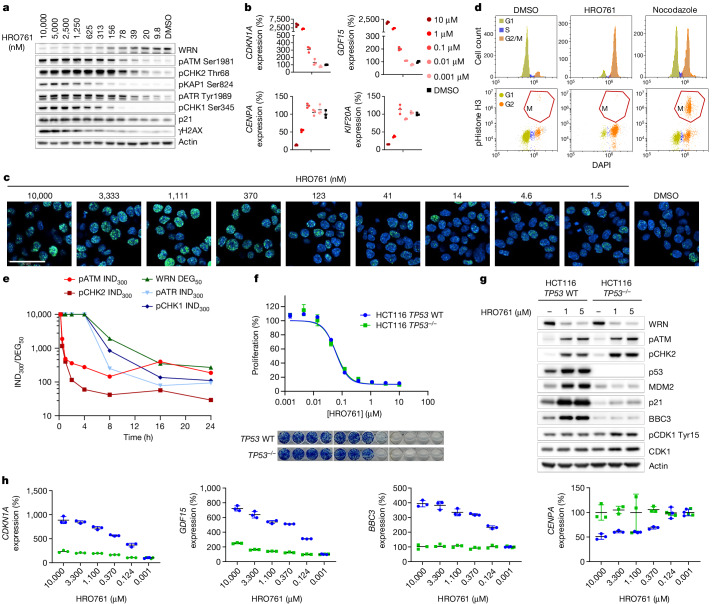
WRN inhibition leads to cell cycle arrest and DNA damage in a time- and dose-dependent manner, independently of p53. **a**, Immunoblot analysis of WRN, pATM (Ser1981), pCHK2 (Thr68), pKAP1 (Ser824), pATR (Tyr1989), pCHK1 (Ser345), p21, γH2AX and actin in HCT116 cells after 24 h of treatment with HRO761 at the concentration indicated at the top. **b**, *CDKN1A* (encoding p21), *GDF15*, *CENPA* and *KIF20A* mRNA levels were quantified by quantitative PCR with reverse transcription (RT–qPCR) in HCT116 cells treated with HRO761 as described. Data are mean ± s.d. percentage expression compared with the DMSO treatment group. *n* = 3. **c**,**d**, γH2AX immunofluorescence (**c**) and cell cycle analysis (**d**) in HCT116 cells after treatment with either DMSO or HRO761 at 10 µM for 24 h. Cell cycle analysis showing DAPI staining (top) and DAPI against phosphorylated histone H3 (bottom). Immunofluorescence data are from one representative experiment out of three. Scale bar, 50 µm. **e**, Time course of PD modulation quantified as the amount of HRO761 required to induce 300% phosphorylation (IND_300_) of DDR markers or 50% WRN degradation compared with DMSO. Western blot images are shown in Extended Data Fig. 5. **f**, Survival curves of HCT116 *TP53* wild-type (WT) or HCT116 *TP53*^*−/−*^ cells exposed to HRO761 for 5 days (top). The cell viability was estimated using the CellTiter-Glo assay. Data are mean ± s.d. percentage of surviving cells compared with DMSO treatment. *n* = 3 biological replicates. Bottom, representative image of a 14 day CFA. **g**,**h**, Immunoblot analysis of WRN, pATM (Ser1981), pCHK2 (Thr68), p53, MDM2, p21, BBC3, pCDK1 (Tyr15), CDK1 and actin (**g**) and RT–qPCR analysis of *CDKN1A*, *GDF15*, *BBC3* and *CENPA* (**h**) in HCT116 *TP53* WT (blue) or HCT116 *TP53*^*−/−*^ (green) cells exposed to HRO761 for 24 h. Data are mean ± s.d. percentage expression compared with the DMSO treatment group. *n* = 3 biological replicates.

**Extended Data Fig. 5:**
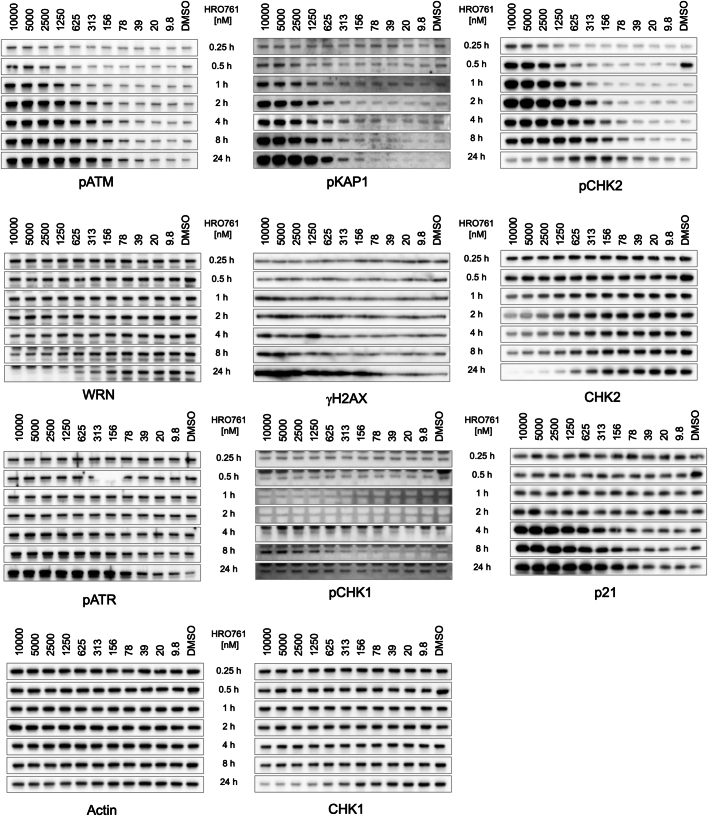
Time course of WRN degradation and DNA damage induction after HRO761 treatment. Immunoblots for WRN, pATM (S1981), pCHK2 (T68), total CHK2, phospho-KAP1 (S824), pCHK1 (S345), total CHK1, pATR (Y1989), p21, γH2AX and actin during 0.25 h to 24 h of treatment of HCT116 cells with HRO761 at the concentrations indicated. Images from a representative experiment of n = 3.

We next characterized the cellular consequences of HRO761 treatment in MSI cells in detail. In addition to a dose-dependent increase in the activation of ATM and CHK2, we observed modest but reproducible ATR activation, γH2AX foci formation, p21 protein induction, dose-dependent regulation of p53-dependent genes identified from RNA-seq and a potent G2 cell cycle arrest (Figs. 2h and 3a–d). We observed that activation of ATM and CHK2 occurred within 1 h after HRO761 treatment and WRN inhibition triggered little CHK1 activation, consistent with arrest in G2/M^29^. By contrast, WRN degradation started at 8 h and was more pronounced after 24 h of treatment. This is consistent with WRN degradation being a consequence of the DNA-damage activation^27,28^. WRN degradation was not a result of the G2 cell cycle arrest as WRN levels remained constant throughout the cell cycle (Extended Data Fig. 3h). It has been reported that CHK2 levels are also modulated after DNA damage^30^. Consistent with this, we observed downregulation of total CHK2 protein levels after HRO761 treatment (Extended Data Figs. 3i and  5). However, it is unlikely that lethality of HRO761 is due to CHK2 (or CHK1) loss, as HRO761 inhibits growth at concentrations devoid of CHK1 and CHK2 degradation (that is, 300 nM HRO761 and below).

**Extended Data Fig. 6:**
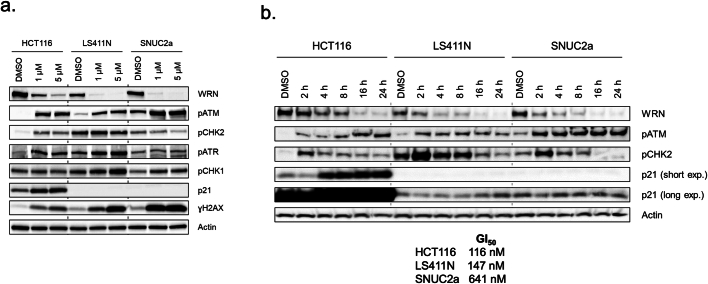
DNA damage response upon HRO761 treatment in p53 mutant cells. **a**. Immunoblot for WRN, pATM (S1981), pCHK2 (T68), pATR (Y1989), pCHK1 (S345), p21, γH2AX and actin of the indicated cells treated with compound **4** for 24 h at the concentrations indicated. **b**. Immunoblot for WRN, pATM (S1981), pCHK2 (T68), p21 and actin of the indicated cells treated with **4** at 5 µM for the time indicated. GI_50_ values after 96 h of compound **4** treatment are indicated in nM under the immunoblots.

Reanalysis of WRN dependence from DRIVE data stratifying by p53 status revealed that both wild-type and mutant-*TP53* MSI cells are dependent on WRN^2^. HRO761 treatment led to similar anti-proliferative effects in HCT116 cells with a genetic knockout at the *TP53* locus and in the parental *TP53-*WT cells (Fig. 3f). While there was no modulation of p21, GDF15, CENPA or BBC3 (also known as PUMA) in *TP53-*knockout cells (Fig. 3g,h), HRO761 treatment led to increased ATM and CHK2 phosphorylation as well as WRN degradation equally in both cell lines (Fig. 3g). Moreover, phosphorylation of CDK1 at Tyr15 was increased in the *TP53*-knockout cells (Fig. 3g). We confirmed that the effects of WRN inhibition are p53 independent in additional *TP53*-mutant cells such as LS411N and SNUC2a (Extended Data Fig. 6a,b).

**Fig. 4:**
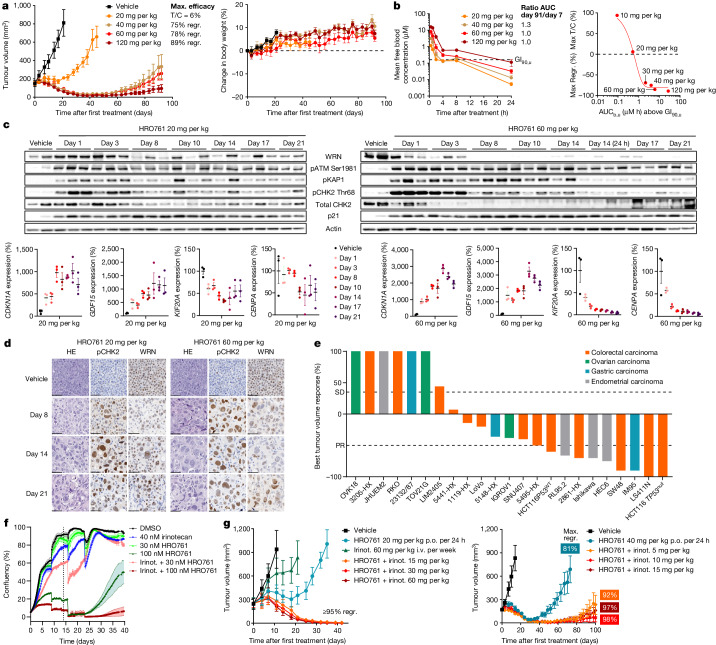
In vivo pharmacological proof of concept of WRN synthetic lethality with HRO761. **a**, The therapeutic response to HRO761 in mice bearing SW48 xenografts. Mice were treated for 92 days with vehicle (*n* = 5) or HRO761 (orally, once daily) at doses up to 120 mg per kg. Mouse body weight was measured from the treatment start. Data are mean ± s.e.m. **b**, Free HRO761 blood concentrations (*n* = 3) at day 7 and 91 (mouse unbound faction = 19.3%) (left); unbound GI_90_ (0.16 µM and in vitro unbound fraction = 37%) is represented as a dotted line. Right, the correlation between efficacy and unbound blood area under the curve (AUC_b,u_) above the SW48 cell unbound GI_90_. Data are mean ± s.e.m. (left). **c**, Immunoblot analysis of WRN, pATM (Ser1981), pCHK2 (Thr68), total CHK2, pKAP1, p21 and actin from SW48-tumour bearing mice (*n* = 3) treated daily with 20 and 60 mg per kg and euthanized and sampled 4 h after the last treatment from day 1 to day 21 (top). Bottom, *CDKN1A*, *GDF15*, *CENPA* and *KIF20A* mRNA levels were quantified by RT–qPCR. Data are mean ± s.e.m. percentage expression compared with the DMSO control group. **d**, WRN and pCHK2 expression were evaluated on SW48 xenograft FFPE sections using immunohistochemistry. Scale bars, 50 µm. Replicate information is provided in the ‘Statistics and reproducibility’ section of the Methods. **e**, Best average response from multiple MSI xenograft models (*n* = 5–7) after treatment with HRO761 at 60 or 120 mg per kg according to the model. **f**, Incucyte-generated confluence plots from SW48 cells exposed to a combination of HRO761 and irinotecan (irinot.) at the indicated concentrations. The graph is a representative experiment of *n* = 3 experiments. **g**, SW48 tumour xenograft growth of mice (*n* = 5–6) treated with HRO761 at 20 mg per kg, irinotecan (intravenous, weekly) or a combination of HRO761 with a decreasing dose of irinotecan from 60 to 15 mg per kg (left), or HRO761 at 40 mg per kg or a combination of HRO761 with a decreasing dose of irinotecan from 15 to 5 mg per kg (right). Data are mean ± s.e.m. Source Data

**Extended Data Fig. 7:**
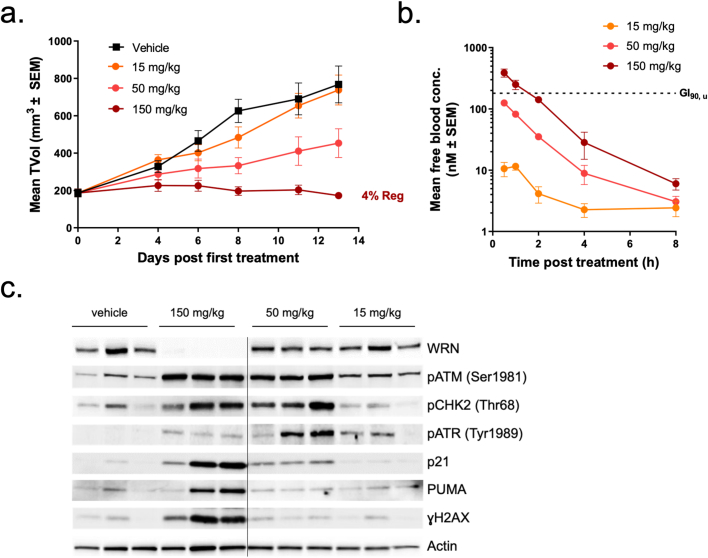
WRN inhibition induces apoptosis, a p53 response and cell cycle arrest. **a**. Xenograft growth of SW48 cells treated p.o. twice daily with compound **3** at 15, 50 or 150 mg/kg. Mice were randomized (n = 7). **b**. Mean total blood concentration of compound **3** performed on the last day of the efficacy experiment. Data are average of n = 3 mice per time point ± SEM **c**. Immunoblot for WRN, pATM (S1981), pCHK2 (T68), pATR (Y1989), p21, pH2AX (S139) and actin 3 h post-last treatment with compound **3** on the last day of the efficacy experiment (i.e., day 13 post start of treatment). Source Data

**Extended Data Fig. 8:**
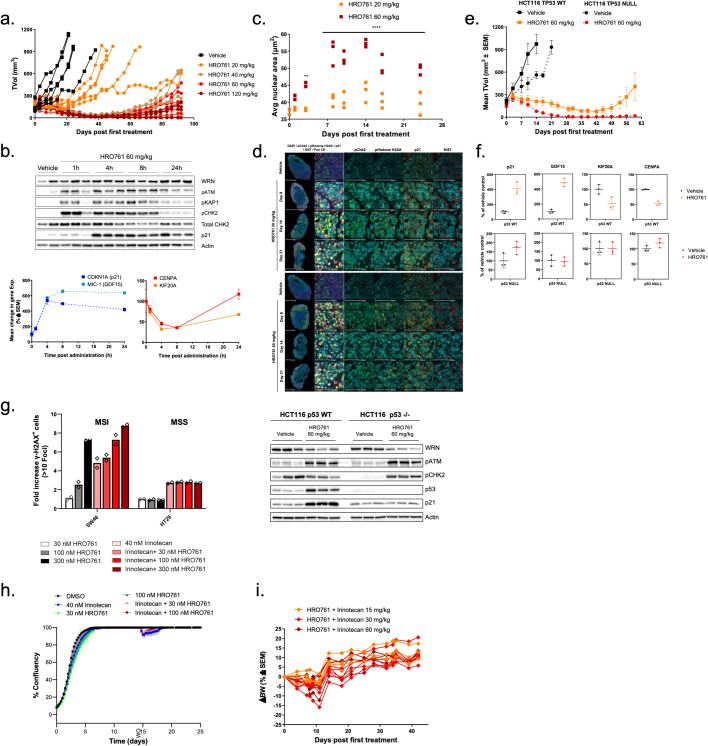
*In vivo* responses to HRO761 in p53 WT and null cells alone or in combination. **a**. Individual SW48 tumour volume data from Fig 4a. **b**. Immunoblot for WRN, pATM (S1981), pCHK2 (T68), total CHK2, phospho-KAP1 (S824), p21 and actin from SW48-tumour bearing mice (n = 3) treated at 60 mg/kg and sacrificed/sampled at 1, 4, 8 and 24 h post-treatment. Bottom panel p21, GDF15, CENPA and KIF20A mRNA levels were quantified by qRT-PCR. **c**. Average nuclear area measured in SW48 tumour sections. ** P < 0.01 and **** P < 0.0001, 2-way ANOVA with a Sidak’s multiple comparison test. **d**. DNA damage response after HRO761 treatment was evaluated on SW48 xenograft FFPE sections by Multiplex Immunofluorescence: DAPI (blue), pCHK2 (green), γH2AX (yellow), p21 (orange), Ki67 (red) and PAN CK (pale blue). **e**. Tumour xenograft growth in HCT116 TP53 WT or null models treated with HRO761 at 60 mg/kg (n = 6 mice per group). **f**. Top. qRT-PCR for p21, GDF15, CENPA and KIF20A. Data are mean of expression compared to vehicle control ± SD, n = 3 mice. Bottom. Immunoblot for WRN, pATM (S1981), pCHK2 (T68), total CHK2, pKAP1 (S824), p21 and actin from HCT116-tumour bearing mice (n = 3) treated at 60 mg/kg of HRO761 and sacrificed 4 h post second treatment. **g**. DNA damage accumulation after HRO761 treatment as SA and in combination with Irinotecan was evaluated by quantification of γH2AX foci formation on SW48 (MSI) and HT29 (MSS) cells. Bars represent the mean of 2 values and is a representative experiment (n = 2). **h**. Incucyte confluence plots from Cal33 cells treated with HRO761 and irinotecan at indicated concentrations. Graph is a representative experiment (n = 3). **i**. Body weight (BW) change (%) from the mice treated with the combination of HRO761 at 20 mg/kg and irinotecan doses from 60 to 15 mg/kg (efficacy in Fig. 4g).

We next tested WRN inhibitors in vivo in SW48-cell-derived xenografts (CDXs). Compound **3** showed a dose-dependent exposure in the blood, inducing a stable disease at 150 mg per kg given orally twice daily (Extended Data Fig. 7). Owing to its improved PK properties, once daily oral dosing of HRO761 led to tumour stasis at 20 mg per kg and led to 75–90% tumour regressions at higher doses for up to 60 days, after which tumours relapsed in a dose-dependent manner (Fig. 4a and Extended Data Fig. 8a). HRO761 did not cause toxicity as inferred by monitoring animal weight. The anti-tumour effects were associated with a dose-dependent exposure in the blood, without accumulation. Moreover, unbound blood area under the curve above unbound GI_90_ strongly correlates with efficacy and shows to be a driver of tumour growth inhibition (Fig. 4b). There was a concordant activation of the DDR pathway as well as WRN protein degradation and p53-target gene modulation, which reached a steady state by day 8 at 60 mg per kg, and some markers such as phosphorylated KAP1 (pKAP1) and pCHK2 decreased over time as tumours regressed (Fig. 4c and Extended Data Fig. 8b). Immunohistochemistry and immunofluorescence analysis of the tumours showed a decrease in Ki-67 as well as an enlargement of the cell nuclei (Fig. 4d and Extended Data Fig. 8c,d). A large-scale in vivo screen in a panel of CDX and patient-derived xenograft (PDX) models across different MSI indications resulted in a disease control rate of approximately 70%, with 35% stable diseases, 30% partial responses and 9% complete responses (Fig. 4e). Notably, the two complete responses corresponded to a p53 mutant (LS411N) and a p53-null (HCT116) CDX. The *TP53-*knockout HCT116 model had an even better in vivo response than the parental model (Fig. 4e and Extended Data Fig. 8e). In contrast to a recent report in which the authors observed that synthetic lethality of WRN was dependent on p53 activity^31^, our data demonstrate that p53 is not required for the anti-tumour activity of HRO761. As double-stranded DNA breaks have been reported to be toxic to cells independent of p53 status, this can explain why MSI cells with p53 loss are sensitive to WRN inhibition^32^.

We next assessed whether the DNA damage and anti-proliferative activity of HRO761 could be potentiated with irinotecan, an inhibitor of topoisomerase I used as standard of care and effective treatment in colorectal cancer^33^. Combination at subefficacious concentrations of each HRO761 and irinotecan led to enhanced antiproliferative activity in SW48 cells (Fig. 4f) while at higher doses of HRO761, it prevented long term cellular regrowth in vitro. These data are consistent with an increased number of γH2AX foci in the combination treatment compared with both single agents (Extended Data Fig. 8g). In vivo studies confirmed the benefit of the combination of HRO761 and irinotecan, causing a complete tumour regression, independent of the dose of irinotecan (Fig. 4g (left)) and HRO761 (Fig. 4g (right)). This combination was well tolerated, and no change in body weight was observed (Extended Data Fig. 8i).

In summary, here we report the identification of HRO761, a potent and selective WRN inhibitor that allosterically binds at the interface of the D1 and D2 helicase domains, thereby locking WRN in an inactive conformation. Inhibition of WRN by HRO761 selectively inhibits the growth of MSI cancer cells in vitro and in vivo and recapitulates the synthetic lethality of WRN in MSI cancers previously observed by genetic screens^2–6^. HRO761 induces double-stranded DNA breaks and activates the DDR to induce WRN degradation and promote cell death and cell cycle arrest selectively in MSI cells, independently of their p53 status. Collectively, our findings represent preclinical pharmacological validation of WRN as a synthetic lethal therapeutic target in MSI cancers. HRO761 has linear exposure after oral administration, displays a favourable safety profile in toxicology studies and is predicted to have a high therapeutic index with sufficient margins to support clinical development. HRO761 is currently undergoing a first in human clinical trial to assess the safety, tolerability and preliminary anti-tumour activity in patients with MSI colorectal cancer and other MSI solid tumours (NCT05838768).

## Methods

### Chemistry

Compound **1** (ZINC21803075, D475-1631) was purchased from ChemDiv, and the synthesis of compounds **2**–**6** is described in the Supplementary Information. Etoposide and camptothecin were purchased from Sigma-Aldrich (E1383 and 208925). ATM inhibitors KU-55933 and AZD1390 were purchased from SelleckChem^34^.

### Co-crystal structures of WRN helicase domain with ATPγS, compound **3** or HRO761

Two constructs comprising the core helicase domain of human WRN (amino acids of 517–945), with either five (hWRN_mut5: E625A, R716A, K804A, E886A, R942A) or six (hWRN_mut6: R564A, E625A, R716A, K804A, E886A, R942A) point mutations were cloned with an N-terminal hexa-histidine tag and ZZ tag followed by a PreScission protease cleavage site for recombinant baculovirus generation. The surface mutations were introduced to facilitate crystallization of the WRN helicase domain^35^. Protein expression was done in virus infected *Spodoptera frugiperda* (Sf9) cells. Cells were incubated with shaking at 120 rpm, 27 °C and collected 72 h after infection by centrifugation at 500*g* for 20 min at room temperature. Cell pellets were flash-frozen and stored at −80 °C.

For protein purification, the cell pellet was thawed in lysis buffer containing 50 mM NaH_2_PO_4_ pH 7, 300 mM NaCl, 20 mM imidazole, 10% glycerol, 1 mM TCEP, 250 U of Benzonase and cOmplete EDTA-free protease inhibitor cocktail. Cells were lysed manually using a Dounce homogenizer and the lysate was cleared by centrifugation in a SS34 rotor at 14,000 rpm for 30 min at 4 °C followed by filtration using 5 µm, 1.2 µm and 0.45 µm Acrodisc filters. The cleared lysate was subjected to immobilized metal affinity chromatography (IMAC) using a prepacked HisTrap HP column in IMAC buffer A (50 mM NaH_2_PO_4_ pH 7, 300 mM NaCl, 20 mM imidazole, 10% glycerol, 1 mM TCEP). The column was washed with 5 CV 10% buffer B (50 mM NaH_2_PO_4_ pH 7, 300 mM NaCl, 300 mM imidazole, 10% glycerol, 1 mM TCEP) and the protein was eluted applying a linear gradient over 10 CV to 100% buffer B. The fractions containing WRN were incubated with PreScission protease at 4 °C overnight. After cleavage, the products were diluted 1:3 in 50 mM Tris pH 7,0, 10% glycerol and applied to a prepacked HiTrap Heparin HP column to remove DNA that co-fractionates with WRN. The column was washed with 2 CV loading buffer and the protein was eluted from the column by applying a salt gradient from 0% to 100% elution buffer (50 mM Tris-HCl pH 7, 1 M NaCl, 10% glycerol) in 25 CV. The WRN-containing fractions were pooled and concentrated to about 5 mg ml^−1^ for crystallization trials. The purified samples were aliquoted, flash-frozen and stored at −80 °C.

All crystallization trials were performed using the sitting-drop vapour-diffusion method at room temperature. Crystals of the ATPγS complex were obtained by soaking. Apo crystals were grown by mixing equal amounts (0.4 µl) of hWRN_mut5 at 5 mg ml^−1^ and reservoir solution containing 20% PEG3350, 0.2 M sodium citrate tribasic dihydrate. ATPγS was dissolved in reservoir solution together with MgCl_2_ at 2 mM concentration and the crystals were soaked overnight. Before flash-cooling the crystals in liquid nitrogen for X-ray data collection, they were placed in reservoir solution containing 30% glycerol for a few seconds.

Crystals of the complex with compound **3** and HRO761 were obtained by co-crystallization at 293 K using the sitting-drop vapour-diffusion method. hWRN_mut6 at 5.4 mg ml^−1^ in 50 mM Tris pH 7, ~300 mM NaCl and 10% glycerol was mixed with inhibitor **3** or HRO761 (10 mM in DMSO) to reach a final compound concentration of 1 mM. Equal volumes (0.4 µl) of protein complex and reservoir solution were mixed, and the plates were incubated at room temperature. Compound **3** complex crystals were obtained using 30% Jeffamine ED-2001 (Hampton Research) and 0.1 M HEPES pH 7 as reservoir solution. HRO761 complex crystals grew using 25.5% PEG4000, 0.17 M ammonium sulfate and 15% glycerol as reservoir solution. Single crystals appeared after a few days and were soaked for 10 s in mother liquor containing 20% glycerol and 10 mM inhibitor compound before flash-cooling them in liquid nitrogen for data collection.

X-ray diffraction data were collected at the Swiss Light Source, beamline X10SA and processed using XDS/XSCALE^36^ and the autoPROC/STARANISO^37,38^ toolbox (Extended Data Table 2). Analysis by STARANISO revealed that diffraction data were anisotropic for the HRO761 complex, with estimated diffraction limits for reciprocal directions of 1.86 Å along a*, 2.12 Å along b* and 1.87 Å along c*. Accordingly, this dataset was processed anisotropically, which explains the lower spherical completeness. The structures were solved by molecular replacement with PHASER^39^, using as a search model the coordinates of previously solved in-house structures of WRN. The first in-house structure of WRN was solved by molecular replacement using as a search model the coordinates of the D1D2 subdomains of the published BLM helicase structure (PDB: 4O3M)^40^. The software programs COOT^41^ and BUSTER^42^ were used for iterative rounds of model building and structure refinement. The refined coordinates of the complex structures have been deposited at the PDB. Data collection and refinement statistics are summarized in Extended Data Table 2.

### Human WRN (D1D2RH) expression and purification

A DNA sequence enabling expression of human WRN-D1D2RH (UniProt: Q14191, Asn517–Pro1238), fused at the amino-terminal end to His tag followed by ZZ tag and HRV 3 C cleavage site, was inserted into a baculovirus transfer vector compatible with the co-transfection virus generation approach. The corresponding baculovirus stocks were generated in Sf21 cells, amplified once and frozen according to the Titerless Infected-Cells Preservation and Scale-up protocol (TIPS)^43^. Large-scale expression was conducted in Sf21 cells at 27 °C for 96 h. Cells were collected and resuspended in lysis buffer (50 mM Tris, 300 mM NaCl, 1 mM TCEP, 10% glycerol, pH 8) supplemented with cOmplete protease inhibitor tablets (Roche), benzonase (Merck) and 20 mM imidazole. Cells were lysed with an Emulsiflex C3 homogenizer (Avestin). After ultracentrifugation, the lysate was loaded onto HisTrap HP column (Cytiva). Bound protein was eluted with imidazole gradient in lysis buffer. The amino-terminal His and ZZ tags were cleaved by HRV 3C protease during overnight dialysis against dialysis buffer (50 mM Tris, 150 mM NaCl, 1 mM TCEP, 10% glycerol, 0.02% CHAPS, pH 8). Human D1D2RH was further purified by ion exchange. The protein was loaded onto the Resource S ion-exchange column (Cytiva) and eluted with a NaCl gradient in IEX buffer (20 mM Tris, 1 mM TCEP, 10% glycerol, pH 7.0). As a final step, the protein was concentrated and loaded onto a Superdex 200 gel-filtration column (Cytiva) pre-equilibrated with SEC buffer (50 mM Tris, 150 mM NaCl, 10% glycerol, pH 7.4). Eluted target protein was concentrated and frozen on dry ice.

### Human BLM-D1D2RH, human RecQ1 and human RecQ5 expression and purification

DNA sequence allowing expression of human BLM-D1D2RH (UniProt P54132: Lys640–Gly1298) fused at the amino-terminal end to His tag followed by TEV-cleavage site, was inserted into a pET-derived vector. Protein expression was performed in BL21(DE3) cells after IPTG induction. Cells were collected and resuspended in lysis buffer (50 mM NaH_2_PO_4_, 300 mM NaCl, 1 mM TCEP, 20 mM imidazole, pH 8.0) supplemented with 2 mM MgCl_2_, benzonase and 0.5 mM AEBSF. Cells were lysed with an Emulsiflex C3 homogenizer. After ultracentrifugation, the lysate was loaded onto a HisTrap HP column. Bound protein was eluted with an imidazole gradient in lysis buffer. The amino-terminal tag was cleaved with TEV protease. Cleaved protein was loaded on Resource S ion-exchange column and eluted with a NaCl gradient in IEX buffer (20 mM Tris, 1 mM TCEP, 10% glycerol, 20 mM NaCl, pH 7.4). As a final step, BLM-D1D2RH protein was concentrated and loaded onto the Superdex 75 gel-filtration column pre-equilibrated with SEC buffer (50 mM Tris, 150 mM NaCl, 10% glycerol, pH 7.4). Eluted target protein was concentrated and frozen on dry ice. An analogous procedure was used for the expression and purification of human RecQ1-D1D2 (UniProt: P46063, Asp62–Ser482) and Human RecQ5-D1D2 (UniProt: O94762, Asp11–Trp453) proteins.

### Cy5-ATPgS binding assay using TR-FRET

A total of 9.8 μl N-terminal Avi Tag-WRN(Asn517–Pro1238) in 30 mM Tris-HCl pH7.5, 1 mM MgCl_2_, 30 mM NaCl, 0.02% BSA, 0.1% Pluronic F127 was incubated with 200 nl compound for 45 min at room temperature in white 384-well plates (Greiner Bio-One, 781207). Then, 10 μl Cy5-labelled ATPγS (EDA-ATPγS-Cy5, Jena Bioscience, NU-1609-CY5) and europium-labelled Streptavidin (LANCE Eu-W1024 Streptavidin, Perkin Elmer, AD0063) were added. The final concentrations in the assay plate were 15 nM WRN, 10 nM ATPγS-Cy5 and 2 nM Eu-Streptavidin. With the TECAN infinite M1000 PRO, Eu was excited at 340 nm and the fluorescence emission was measured at 620 nm (*F*_620_) and 665 nm (*F*_665_) after 30 min. A ratio of *F*_665_ over *F*_620_ was then calculated to determine the FRET efficiency in each well. This assay protocol was used during hit optimization to build the initial structure–activity relationship and was also adapted to 1,536-well plates for screening purposes.

### ATPase activity analysis of WRN helicase

We used an ADP-Glo assay kit (Promega) to monitor ATP hydrolysis^44^. A 45-oligonucleotide with the following sequence: TTTTTTTTTTTTTTTTTTTTTTCCAAGTAAAACGACGGCCAGTGC, referred to as FLAP26, as described previously^45^ was purchased from IDT (Integrated DNA Technologies) and used as a DNA substrate. A typical reaction consisted of 10 nM WRN D1D2RH (protein construct (Asn517–Pro1238)), 0.2 nM single-stranded DNA (ssDNA) substrate (*K*_DNA_) and 15 or 300 µM ATP (20-fold *K*_M_) in the following assay buffer: 30 mM Tris pH 7.5, 2 mM MgCl_2_, 0.02% BSA, 50 mM NaCl, 0.1% Pluronic F127 prepared in DNase-free water. For IC_50_ determination, 10 half-log dilutions were prepared in DMSO from a 10 mM compound solution. Then, 50 nl of each concentration was preincubated for 3 h in a 384 small volume assay plate (Greiner, 784075) with 2.5 μl of a 20 nM WRN helicase protein in assay buffer with 600 µM ATP. The reaction was started by addition of 2.5 μl of ssDNA substrate at 0.4 nM and incubated for 30 min at room temperature. The reaction was stopped with the addition of 5 μl of the first ADP-Glo reagent and incubated for 1 h to remove the excess amount of ATP. Then, 10 μl of ATP detection reagent was added and incubated for an additional hour before reading. The luminescence output was recorded using the Pherastar reader, with a 5 min delay before reading. Each concentration of compounds was tested in duplicates in the assay plate. The compound potency (IC_50_ value) was determined using custom software (Novartis Helios software application) with a four-parameter sigmoid Hill-curve model^46^. After normalization of activity values for the wells to percentage inhibition (percentage inhibition = [(high control activity − sample activity)/(high control activity − low control activity)] × 100), IC_50_ fitting was performed from the duplicate determinations present on each plate.

### RecQ selectivity assays on RecQ1, BLM and RecQ5

The same ATPase assay protocol was used with the three other RecQ protein constructs to assess the selectivity over WRN. Time-course experiments were performed to determine the best enzymatic assay conditions for each RecQ protein. The experiments were run as described above with 10 nM RecQ1 protein construct with 10 nM ssDNA substrate, 2 nM BLM protein construct with 0.2 nM ssDNA substrate and 0.5 nM RecQ5 protein construct with 2 nM ssDNA substrate, respectively.

### Quenched fluorescence DNA-unwinding assay

We used the same DNA substrate as described previously^45^ with slightly different dyes. The FLAP26 oligonucleotide was labelled with an Atto647 dye in 3′ and annealed with the TSTEM25 oligonucleotide carrying a BHQ3 dark quencher in 5′. The Atto647/BHQ3 pair of fluorescent dye/quencher (excitation 620 nm, emission 685 nm) was selected for a better assay robustness and lower interference with the compounds. Then, 4.9 μl WRN D1D2RH in 30 mM Tris-HCl pH 7.5, 0.5 mM MgCl_2_, 50 mM NaCl, 0.02% BSA, 0.1% Pluronic F127 and 1 mM DTT were incubated with 100 nl compound for 45 min at room temperature in black 384-well plates (Greiner Bio-One, 784076). Next, 5 μl of a solution containing ATP, double-stranded DNA (dsDNA) as described above and trap ssDNA TSTEM01 were added to initiate the reaction. The final assay concentrations were 0.5 nM WRN (D1D2RH), 300 μM ATP, 50 nM dsDNA and 100 nM trap ssDNA (GCACTGGCCGTCGTTTTACG). The increase in fluorescence after DNA unwinding was monitored over time using the TECAN infinite M1000 PRO system. The excitation and emission wavelengths were set to 647 nm and 669 nm, respectively.

### Radioactive binding assay using an SPA

A total of 20 μl N-terminal Avi tag-WRN protein (Asn517-Pro1238) in 30 mM Tris pH 7.5, 30 mM NaCl, 1 µM MgCl_2_, 0.02% BSA, 0.1% Pluronic F127, containing or not a fivefold higher concentration of dsDNA as compared to protein concentration, were incubated with 20 μl [^3^H]-probe **6** (Extended Data Fig. 2f) for 1 h at room temperature. Then, 10 μl Streptavidin-coated PVT scintillation proximity assay (SPA) beads (Perkin Elmer, RPNQ0009) were added and the plate was read after 30 min in a TopCount luminescence reader (1 min, delay,1 min per well). The final assay concentrations were 10 nM WRN, 50 nM dsDNA (for the condition with dsDNA), a concentration range of radioligand as shown in the graphs and 50 μg per well of PVT SPA beads. The dsDNA was the same substrate as used in the DNA-unwinding assay (without labels).

### Cell lines and reagents

All cell lines are part of the original Cancer Cell Line Encyclopedia and were handled as described previously^1^. Cell line media and origins are described in Supplementary Table 6. All models were regularly tested for being free of mycoplasma and their identity was verified by SNP genotyping. HCT116 *TP53*^*−/−*^ cells were bought from Horizon (D104-001). DLD-1 *WRN*-knockout cells were generated using standard CRISPR techniques and the following sgRNA sequence from GenScript AGCATCGAACTATACACAA^47^. The *WRN*-knockdown insensitive colon carcinoma cell line DLD-1 (RRID: CVCL_0248) was obtained from the Korean Cell Line Bank and used to generate a derivative in which the endogenous *WRN* gene copies were knocked out by CRISPR-mediated editing using standard CRISPR methods. This cell line is available on request under a material transfer agreement with Novartis. HCTT116 cells with a knock-in of alanine instead of cysteine at position 727 of the WRN protein were generated using standard CRISPR techniques and the following sgRNA sequence for cutting AATCTCAGATCACCTGTAC, and the following ssDNA sequence as a donor GGAAAATCGTTCTAAATCTAATCTCAAAACACAAAAACAAATTCGAAA, both from GenScript. This cell line is available on request under a material transfer agreement with Novartis. RKO cells with a knock-in of serine instead of cysteine at position 727 of the WRN protein were generated by Horizon Discovery Limited using X-MAN technology under project number CLPP775. This cell line is available from Horizon Discovery. All cells were maintained at 37 °C in a humidified 5% CO_2_ incubator.

### Generation of inducible shRNA constructs, viral production and infection

The shRNA construct cloned into the pRSI-U6-tet-ccdB inducible vector system was ordered from Cellecta. The shRNA *WRN* target sequence is CCTGACTCCAGAGGTTCAGAA corresponding to WRN gene entry NM_000553.6. The non-targeting control shRNA sequence was GGATAATGGTGATTGAGATGG. *WRN* wild-type and point-mutant cDNA expression constructs were synthesized as codon-optimized cDNAs for shRNA resistance (DNA2.0) and cloned into a lentiviral cDNA expression vector driven by a CMV promoter (pD2528-CMV).

HEK293FT cells were transfected with mixes containing 1 µg of plasmid of interest and 1 µg of Lentiviral Packaging Plasmid Mix (Cellecta, MSPPCPCP-K2A) pre-incubated for 20 min with TransIT-LT1 Transfection Reagent (Mirus, NOVSMIR2300). Transfected HEK293FT cells were incubated at 37 °C for 48 h and the supernatants containing lentivirus were collected using a 0.45 µm syringe filter. Cell infection was performed by centrifugation with fresh medium containing lentiviral supernatant supplemented with 8 µg ml^−1^ of polybrene final concentration (Merck, TR-1003-G). Cells were selected 48 h after the infection with 1 µg ml^−1^ of puromycin (Thermo Fisher Scientific, A1113803) for the shRNA constructs or 10 µg ml^−1^ blasticidin for the rescue constructs. After ensuring that the mock was dead, cells were used for further experiments and shRNA expression was induced using 100 ng ml^−1^ final concentration of doxycycline (Sigma-Aldrich, D9891) for 48 h.

### MS proteomics analysis

Cells were treated for 24 h with compound **3**, after which they were washed with ice-cold PBS, lysed using 400 µl of urea lysis buffer (8 M urea and 50 mM Tris pH 8.4) on ice and stored at −80 °C until further processing. Cell lysates equivalent to 1 million cells were reduced with 5 mM DTT for 1 h, alkylated with 10 mM iodoacetamide for 1 h and finally quenched with 10 mM DTT for 15 min. All of the steps were performed at room temperature. Subsequently, the cell lysates were diluted to 2 M urea and a trypsin/Lys-C mix (Promega) was added to an enzyme to protein ratio of 1:50 by weight followed by an overnight incubation at 37 °C. The resulting peptide mixtures were desalted on 1 ml SepPak columns (Waters), dried and labelled using the TMT 16-plex kit (Thermo Fisher Scientific) according to the manufacturer’s instructions. After verifying complete TMT labelling, the samples were pooled and fractionated into 24 concatenated fractions using high-pH reversed-phase chromatography. The 24 fractions were then analysed using an MS3-based workflow^48^ for TMT quantification on an Orbitrap Fusion Lumos LCMS system (Thermo Fisher Scientific) using a 3 h gradient. Raw data were analysed using Proteome Discoverer 2.4 (Thermo Fisher Scientific). For quantification, unique peptide intensities were summed for each protein and median-normalized protein intensities were compared across samples. Owing to the heterogenous protein levels across the three sensitive cell lines, paired *t*-tests (as implemented in the Python Scipy package) were used to calculate significant differences between DMSO-treated versus compound-**4**-treated cells.

### Cell-viability assay

The ability to inhibit cell proliferation and survival was assessed across a diverse panel of cancer cell lines. After filtration through a Steriflip-NY 0.22 μm filter (Millipore, SCNY00020), trypsinized cells were seeded at 500–8,000 cells per well into white, clear-bottom 96-well plates (Costar, 3903). Three replicate plates were prepared for each compound treatment condition. Furthermore, one plate (termed day 0) was prepared to quantify the number of viable cells at the time of compound addition. After overnight incubation at 37 °C in a humidified 5% CO_2_ atmosphere, eight threefold serial dilutions of a given compound stock (obtained at a concentration of 10 mM in DMSO and stored at 4 °C) were dispensed directly into each of the triplicate assay plates using a HP 300D non-contact Digital Dispenser (HP). The final concentration of DMSO was normalized to 0.1% in all wells. Then, 96 h after compound addition, the cellular ATP level as a surrogate for cell viability was assessed after addition of 50 µl CellTiter-Glo (Promega,G7573) reagent and luminescence quantification on the SYNERGY HT multi-mode plate-reader (BIOTEK) after a 10 min incubation at room temperature. The number of viable cells in the day 0 plate was quantified identically on the day of compound addition. For data analysis, the assay background signal that was determined in wells containing medium, but no cells, was subtracted from all other datapoints before further calculations. The extent of growth inhibition and potential cell kill was assessed by comparing the ATP levels (measured using CellTiter-Glo, Promega) in compound-treated cells with those present at the time of compound addition. To this end, the following conditional concept was programmatically applied in HELIOS, an in-house software applying a multistep decision tree to arrive at optimal concentration response curve fits^46^ to calculate the percentage growth (%G) for each compound-treated well: %*G* = (*T* − *V*_0_)/*V*_0_)) × 100 when *T* < *V*_0_, and %*G* = (*T* − *V*_0_)/(*V* − *V*_0_))) × 100 when *T* ≥ V_0_, where *V*_0_ is the viability level at time of compound addition, and *V* and *T* represent vehicle-control and compound-treated viability levels, respectively, at the end of the compound incubation. 100%, 0% and −100% signify the absence of growth inhibition, growth stasis and complete cell killing, respectively. Compound concentrations leading to GI_50_ and residual cell viability at the highest tested compound concentration (Data (*c*_max_), expressed as a percentage) were routinely calculated. Data analysis can also be carried out using commercially available software designed to derive IC_50_ values using four-parameter fits (such as GraphPad Prism, XL fit). The reported GI_50_ values are the geometrical means of at least two independent replicates. For the 301 cell lines profiled at Horizon Discovery (the Horizon - 2D OncoSignature panel), the antiproliferative activity of HRO761 was assessed through Horizon’s custom drug-screening service in 5 day CellTiter-Glo cell viability assays. Growth inhibition (GI_50_) metrics were derived from dose–response curves fitted to experimental data points capturing sensitivity to HRO761 relative to DMSO vehicle control over a nine-point dilution range up to 10 µM).

### Long-term proliferation assay using Incucyte

On day 1, cells were trypsinized, resuspended in the respective medium and counted using the TC20 cell counter from Bio-Rad. Cells were seeded in 200 μl growth medium at 3,000 to 4,000 cells per well into white, clear-bottom 96-well plates (Corning Cat, 3903). On day 2, cells were incubated overnight at 37 °C in a humidified 5% CO_2_ atmosphere, and then treated in triplicate with the indicated concentrations, using the HP 300D non-contact Digital Dispenser (TECAN). The final concentration of DMSO was normalized to 0.1% in all wells. On day 7 of the experiment, compound treatment was refreshed by carefully removing the medium by aspiration and adding fresh medium, followed by compound dosing as on day 2. Compound treatment was removed around day 15 by carefully aspirating the medium, washing once with fresh medium and adding 200 µl of fresh medium. At around day 21, the medium was refreshed by carefully aspirating the medium and adding 200 µl of fresh medium. The experiment was monitored, and images were acquired using the Incucyte S3 live-cell analysis instrument (Sartorius). Images were captured every 6 h from day 2 up to 40 days. Data were analysed and represented using GraphPad Prism.

### Clonogenic assay

Cells were seeded at 250–2,000 cells per well in a 12-well plate in 1 ml of medium. HRO761 was added at a starting concentration of 10 µM. After overnight incubation at 37 °C in a humidified 5% CO_2_ atmosphere, ten threefold serial dilutions of a given compound stock (obtained at a concentration of 10 mM in DMSO and stored at 4 °C) were dispensed directly into each assay plates using a HP 300D non-contact Digital Dispenser (TECAN). The final concentration of DMSO was normalized to 0.1% in all wells. Cells were left in the incubator for 8–20 days, with medium exchange every 3–4 days. After this time, 100 µl formaldehyde 37% was added directly in each test well and incubated for 15 min at room temperature. After rinsing twice with 5 ml of water, 0.5 ml of 0.05% methylene blue was added for 20 min at room temperature. Wells were rinsed three times with water and 1 ml of 3% HCl was added to the plates and shaken until complete colour dissolution. A total of 200 µl of this solution was transferred in a 96-well plate and the absorbance was measured at 650 nM using microtitre plate reader (Synergy HT). Compound concentrations leading to half-maximal growth inhibition (GI_50_) were calculated using XLfit using the Dose Response One Site model 201, with fit = (*A* + ((*B* − *A*)/(1 + ((*x*/*C*)^*D*^)))). For non-adherent cell lines, cellular ATP levels as a surrogate for cell viability were assessed after addition of 200 µl of CellTiterGlo (Promega, G7573) reagent to the culture after removal of 600 µl. Luminescence quantification was performed on the Synergy HT plate-reader after a 15 min incubation at room temperature. Data were analysed as for the methylene blue stain.

### Immunoblotting

Cells were lysed in NP-40 lysis buffer (0.05 M Tris HCl pH 7.8, 1% NP40, 0.12 M NaCl, 0.025 M NaF, 0.04 M β-glycerophosphate disodium salt) supplemented with complete protease inhibitor cocktail (Roche, 04906837001) and a phosphatase inhibitor cocktail (Roche, 04693124001). The lysates were fractionated in 4–12% Bis-Tris gels (Bio-Rad), which were then transferred to nitrocellulose membranes (Amersham, 10600002) and blocked for 1 h with 5% skimmed milk (Sigma-Aldrich, 70166-500) diluted in 1× PBS. The primary antibodies and the dilutions used for immunoblotting were as follows: ATM (Cell Signaling Technology, 2873, 1:1,000), pATM Ser1981 (Abcam, ab81292, 1:1,000), CHK2 (Cell Signaling Technology, 3440, 1:1,000), pCHK2 Thr68 (Cell Signaling Technology, 2197, 1:1,000), p53 (Calbiochem, OP43, 1:2,000), p21 (Cell Signaling Technology, 2947, 1:2,000), WRN (Millipore, MABD34, 1:1,000), actin (Chemicon, MAB1501, 1:10,000), pH2AX Ser139 or γH2AX (Cell Signaling Technology, 9718, 1:1,000), pCHK1 Ser345 (Cell Signaling Technology, 2348, 1:1,000), pKAP1 Ser824 (Cell Signaling Technology, 4127, 1:1,000), pATR Thr1989 (Cell Signaling Technology, 30632, 1:1,000), MDM2 (Calbiochem, OP46, 1:1,000), tubulin (Sigma-Aldrich, T9026, 1:10,000), PUMA (Cell Signaling Technology, 12450, 1:1,000), SP1 (Cell Signaling Technology, 9389), phosphorylated histone H3 (Cell Signaling Technology, 9706), cyclin E (Upstate, 06-134), cyclin A (Sigma-Aldrich, C4710), cyclin B1 (Cell Signaling Technology, 55506), goat anti-rabbit (Cell Signaling Technology, 7074, 1:5,000), goat anti-mouse (Cell Signaling Technology, 7076, 1:5,000). Membranes were incubated overnight with the primary antibodies and for 1 h with the secondary antibodies. Washing steps were performed with 1× PBS containing 0.01% Tween-20. The developing was performed using ECL substrate (Advansta, K12045-D20) and Femto substrate (Thermo Fisher Scientific, 34096) and conducted on the fusion FX device (Witec) with the EvolutionCapt software. We performed the immunoblots three times except for blots shown in Figs. 2e and 4c and Extended Data Figs. 6b and 8f, which were performed twice. Representative results from one experiment are shown. Uncropped and unprocessed scans of all blots are available as a Supplementary Figs. 1 (main text figures, which contain Figs. 2d,e, 3a,g and 4c) and 2 (Extended Data Figs. 1d,h,i, 3b–e,h,i, 5, 6a,b, 7c, 8b,g) in the Supplementary Information.

### Protein-stabilization assay

Cells were cultured in a 225 cm^2^ flask (Falcon, 353138) to a maximum confluency of 95%, then cells were rinsed twice in PBS and lysed in 12.5 ml of lysis buffer per flask (50 mM Tris pH 7.8, 1% NP40 (Sigma-Aldrich, 74385), 120 mM NaCl (Sigma-Aldrich, 71380), 25 mM NaF (Merck, 1.06450.0025), 40 mM β-glycerophosphate disodium salt (Sigma-Aldrich, 50020), 100 µM sodium metavanadate (Sigma-Aldrich, 590088), 1 mM DL-DTT (Sigma-Aldrich, 43815), 100 µM phenylmethyl sulfonyl fluoride (Sigma-Aldrich, P-7626), 1 mM benzamidine (Sigma-Aldrich, B-6506), 1 µM microcystin (Alexis Biochemicals 350-012-M001)). The lysates were centrifuged at 4 °C for 10 min and total protein was quantified and adjusted to 0.5 µg µl^−1^. Then, 100 µl of cell lysates was then plated in 96-well plates, the lysates were treated with HRO761 starting at 10 µM using the HP Dispenser (software D300eControl) and then incubated for 72 h at 20 °C. After incubation, the plates were used for in WRN enzyme-linked immunosorbent assay (ELISA) analysis as described below. PS_50_ values are the levels of HRO761 needed to stabilize the WRN protein 50% over DMSO control.

### ELISA

A list of all of the antibodies is provided in Supplementary Table 2. Maxisorp plates (Nunc, 437111, Thermo Fisher Scientific) were coated with 50 µl of primary antibody per well diluted in PBS and incubated for 2 h at room temperature. The plates were washed in PBS and 200 µl per well of blocking reagent added (PBS, 0.05% Tween-20, 5% skimmed milk), and the plates were incubated for a minimum of 2 h at room temperature. The plates were washed and 50 µl of HRO761-treated lysates (from the protein-stabilization assay) was added and incubated for 2 h at room temperature. The plates were then washed and 50 µl of secondary antibody per well diluted in blocking buffer was added and the plates were incubated overnight at 4 °C. The next day, 50 µl of tertiary antibody was added per well buffer and the plates were incubated for 2 h at room temperature. After three wash steps with 200 µl PBS with 0.05% Tween-20 and once with water, detection was then performed with 100 µl of an equal volume of Supersignal Elisa picoluminol Enhancer and Supersignal Elisa pico stable peroxide solution (Supersignal from Thermo Fisher Scientific, 37069). The plates were shaken and luminescence was detected immediately on the Synergy HT microplate reader.

### RNA preparation

Total RNA was extracted with RLT buffer (Qiagen) from SW48 cells seeded at 450,000 cells treated in six-well plates for 24 h at the indicated concentrations. Extractions were performed using the RNeasy mini kit (Qiagen, 74106) and the Qiashredder column (Qiagen, 79656) according to the manufacturer’s instructions. Total RNA concentration was measured using the Nanodrop (Thermo Fisher Scientific) device and 20 ng was used for RT–qPCR reactions.

### RT–qPCR

Reactions were performed using the iTaq Universal Probes One Step kit (Bio-Rad, 1725141) in biological and technical triplicates using RNA samples stored at −80 °C before use. A multiplexing protocol was used and TaqMan probes used for RT–qPCR analysis were ordered from iDT (https://eu.idtdna.com/) as predesigned PrimeTime qPCR Probe assays, labelled FAM/ZEN/IBFQ for all except for housekeeping gene *ACTB* TET/ZEN/IBFQ (Supplementary Table 3). In brief, 2 µl of RNA normalized at 10 ng µl^−1^ in ultrapure water (Invitrogen, 10977-035) was mixed with 8 µl of 2× master mix made as follows: iTaq mix 5 µl, 0.25 µl iScript RT, 0.25 µl TaqMan probe for gene of interest 40×, 0.25 µl TaqMan probe for control gene and 2 µl ultrapure water. The protocol for the 7900HT Device (Applied Biosystem) includes a 10 min step at 50 °C; 3 min inactivation at 95 °C; 40 cycles of denaturation of 10 s at 95 °C and 30 s hybridization/elongation step at 60 °C. The relative levels of mRNA expression were calculated according to the ΔΔ*C*_t_ method^49^ and individual expression data were normalized to *ACTB*. WRN endogenous versus WRN exogenous K577A mutant mRNA were measured by SYBR green. After isolation of RNA as described above, RT was performed with an initial amount of 250 ng of RNA using the High-Capacity cDNA Reverse Transcription Kit (Applied Biosystems, 4368813). The qPCR reaction was performed using SYBR green (Applied Biosystem, 4367659) mixed with 5 ng of cDNA and primer pairs (*WRN*endogenous forward: GTCATGGCAACTGGATATGGA, *WRN*endogenous reverse: CTGGAGCAGGCCCATGTTAC, *WRN*^*K577A*^ forward: AGGAAAGACGGGACAACGTC, *WRN*^*K577A*^reverse: CGTCGACGGCAATCAGAGTA) according to the supplier recommendations. The qPCR reaction and dissociation curve have been run on an Applied Biosystem 7900HT device. Software for analysis is SDS2.4.1, thresholds for calculation were manually adjusted and the resulting data were analysed using the Δ*C*_t_ method: for each sample, three replicates were performed for each gene of interest (as well as for the reference gene (human *ACTB*). The mean and s.d. were calculated for each group of triplicates. The Δ*C*_t_ for each sample was calculated as the difference between the *C*_t_ of the gene of interest and the reference gene for a given sample. The s.d. of this difference equals the square root of the sum of the squared s.d. of the individual mean *C*_t_. The ΔΔ*C*_t_ is the difference between the Δ*C*_t_ of the sample of interest (for example, treated) and the Δ*C*_t_ of the reference samples (vehicle-treated sample). Same as seen previously, the s.d. of this difference equals the square root of the sum of the squared s.d. of the individual Δ*C*_t_. Differential expression is calculated as ${2}^{-\Delta \Delta {C}_{{\rm{t}}}}$ for each sample. The error of the ${2}^{-\Delta \Delta {C}_{{\rm{t}}}}$ is calculated as a range depending on the s.d. of the ΔΔ*C*_t_term (2^(*x*)^ is a strictly positive, growing function but nonlinear). The results were then normalized setting the mean vehicle value to 1.0 or 100% for each gene of interest.

### RNA-seq and GSEA

RNA was extracted as described above with the only change than an on-column DNA digest was performed according to the manufacturer’s instructions (RNase-free DNase Set, 79254). The RNA concentration and integrity was analysed using RNA screen Tape (5067–5576, 5067–5578, 5067–5577) on the TapeStation 2200 Device (Agilent) according to the manufacturer’s instructions. RNA-seq was performed using the TruSeq Stranded mRNA Library Prep kit (Illumina, 20020595) and either the HiSeq 4000 or the NovaSeq 6000 instruments from Illumina.

The Pisces pipeline^50^ (v.0.1.3.1) was used to quantify gene-level expression. All further analyses were performed using R (v.4.2.1) Statistical Software. Differential gene expression analysis between two conditions (treatment and control) was performed using DESeq2 (ref. ^51^) (v.1.36.0). The resulting *P* values were corrected for multiple testing using the Benjamini–Hochberg procedure. Gene set enrichment analysis (GSEA) was run to test for gene sets that were up- or downregulated in each cell line after *WRN* knockout. In particular, we used the R package fgsea^52^ (v.1.25.1) to estimate normalized enrichment statistics and associated *P* values, for each gene set in the Hallmark Collection (h.all.v6.2.symbols.gmt) from the Molecular Signatures Database^53^ with nperm = 10,000.

### Immunofluorescence

For nuclear γH2Ax staining, cells were seeded in 96-well plates (CellCarrier-96 Ultra Collagen-coated PerkinElmer, 6055700) with 10^5^ cells per 100 µl per well and incubated at 37 °C for 18 h. Treatment with HRO761 was performed using the HP Dispenser. After compound exposure, cells were fixed in 4% (v/v) paraformaldehyde (PFA) in PBS for 10 min at room temperature, washed twice with PBS and permeabilized/ blocked with 0.5% (v/v) Triton X-100, 0.5% BSA in PBS for 1 h at room temperature. Cells were then incubated with 1:2,000 anti-γH2AX (Millipore, 05-636) primary antibodies in blocking buffer overnight at 4 °C. The cells were then washed three times with 0.5% (v/v) Triton X-100 in PBS, followed by incubation with 1:2,000 goat anti mouse Alexa Fluor 488-conjugated antibodies (Invitrogen, A11001) and 1 µg ml^−1^ DAPI in blocking buffer for 1 h at room temperature. Cells were then washed three times with 0.5% (v/v) Triton X-100, and 200 μl PBS was finally added to each well before imaging. Images were captured and analysed using the PerkinElmer Opera Phenix imager and Harmony 4.9 software.

### Flow cytometry staining

About 2 × 10^6^ cells were collected by trypsin and washed once with PBS. Cells were pelleted and fixed in 100 µl 4% formaldehyde (Sigma-Aldrich, 47608) for 15 min at room temperature. Cells were washed by centrifugation with an excess of 1× PBS. Pellets were resuspended in 100 µl 1× PBS, then 900 µl of ice-cold methanol (Sigma-Aldrich, 34860) was added drop by drop while vortexing. Cells were incubated for 10 min on ice to permeabilize them. Cells were washed by centrifugation with excess of 1× PBS, pellets were resuspended in 100 µl anti-phosphorylated-histone H3 Ser10 conjugated to PE (Cell Signalling Technology, 5764) antibodies diluted 1:50 in 0.5% BSA PBS buffer and incubated for 1 h at room temperature protected from the light. After two washes by centrifugation with an excess of 1× PBS, cells were stained with 3 µM final concentration of DAPI solution (Thermo Fisher Scientific, 62248) for 1–5 min protected from the light. Cells were washed twice by centrifugation with an excess of 1× PBS. Stained cells were analysed on the Cytoflex flow cytometer (Beckman Coulter).

### Flow cytometry data analysis

Data were collected in 10^5^ particle units and analysed with CytExpert v.2.4 software. Cell debris and dead cells were excluded on the basis of forward scatter area (FSC-A) and side scatter area (SSC-A) profiles. Subsequently, singlets were identified based on FSC-A and forward scatter-height (FSC-H) profiles. These singlets were then analysed for DAPI (DNA content) and PE (phosphorylated histone H3 Ser10) staining intensities. Data were analysed to show the percentage of nuclei in the G1, S and G2/M phases using the DAPI channel and the percentage of cells specifically in G2 or M phase using the PE channel.

### Cell synchronization with double thymidine block

Cells were blocked for 18 h with 2 mM thymidine solution (Sigma-Aldrich, T1895). Cells were then released by washing with PBS followed by addition of complete medium and incubation for 9 h. Then, 2 mM thymidine solution was added again for 15 h. Cells were next collected (*t* = 0 h), then washed with PBS and complete medium added. Cells were collected every 2 h until 12 h. For the M phase control, unsynchronized cells were treated with 330 nM of nocodazole (Sigma-Aldrich, M1404) for 18 h.

### Cell fractionation

A total of 6 × 10^6^ cells was plated and treated the day after as indicated. Cells were processed according to the manufacturer’s instructions with the FOXP3/Transcription Factor Staining Buffer kit (Thermo Fisher Scientific, 00-5523-00) and the samples run by western blot as described above.

### In vivo experiments

All animal studies were conducted in accordance with ethics and procedures covered by permit BS-2275 and BS-1975 issued by the Kantonales Veterinäramt Basel-Stadt and in strict adherence to guidelines of the Eidgenössisches Tierschutzgesetz and the Eidgenössische Tierschutzverordnung, Switzerland. All animal studies were approved by the internal ethics committee. All animals had access to food and water ad libitum and were identified with transponders. They were housed in a specific-pathogen-free facility in IVC racks under a 12 h–12 h light–dark cycle. To establish cell-line-derived xenograft models, 6–7-week-old female athymic nude (Crl:NU(NCr)-*Foxn1*^nu^) or SCID-BEIGE (CB17.Cg-PrkdcscidLystbg-J/Crl) mice from Charles River were engrafted subcutaneously with 5 million tumour cells in HBSS (H6648, Sigma-Aldrich). For SNU-407, Lovo, IM95, RL95.2, Ishikawa and IGROV-1, the cells were concentrated in 50% Matrigel (354234, Corning). Patient-derived xenografts were induced and expanded by transplantation as previously described^54^. The amorphous sodium salt of HRO761 was dissolved in 20% in hydroxypropyl-beta-cyclodextrin in water. For the efficacy experiment, mice were randomized into groups (*n* = 5–6) for a mean tumour size of 100–150 mm^3^. For the SW48 model, HRO761 was dosed orally once daily at multiple doses up to 120 mg per kg until relapse. Blood samples were collected on day 7 and 91. For the other CDX and PDX models, HRO761 was dosed once daily at 60 or 120 mg per kg for 3–4 weeks. Tumour responses are reported with the measures of tumour volumes from the treatment start. For the PD experiment in SW48 tumour-bearing mice, HRO761 was given by oral gavage daily at 20 or 60 mg per kg. Animals were randomized (*n* = 3) and tumour samples were collected at 0, 1, 4, 8 and 24 h on the first day and then collected 4 h after the last treatment on day 3, 8, 10, 14 (including a 24 h timepoint), 17 and 21. Tumours were excised, weighed, either frozen in liquid nitrogen and cryogenic dry pulverized with the CryoPrep system (model CP-02, Covaris) or processed to FFPE after fixation. For efficacy experiments with compound **3** in the SW48 model, mice were randomized into groups (*n* = 7) for a mean tumour size of ~200 mm^3^ and dosed twice daily at 15, 50 and 150 mg per kg for 13 days. On day 12, blood samples were collected for PK analysis. On the last day (3 h after the last treatment), tumours were excised, weighed, frozen in liquid nitrogen and cryogenic dry pulverized for PD analysis. For combination experiments in the SW48 model, mice were randomized into groups (*n* = 5) for a mean tumour size of about 170 mm^3^ and treated with either HRO761 orally once daily at 20 mg per kg, or irinotecan intravenously once weekly at 60 mg per kg, or a combination of HRO761 with a decreasing dose of irinotecan from 60 to 15 mg per kg.

### Bioanalytics and PK parameters

Blood samples were extracted with acetonitrile and HRO761 or compound **3** concentrations were determined using UPLC–MS/MS using internal and authentic standards in blank blood. PK parameters were determined by non-compartmental analysis. Intravenous bolus studies in mouse, rat and dog were performed at 1, 1 and 0.1 mg per kg respectively (Extended Data Table 1).

### Immunohistochemistry

Formalin-fixed paraffin-embedded sections were stained on a Leica Bond RX using the Opal 7 colour Automation IHC detection kit (Akoya Biosciences, NEL871001KT) and Opal Polymer anti-rabbit HRP kit (Akoya Biosciences, ARR1001KT) according to the manufacturer’s instructions with epitope retrieval 1 (ER1) conditions for 20 min at 100 °C. For immunohistochemical staining, WRN and pCHK2 were evaluated using the antibodies described in Supplementary Table 4. Nuclear size evaluation in tumour cells was performed using the HALO (Indica Labs) Cytonuclear IHC algorithm that measures cell by cell immunohistochemistry positivity for single-stain application. For immunofluorescence analysis, DDR response to treatment (pCHK2, p21 and γH2AX), tumour content (pan-CK) and tumour cell proliferation (Ki-67) were evaluated using the antibodies coupled to specific Opal dyes in the described order listed in Supplementary Table 5. Slides were mounted with Aqueous Mounting Medium proLong gold antifade reagent (Invitrogen, P36930) and allowed to dry before being scanned at ×20 magnification on the PhenoImager HT multispectral imaging system (Akoya Biosciences).

### Statistics and reproducibility

All immunoblots were repeated three times, except for blots shown in Figs. 2e and 4c and Extended Data Figs. 6b and 8f, which were performed twice. Representative results from one experiment are shown.

For Fig. 4d, pictures are representative of one study and animal with three animals per group and timepoint in SW48, but the same observations were made on different colorectal cancer MSI^high^ xenograft and PDX models.

For Extended Data Fig. 1f, pictures are representative of one study and animal with three animals per group, condition and timepoint.

### Reporting summary

Further information on research design is available in the Nature Portfolio Reporting Summary linked to this article.

## Online content

Any methods, additional references, Nature Portfolio reporting summaries, source data, extended data, supplementary information, acknowledgements, peer review information; details of author contributions and competing interests; and statements of data and code availability are available at 10.1038/s41586-024-07350-y.

### Supplementary information

Supplementary Information

Reporting Summary

Peer Review File

### Source data

Source Data Fig. 4

Source Data Extended Data Fig. 1

Source Data Extended Data Fig. 4

Source Data Extended Data Fig. 7

## Figures and Tables

**Extended Data Table 1:**
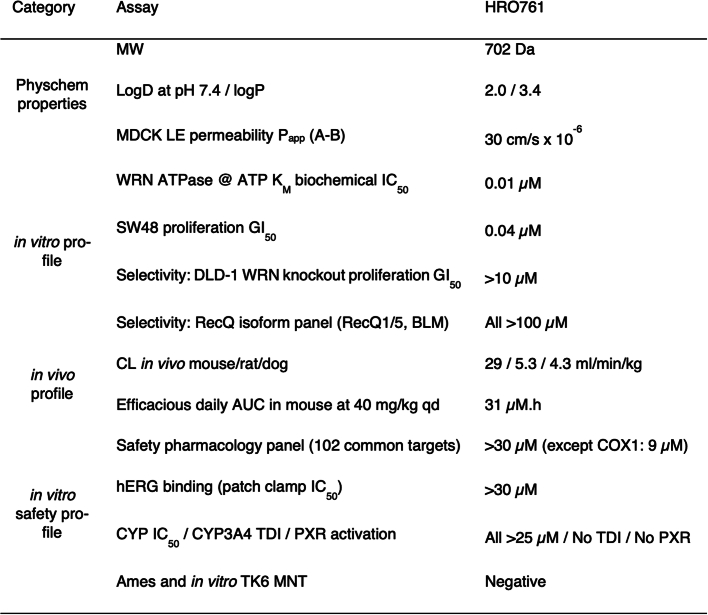
Key properties of clinical candidate HRO761 (4)

**Extended Data Table 2:**
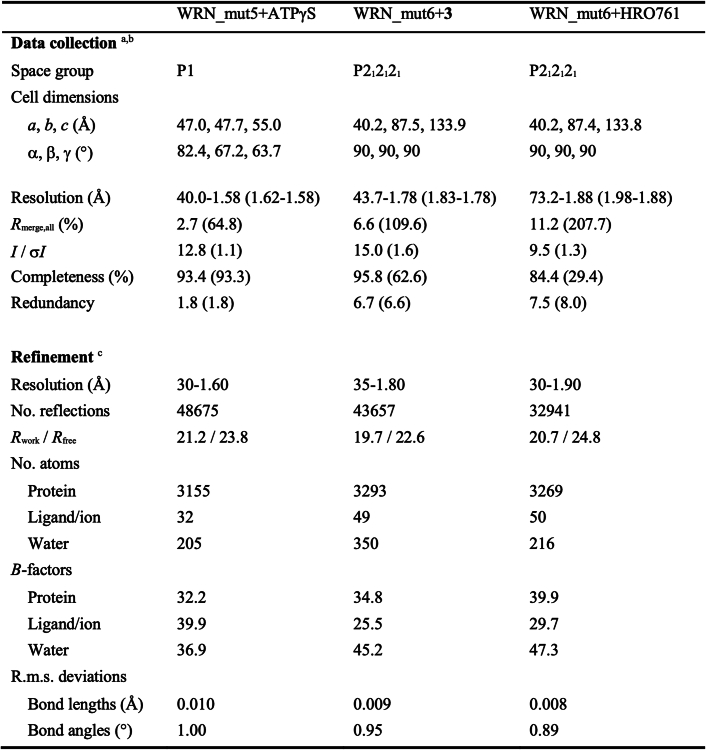
Crystallographic data collection and refinement statistics

^a^ Values as reported in XDS/XSCALE^36^ for the ATPγS complex and autoPROC/STARANISO^37,38^ for the complexes with **3** and HRO761. ^b^ Highest resolution shell is shown in parentheses. ^c^ Values as defined in BUSTER 2.11.8^42^.

## Data Availability

Atomic coordinates and structure factors for the co-crystal structures have been deposited in the PDB under accession codes 8PFP (WRN–ATPγS), 8PFL (WRN–**3**) and 8PFO (WRN–HRO761). The synthesis of compounds **2**–**6**, including ^1^H and ^13^C NMR data, is described in the Supplementary Information. All raw sequencing data from this study have been deposited at the SRA under BioProject IDs PRJNA995921 and PRJNA995923. The MSS proteomics data have been deposited to the ProteomeXchange Consortium through the PRIDE 56 partner repository under dataset identifier PXD044202. Source data are provided with this paper.
