# Supplementary material for: Discovery of WRN inhibitor HRO761 with synthetic lethality in MSI cancers
Source: Nature. 2024 Apr 24;629(8011):443–9. doi: 10.1038/s41586-024-07350-y (PMC11078746; doi:10.1038/s41586-024-07350-y)
Supplement: Supplementary file 1 — Supplementary Information [file 41586_2024_7350_MOESM1_ESM.pdf]

nature portfolio

https://doi.org/10.1038/s41586-024-07350-y

Supplementary information

Discovery of WRN inhibitor HRO761 with synthetic lethality in MSI cancers

In the format provided by the authors and unedited

Supplementary Information

SI Guide

Discovery of allosteric WRN inhibitor HRO761 demonstrating synthetic lethality in MSI cancers

Stephane Ferretti[1#], Jacques Hamon[1#], Ruben de Kanter[1#], Clemens Scheufler[1#], Rita Andraos-Rey[1], Stephanie Barbe[1], Elisabeth Bechter[1], Jutta Blank[1], Vincent Bordas[1], Ernesta Dammassa[1], Andrea Decker[1], Noemi Di Nanni[1], Marion Dourdoigne[1], Elena Gavioli[4], Marc Hattenberger[1], Alisa Heuser[1], Christelle Hemmerlin[1], Jürgen Hinrichs[1], Grainne Kerr[1], Laurent Laborde[1], Isabel Jaco[1], Eloísa Jiménez Núñez[1,3], Hans-Joerg Martus[1], Cornelia Quadt[1], Markus Reschke[1], Vincent Romanet[1], Fanny Schaeffer[1], Joseph Schoepfer[1], Maxime Schrapp[1], Ross Strang[1], Hans Voshol[1], Markus Wartmann[1], Sarah Welly[1], Frédéric Zecri[2], Francesco Hofmann[1,3], Henrik Möbitz[1✉], Marta Cortés-Cros[1✉]

# These authors contributed equally: Stephane Ferretti, Jacques Hamon, Ruben de Kanter and Clemens Scheufler

✉ Corresponding authors: marta.cortes-cros@novartis.com, henrik.moebitz@novartis.com

Authors Affiliations

[1]Novartis BioMedical Research, CH-4056 Basel, Switzerland
[2]Novartis BioMedical Research, Cambridge, MA 02139, USA
[3]Present address: Pierre Fabre Laboratories, 31000 Toulouse, Franc
[4]Novartis Pharma AG, Basel, Switzerland

Table of contents:

SI Figure 1 | Uncropped immunoblots from Main Figures……………………Pages 2-3

SI Figure 2 | Uncropped immunoblots from Extended Data Figures 1-8……..Pages 4-9

SI Figure 3 | Gating strategies for FACS analysis…………………………...…Page 10

Supplementary Information to Extended Data Figure 1…………..……………Page 11

Supplementary Methods:
Synthesis of compounds **2-6,** including ^{1}H and ^{13}C NMR data…...………**Pages 12-29**

SI Table 1 | Proliferation inhibition of HRO761 for a panel of cell lines……..Page 30

SI Table 2 | Antibodies used for WRN degradation ELISA……………………..Page 31

SI Table 3 | TaqMan probes used for RTqPCR……………….………………….Page 31

SI Table 4 | Antibodies used for immunohistochemical stainin..……………..Page 31

SI Table 5 | Antibodies coupled with specific Opal dyes used for tumor content (PanCK) and tumor cells proliferation (Ki67) staining……….Page 31

SI Table 6 | Cell lines name, growth media used and cell line origin……...…Page 32

Supplementary Figure 1: Uncropped immunoblots from Main Figures.

For Figure 2

Used in Figure 2d

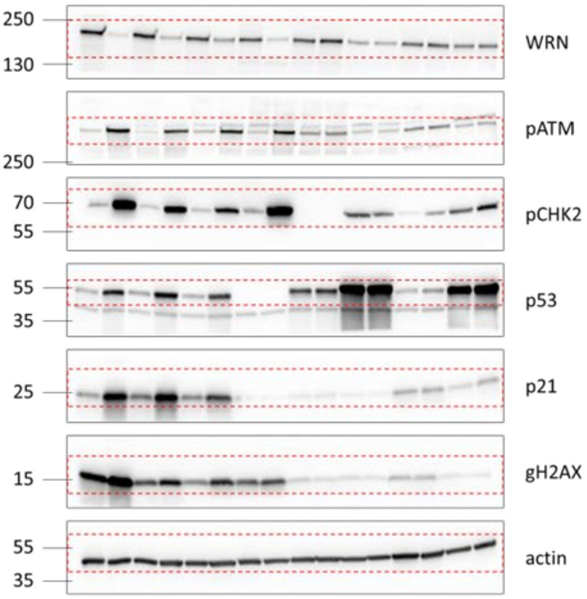

Used in Figure 2e

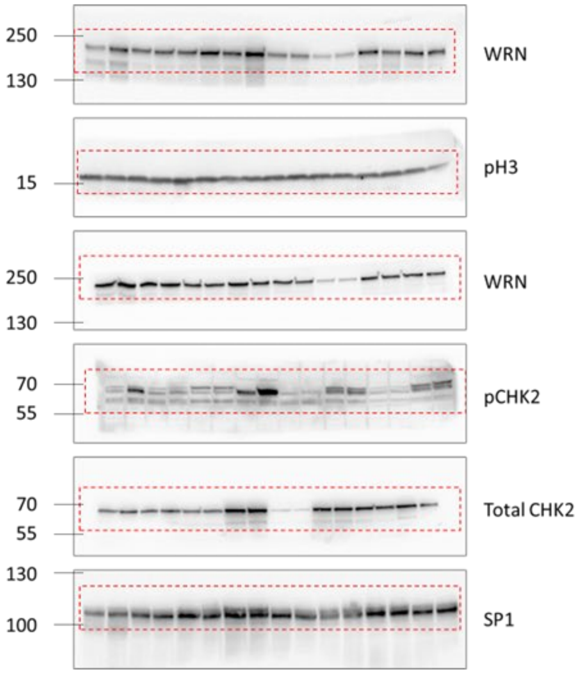

For Figure 3

Used in Figure 3a

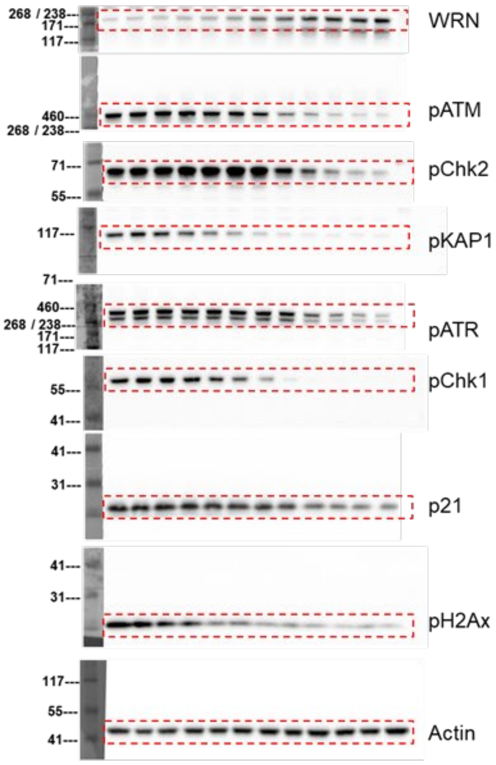

Used in Figure 3g

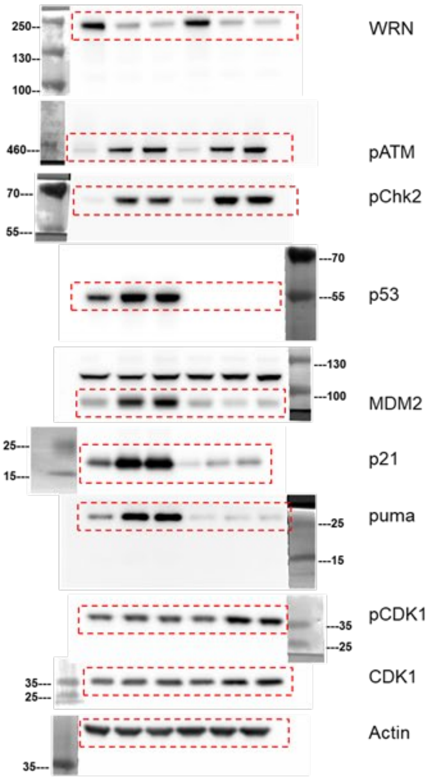

Supplementary Figure 1: **Uncropped immunoblots from Main Figures.**

For Figure 4

Used in Figure 4c left

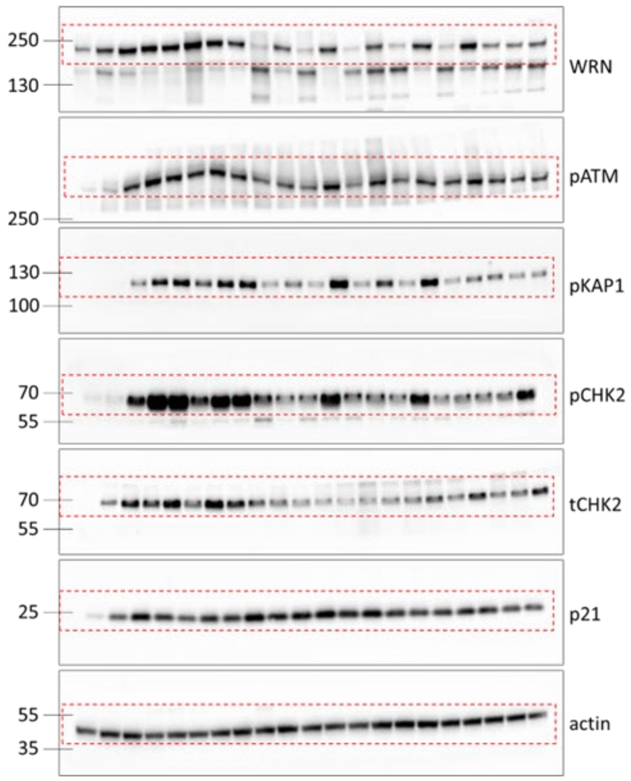

Used in Figure 4c right

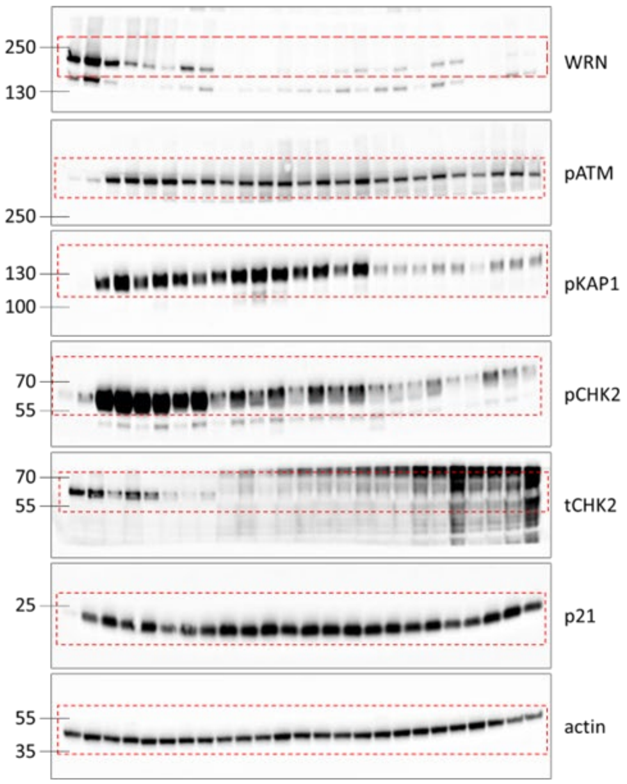

Supplementary Figure 2: Uncropped immunoblots from Extended Data Figures 1-8

For ED Figure 1

Used in ED Figure 1d

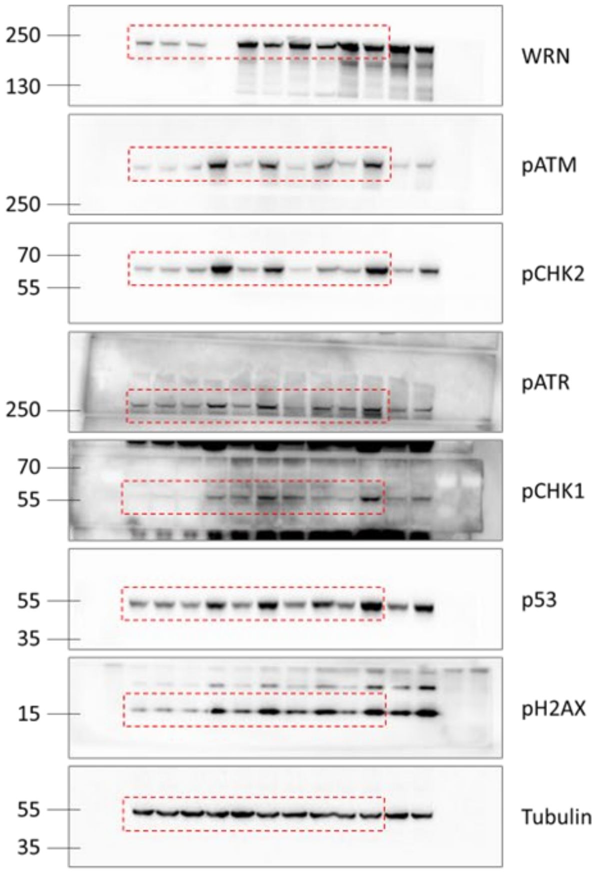

Used in ED Figure 1h

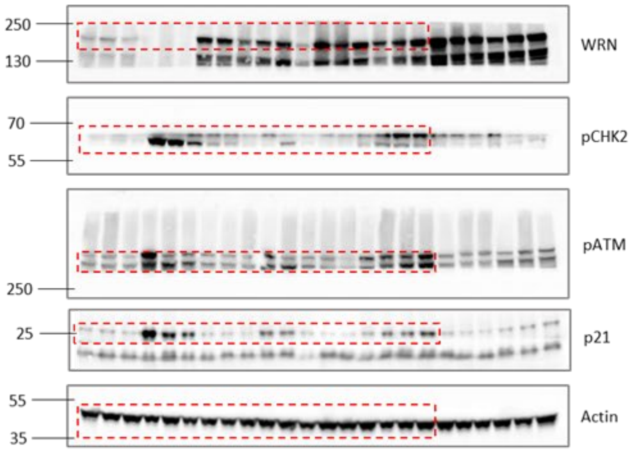

Used in ED Figure 1i

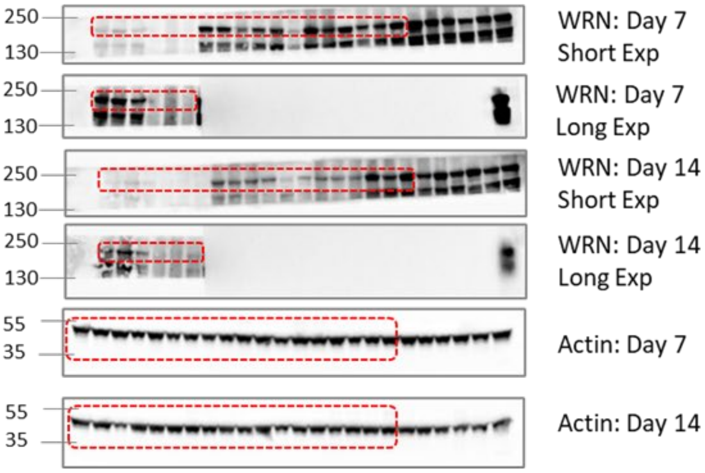

Supplementary Figure 2: Uncropped immunoblots from Extended Data Figures 1-8

For ED Figure 3

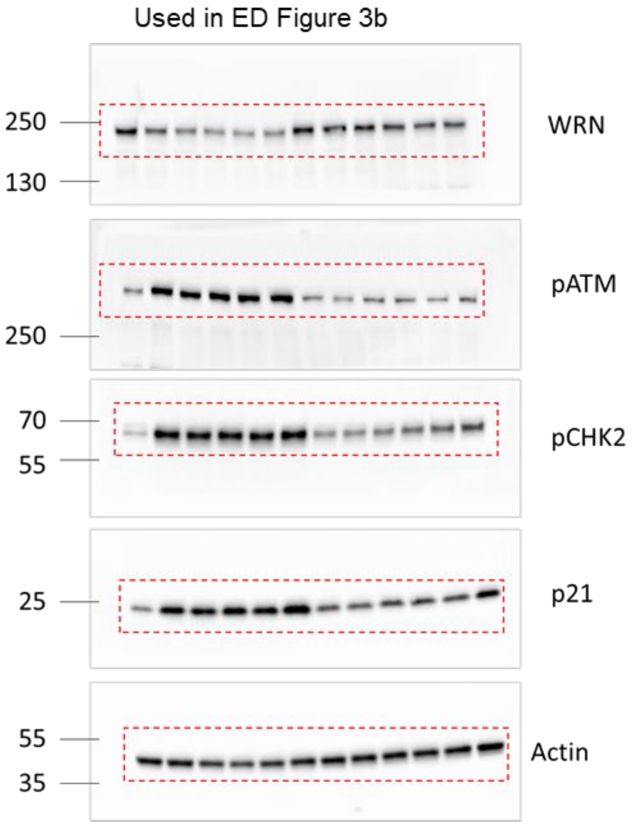

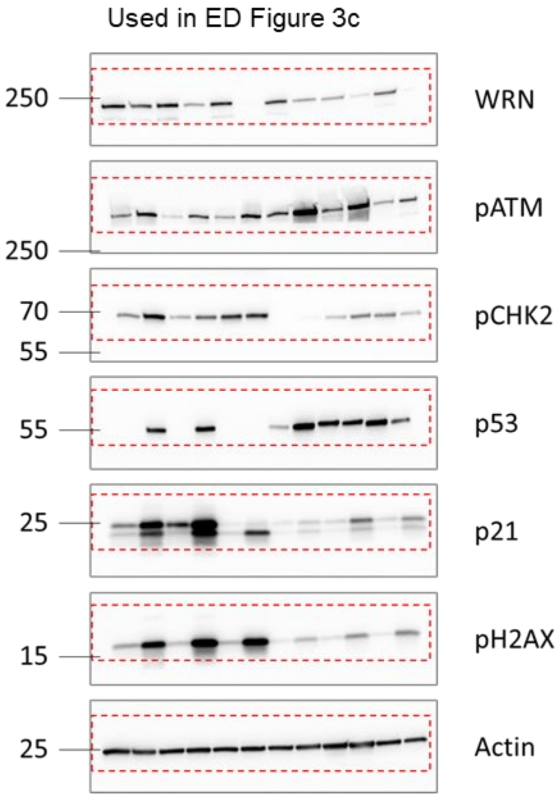

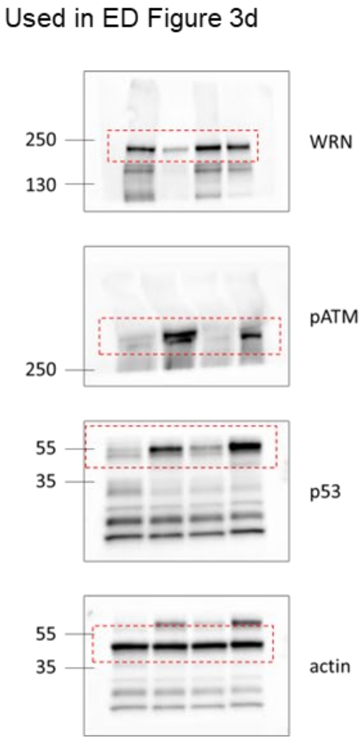

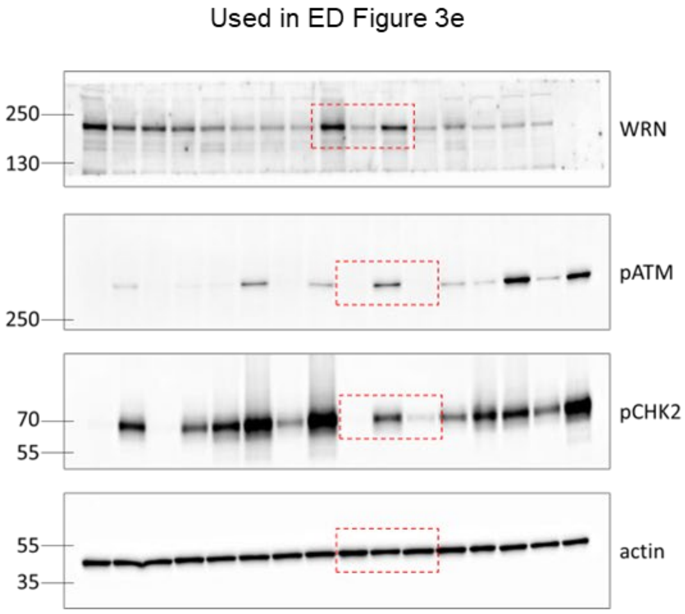

Supplementary Figure 2: Uncropped immunoblots from Extended Data Figures 1-8

For ED Figure 3

Used in ED Figure 3h

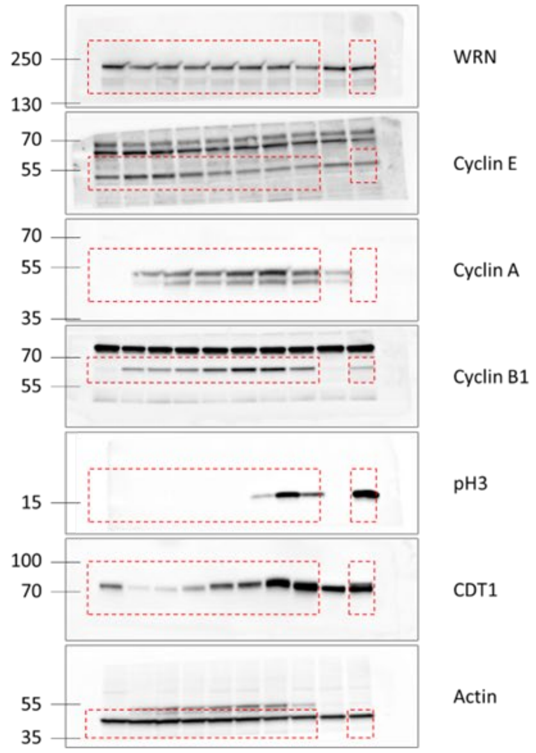

Used in ED Figure 3i

Etoposide

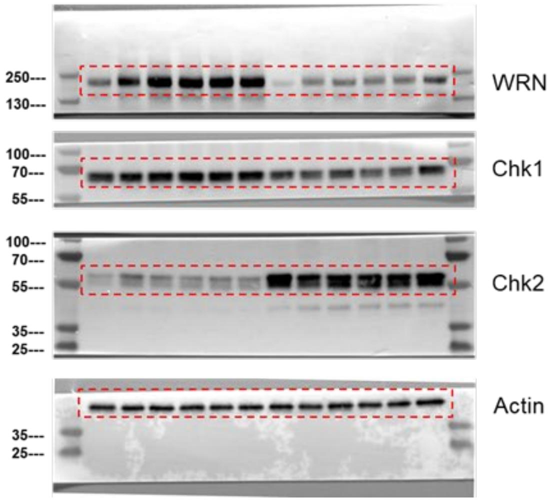

Camptothecin

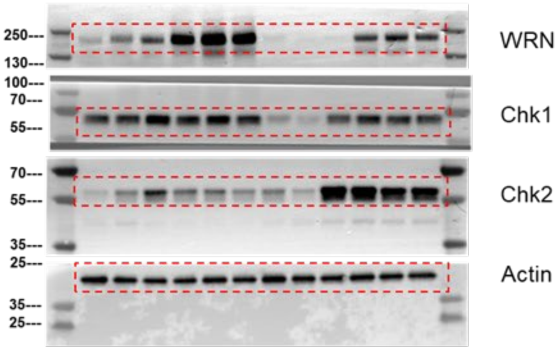

Supplementary Figure 2: Uncropped immunoblots from Extended Data Figures 1-8

For ED Figure 5

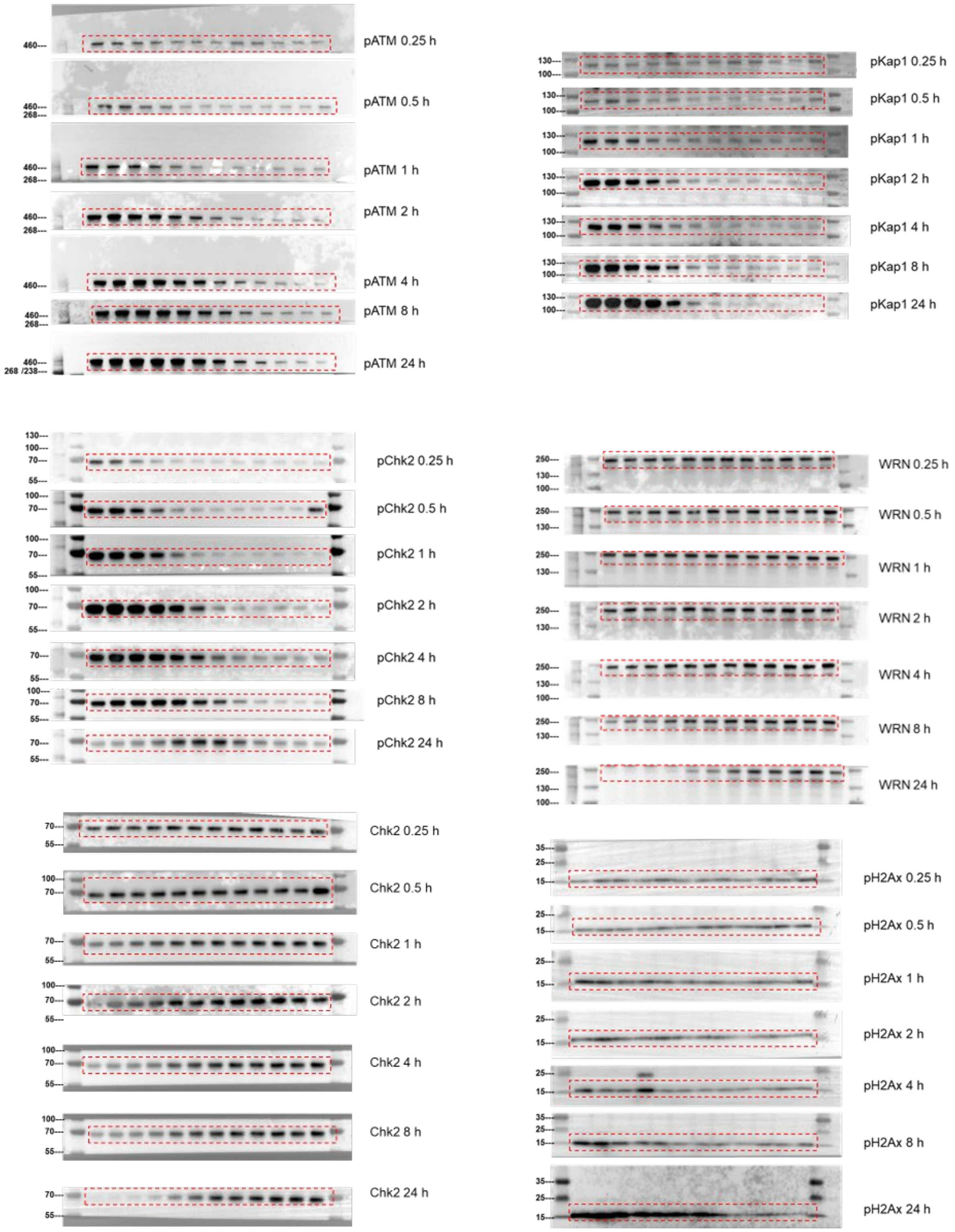

Supplementary Figure 2: Uncropped immunoblots from Extended Data Figures 1-8

For ED Figure 5

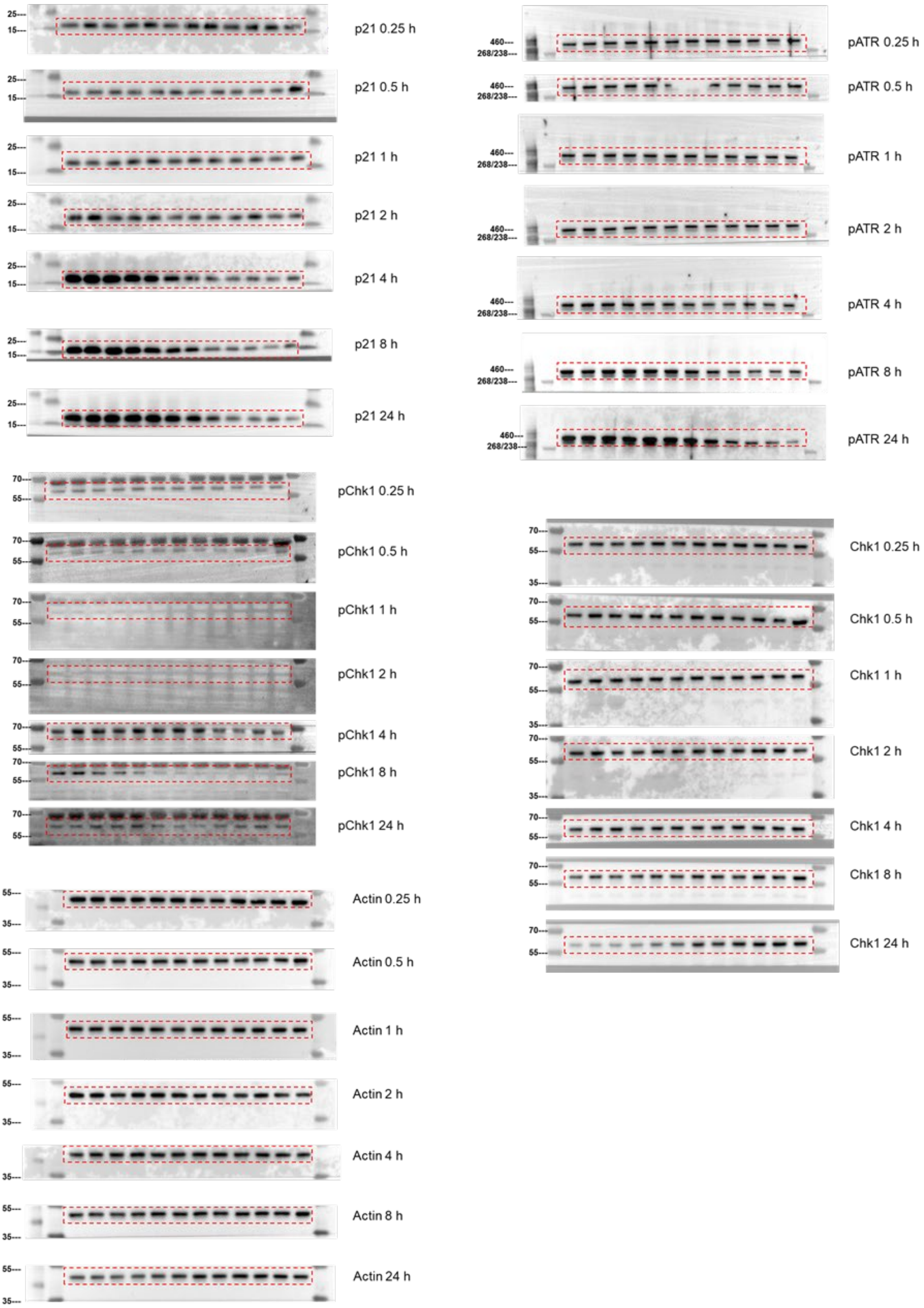

Supplementary Figure 2: Uncropped immunoblots from Extended Data Figures 1-8

Used in ED Figure 6a

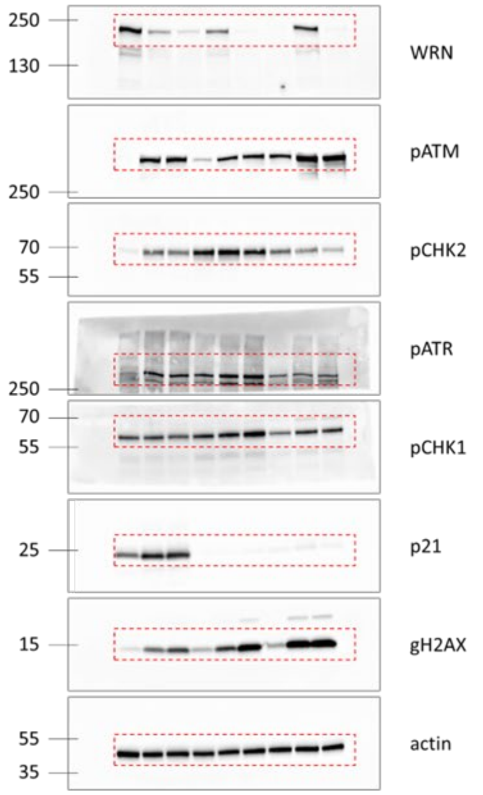

Used in ED Figure 6b

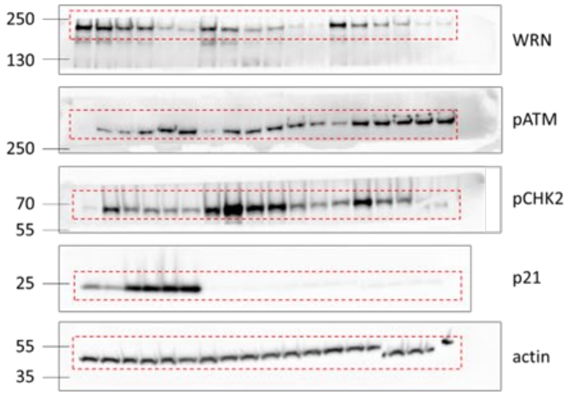

Used in ED Figure 7c

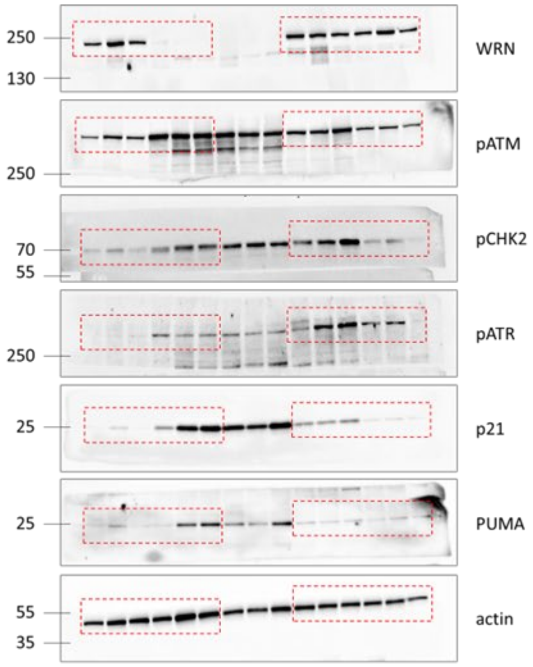

Used in ED Figure 8b

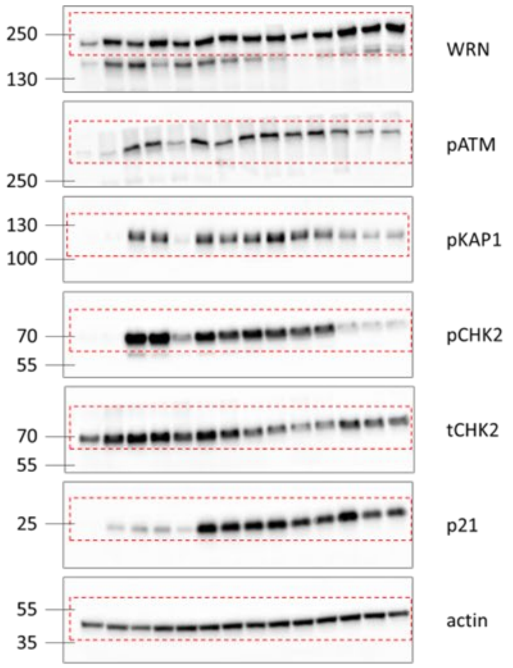

Used in ED Figure 8g

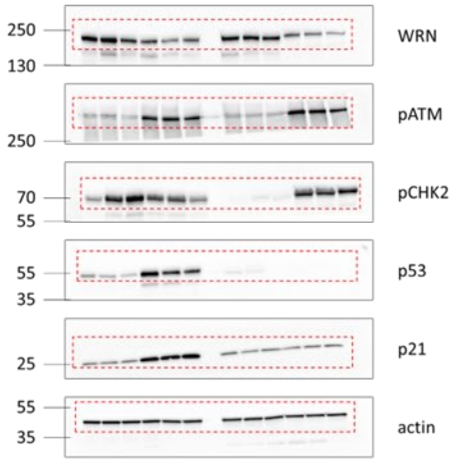

Supplementary Figure 3

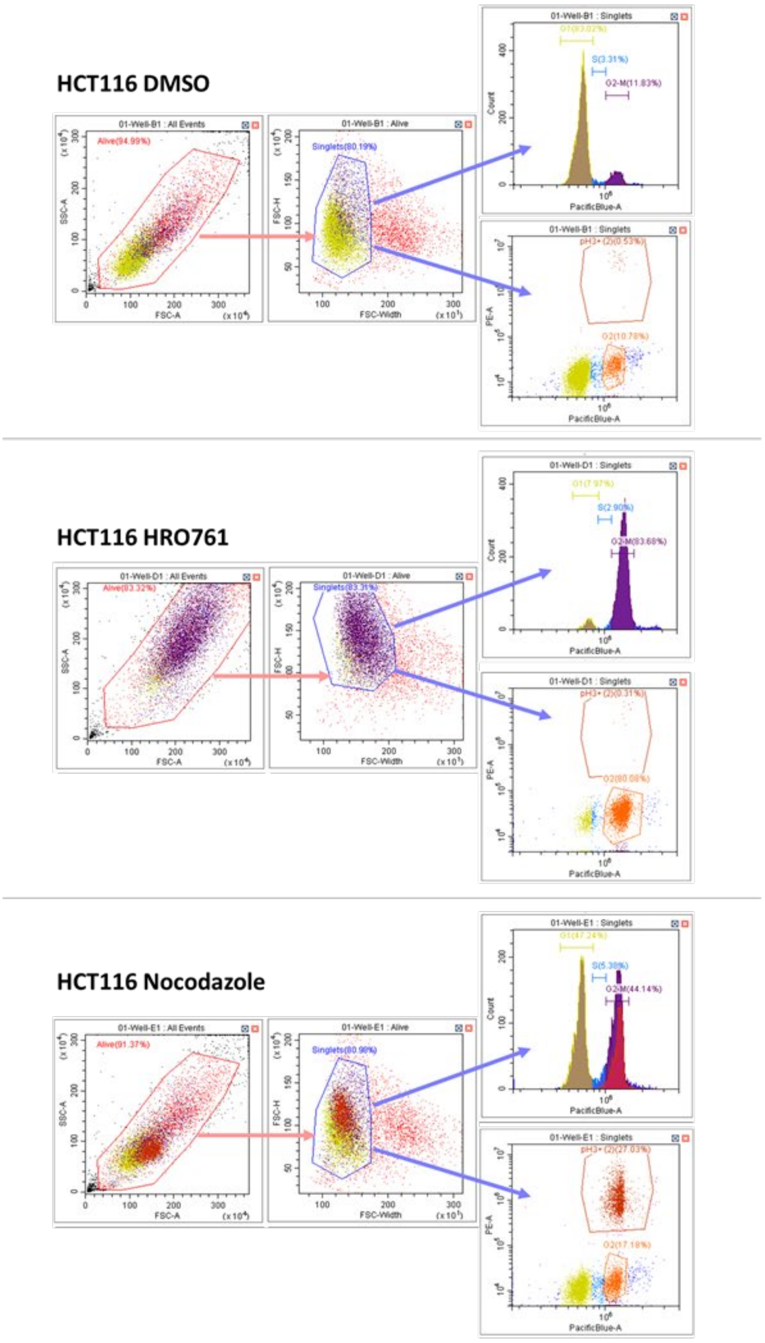

SI Figure 3 | Gating strategies for FACS analysis. Gating strategy for Fig. 3d. Cell debris and dead cells were excluded based on forward scatter-area (FSC-A) and side scatter-area (SSC-A) profiles. Subsequently, singlets were identified based on FSC-A and forward scatter-height (FSC-H) profiles. These singlets were then analysed for DAPI (DNA content) and PE (phospho-Histone 3 Ser10) staining intensities.

Supplementary Information to Extended Data Figure 1.

Genetic validation studies with WRN shRNA knockdown and WRN WT and mutant rescue constructs

Genetic dependence on WRN was confirmed with an inducible shRNA to WRN (sh19-WRN), which showed loss of viability of RKO cells upon induction of the shRNA and knockdown of the WRN protein (Extended Data Fig. 1c and d). The wild type WRN protein as well as two different loss-of-function mutations within the helicase domain (K577A) and exonuclease domain (E84A) of the WRN protein (Extended Data Fig. 1b) were introduced to demonstrate, firstly, that the viability effects were due to an on-target effect of WRN knockdown both *in vitro* and *in vivo* (Extended Data Fig. 1c, e, f and g) and, secondly, that the responsible enzymatic activity for the WRN dependence was the helicase activity as expression of the WRN WT and E84A cDNAs rescued the DNA damage and cell proliferation phenotype but the K577A WRN cDNA did not (Extended Data Fig. 1c-e, h-i).

While *in vivo* there was an initial impairment of proliferation with the K577A helicase mutant, tumors grew over time. We observed that there was an increase in WRN levels between day 7 and day 14 in the tumors with the WRN K577A mutant cDNA (Extended Data Figure 1i). Since the immunoblot could not differentiate between endogenous WRN and exogenous, K577A mutant WRN, we measured the transcript levels of both in tumors, and found that it was the K577A WRN mutant that was selectively upregulated in the tumors after 2 weeks of dox induction removing WRN WT (Extended Data Figure 1j). We therefore measured the enzymatic activity from both WT and K577A helicase domain proteins in an ATPase assay and found that the K577A WRN mutant is not completely dead but retains ~1-2 % of activity (Extended Data Figure 1k). This explains why these tumors upregulate the expression of the K577A WRN protein, to have enough helicase activity for their survival. This points to the strong WRN dependence of this cell line and shows a first genetic mechanism of “resistance” which upregulates the mutant K577A WRN in order to compensate for the loss of endogenous WRN as an escape mechanism.

Supplementary Methods: Synthesis of compounds **2-6,** including ^{1}H and ^{13}C NMR data

Instrumentation

UPLC-MS 1:

Instrument	Waters Acquity UPLC with Waters SQ detector
Column	CORTECS™ C18+ 2.7μm,
Column Dimension	2.1 x 50 mm
Column Temperature	80°C
Eluents	A: water + 4.76% isopropanol + 0.05 % FA + 3.75 mM AA
	B: isopropanol + 0.05 % FA
Flow Rate	1.0 mL/min
Gradient	1 to 50% B in 1.4 min; 50 to 98% B in 0.3 min

UPLC-MS 2:

Instrument	Waters Acquity UPLC with Waters SQ detector
Column	ACQUITY UPLC® BEH C18 1.7 μm
Column Dimension	2.1 x 100 mm
Column Temperature	80°C
Eluents	A: water + 4.76% isopropanol + 0.05% FA + 3.75 mM AA
	B: isopropanol + 0.05% FA
Flow Rate	0.4 mL/min
Gradient	1 to 60% B in 8.4 min; 60 to 98% B in 1.0 min

UPLC-MS 3:

Instrument	Waters Acquity UPLC with Waters SQ detector
Column	ACQUITY UPLC® BEH C18 1.7 μm
Column Dimension	2.1 x 50 mm
Column Temperature	80°C
Eluents	A: water + 4.76% isopropanol + 0.05% FA + 3.75 mM AA
	B: isopropanol + 0.05% FA
Flow Rate	0.6 mL/min
Gradient	1 to 98% B in 1.7 min

UPLC-MS 4:

Instrument	Waters Acquity UPLC with Waters SQ detector

Column	ACQUITY UPLC® BEH C18 1.7 µm
Column Dimension	2.1 x 50 mm
Column Temperature	80°C
Eluents	A: water + 0.05% FA + 3.75 mM AA
	B: isopropanol + 0.05% FA
Flow Rate	0.6 / 0.7 mL/min
Gradient	5 to 98% B in 1.7 min

<u>UPLC-MS 7:</u>

Instrument	Waters Acquity UPLC with Waters SQ detector
Column	Acquity UPLC® HSS T3 1.8 µm
Column Dimension	2.1 x 50 mm
Column Temperature	60°C
Eluents	A: water + 0.05% formic acid + 3.75 mM ammonium acetate
	B: acetonitrile + 0.04% FA
Flow Rate	1.0 mL/min
Gradient	2 to 98% B in 1.4 min

<u>UPLC-MS 8:</u>

Instrument	Waters Acquity UPLC with Waters SQ detector
Column	Acquity UPLC® HSS T3 1.8 µm
Column Dimension	2.1 x 50 mm
Column Temperature	60°C
Eluents	A: water + 0.05% formic acid + 3.75 mM ammonium acetate
	B: acetonitrile + 0.04% FA
Flow Rate	1.0 mL/min
Gradient	5 to 98% B in 1.4 min

<u>HPLC 4:</u>

Instrument	Agilent 1260
Column	Agilent Poroshell 120 EC-C18, 2.7 µm
Column Dimension	4.6 x 50 mm
Column Temperature	40°C
Eluents	A: water + 0.1% TFA
	B: acetonitrile + 0.1% TFA
Flow Rate	1.2 mL/min
Gradient	5% B to 95% B in 5 min, hold 2 min

HPLC 6:

Instrument	Agilent 1260 infinity series HPLC system with DAD/ELSD
Column	Atlantis dC18, 5 µm
Column Dimension	4.6 x 250 mm
Column Temperature	25°C
Eluents	A: water + 0.1% TFA
	B: acetonitrile
Flow Rate	1.0 mL/min
Gradient	10% B to 100% B in 15 min, hold 5 min

RP-HPLC acidic 1:

System	Gilson
Column	Waters SunFire Prep C18 OBD (100 mm x 30 mm), 5 µm
Eluents	A: water + 0.1% TFA, B: acetonitrile
Flow rate	40 mL/min

RP-HPLC acidic 2:

System	Gilson
Column	Waters SunFire C18 OBD (250 mm x 4.6 mm), 5 µm
Eluents	A: water + 0.05% TFA, B: acetonitrile + 0.05% TFA
Flow rate	1 mL/min
UV detection	254 nm
Radio detector	Berthold LB 513
Cocktail	Zinsser Quickszint Flow 302

N-(2-chloro-4-(trifluoromethyl)phenyl)-2-(2-(3,6-dihydro-2H-pyran-4-yl)-5-ethyl-6-(4-(5-hydroxy-6-methylpyrimidine-4-carbonyl)piperazin-1-yl)-7-oxo-[1,2,4]triazolo[1,5-a]pyrimidin-4(7H)-yl)acetamide, **4** (HRO761)

Step 1: 2-(6-(4-(5-(benzyloxy)-6-methylpyrimidine-4-carbonyl)piperazin-1-yl)-2-(3,6-dihydro-2H-pyran-4-yl)-5-ethyl-7-oxo-[1,2,4]triazolo[1,5-a]pyrimidin-4(7H)-yl)-N-(2-chloro-4-(trifluoromethyl)phenyl)acetamide

To a stirred solution of N-(2-chloro-4-(trifluoromethyl)phenyl)-2-(2-(3,6-dihydro-2H-pyran-4-yl)-5-ethyl-7-oxo-6-(piperazin-1-yl)-[1,2,4]triazolo[1,5-a]pyrimidin-4(7H)-yl)acetamide (Intermediate AK) (300 mg, 429 µmol), 5-(benzyloxy)-6-methylpyrimidine-4-carboxylic acid (Intermediate CY) (115 mg, 472 µmol) and HATU (245 mg, 644 µmol) in DMF (3 mL) was

added DIPEA (375 µL, 2.15 mmol) at RT and the RM was stirred at RT for 15 minutes. The RM was diluted with EtOAc/water, extracted twice with EtOAc and the combined organic extracts were dried over Na_2SO_4 and concentrated. The crude product was purified by column chromatography (RediSep column: silica 24 g, eluent DCM:MeOH 100:0 to 90:10). The product containing fractions were combined and concentrated to give the title compound as a beige foam. The product was dissolved in EtOH, stirred at 60 °C over 18 hours, then cooled down to 0 °C, filtered off and washed with EtOH to give the title compound as a white solid (316 mg, purity 93%, yield 86%). LC-MS: Rt = 1.20 min; MS m/z $[M+H]^+$ 792.4/794.4, m/z $[M-H]^-$ 790.6/792.6; UPLC-MS 1

Step 2: N-(2-chloro-4-(trifluoromethyl)phenyl)-2-(2-(3,6-dihydro-2H-pyran-4-yl)-5-ethyl-6-(4-(5-hydroxy-6-methylpyrimidine-4-carbonyl)piperazin-1-yl)-7-oxo-[1,2,4]triazolo[1,5-a]pyrimidin-4(7H)-yl)acetamide

To a stirred suspension of 2-(6-(4-(5-(benzyloxy)-6-methylpyrimidine-4-carbonyl)piperazin-1-yl)-2-(3,6-dihydro-2H-pyran-4-yl)-5-ethyl-7-oxo-[1,2,4]triazolo[1,5-a]pyrimidin-4(7H)-yl)-N-(2-chloro-4-(trifluoromethyl)phenyl)acetamide (266 mg, 312 µmol) in DCM (6 mL) was added boron trichloride methyl sulfide complex (312 µL, 625 µmol) at RT and the RM was stirred at RT for 14 hours. The RM was quenched with MeOH. Then it was diluted with DCM/water, extracted twice with DCM and the combined organic extracts were washed with water and brine, dried over Na_2SO_4 and concentrated. The crude product was purified by column chromatography (RediSep column: silica 24 g, eluent DCM:MeOH 100:0 to 93:07). The product containing fractions were combined and concentrated to give **4** as an off-white solid. The product was dissolved in EtOH, stirred at 60 °C over 18 h, then cooled down to 0 °C, filtered off and washed with EtOH to give **4** as a white solid (216 mg, purity 99%, yield 99%).

LC-MS: Rt = 1.05 min; MS m/z $[M+H]^+$ 702.4/704.4, m/z $[M-H]^-$ 700.5/702.5; UPLC-MS 1; ^{1}H NMR (400 MHz, DMSO-d_6) δ 10.36 (s, br, 2H), 8.55 (s, 1H), 8.05 (d, J = 8.5 Hz, 1H), 7.96 (m, 1H), 7.71 (dd, J = 2.1 Hz, 8.8 Hz, 1H), 6.83 (m, 1H), 5.32 (s, 2H), 4.52 (m, 1H), 4.25 (m, 2H), 3.80 (m, 2H), 3.48 (m, 3H), 3.25 (m, 1H), 2.99 (m, 3H), 2.81 (m, 1H), 2.64 (m, 1H), 2.52 (m, 2H), 2.43 (s, 3H), 1.18 (t, J = 7.4 Hz, 3H); ^{13}C-NMR (125 MHz, DMSO-d_6): δ 166.0, 164.3, 161.0, 156.8, 156.7, 154.1, 151.3, 148.9, 146.8, 146.3, 138.1, 130.2, 126.8 (q, J_{CF} = 3.7 Hz), 126.3 (q, J_{CF} = 32.9 Hz), 125.8, 125.6, 125.3, 124.7 (q, J_{CF} = 3.7 Hz), 123.3 (q, J_{CF} = 272.3 Hz), 121.4, 64.7, 63.1, 50.3, 50.2, 49.8, 46.9, 41.9, 24.6, 21.0, 19.2, 12.8; HRMS (m/z): $[M+H]^+$ calcd for $C_{31}H_{32}O_5N_9ClF_3$, 702.21615; found, 702.21639.

2-(6-(4-(3,6-difluoro-2-hydroxybenzoyl)piperazin-1-yl)-2-(3,6-dihydro-2*H*-pyran-4-yl)-5-methyl-7-oxo-[1,2,4]triazolo[1,5-*a*]pyrimidin-4(7*H*)-yl)-*N*-(4-(trifluoromethyl)phenyl)acetamide, **2**

2-(2-(3,6-Dihydro-2H-pyran-4-yl)-5-methyl-7-oxo-6-(piperazin-1-yl)-[1,2,4]triazolo[1,5-a]pyrimidin-4(7H)-yl)-N-(4-(trifluoromethyl)phenyl)acetamide (Intermediate BN) (67.0 mg, 129 µmol), 3,6-difluoro-2-hydroxybenzoic acid (22.5 mg, 129 µmol) and TEA (18.0 µL, 129 mol) were combined in DMF (Volume: 1 mL). HATU (49.2 mg, 129 µmol) was added in one portion and the resulting mixture stirred at RT for 1 hour. The crude product was purified by reverse phase preparative HPLC (RP-HPLC acidic 1: 30 to 57% B in 20 min, 25 mL/min). Fractions containing desired product were lyophilized to give **2** as a colorless powder (19.0 mg, purity 97%, yield 21%).

LC-MS: Rt = 1.03 min; MS m/z $[M+H]^+$ 674.3, m/z $[M-H]^-$ 672.3; UPLC-MS 8; ^1H NMR (400 MHz, DMSO-d_6) δ 10.87 (br s, 2H), 7.78 (d, J = 8.6 Hz, 2H), 7.70 (d, J = 8.7 Hz, 2H), 7.25 (m, 1H), 6.81 (m, 1H), 6.76 (m, 1H), 5.21 (s, 2H), 4.52 (m, 1H), 4.24 (m, 2H), 3.79 (t, J = 5.5 Hz, 2H), 3.44 (m, 3H), 3.25 (m, 1H), 2.99 (m, 1H), 2.78 (d, J = 11.3 Hz, 1H), 2.65 (m, 1H), 2.56 (s, 3H), 2.52 (m, 2H); ^{13}C-NMR (150 MHz, d6-DMSO) δ 165.4, 161.1, 161.0, 154.4, 154.0, 152.2, 151.2, 148.0, 142.4, 141.9, 130.3, 126.3, 125.6, 124.3, 123.9, 121.8, 119.3, 116.3, 115.3, 105.6, 64.7, 63.2, 50.7, 50.2, 49.6, 47.2, 42.0, 24.6, 14.2; HRMS (m/z): $[M+H]^+$ calcd for $C_{31}H_{29}F_5N_7O_5$, 674.21448; found, 674.21507.

2-(2-(3,6-dihydro-2H-pyran-4-yl)-5-ethyl-6-(4-(3-hydroxypicolinoyl)piperazin-1-yl)-7-oxo-[1,2,4]triazolo[1,5-a]pyrimidin-4(7H)-yl)-N-(2-methyl-4-(trifluoromethyl)phenyl)acetamide, **3**
3-Hydroxypicolinic acid (110 mg, 778 µmol) was dissolved in DCM (5 mL) at RT under argon. 1-Chloro-N,N,2-trimethylprop-1-en-1-amine (113 µL, 856 µmol) was added and the RM was stirred at RT for 1.75 hours. A solution of 2-(2-(3,6-dihydro-2H-pyran-4-yl)-5-ethyl-7-oxo-6-(piperazin-1-yl)-[1,2,4]triazolo[1,5-a]pyrimidin-4(7H)-yl)-N-(2-methyl-4-(trifluoromethyl)phenyl)acetamide (Intermediate AP) (312 mg, 389 µmol) in DCM (2 mL) and DIPEA (340 µL, 1.94 mmol) were added and the RM was stirred at RT for 2.5 hours. The RM was quenched with water (5 mL) and aq sat $NaHCO_3$ (5 mL) and extracted with DCM (4 x 20 mL). The combined organic layers were washed with aq sat $NaHCO_3$ (5 mL) and water, dried over a phase separator and concentrated under reduced pressure. The crude product was adsorbed onto Isolute and purified by column chromatography (RediSep column: silica 24 g, eluent DCM:MeOH 100:0 to 90:10). The product containing fractions were combined and concentrated under reduced pressure. The still impure product was purified in 2 portions by reverse phase preparative HPLC (RP-HPLC acidic 1: 10 to 90% B in 20 min and RP-HPLC acidic 1: 20 to 80% B in 20 min). The product containing fractions were combined, basified with aq sat $NaHCO_3$ and the ACN was removed under reduced pressure. The residue was extracted with DCM (3 x 10 mL), dried through a phase separator and concentrated under reduced pressure to give **3** as a pale-yellow solid (118 mg, purity 99%, yield 45%).

LC-MS: Rt = 1.00 min; MS m/z $[M+H]^+$ 667.4, m/z $[M-H]^-$ 665.4; UPLC-MS 4; ^1H NMR (400 MHz, DMSO-d_6) δ 10.39 (s, 1H), 10.01 (s, 1H), 8.06 (m, 1H), 7.72 (d, J = 8.4 Hz, 1H), 7.63 (m, 1H), 7.53 (d, J = 8.6 Hz, 1H), 7.29 (m, 2H), 6.83 (m, 1H), 5.24 (s, 2H), 4.55 (m, 1H), 4.26 (m, 2H), 3.81 (m, 2H), 3.44 (m, 3H), 3.22 (m, 1H), 2.99 (m, 3H), 2.80 (m, 1H), 2.62 (m, 1H), 2.54 (m, 2H), 2.35 (s, 3H), 1.20 (t, J = 7.4 Hz, 3H); ^{13}C-NMR (150 MHz, DMSO-d_6): δ 165.8, 165.4, 161.0, 156.7, 154.1, 151.3, 150.7, 142.4, 139.7, 139.4, 132.1, 130.1, 127.2, 125.7, 125.5, 125.0, 124.4, 124.2, 123.3, 123.2, 121.4, 64.7, 63.1, 50.3, 50.2, 49.8, 46.9, 41.7, 24.6, 21.0, 17.7, 12.8; HRMS (m/z): $[M+H]^+$ calcd for $C_{32}H_{34}F_3N_8O_5$, 667.25988; found, 667.26044.

tert-butyl 4-(6-(4-acetylpiperazin-1-yl)-4-(2-((4-chlorophenyl)amino)-2-oxoethyl)-5-methyl-7-oxo-4,7-dihydro-[1,2,4]triazolo[1,5-*a*]pyrimidin-2-yl)piperazine-1-carboxylate, **5**

tert-Butyl 4-(6-bromo-4-(2-((4-chlorophenyl)amino)-2-oxoethyl)-5-methyl-7-oxo-4,7-dihydro-[1,2,4]triazolo[1,5-*a*]pyrimidin-2-yl)piperazine-1-carboxylate (Intermediate CO) (61.0 mg, 100 µmol), 1-(piperazin-1-yl)ethanone (25.6 mg, 200 µmol) and KF (29.0 mg, 499 µmol) were heated in DMSO (0.5 mL) with stirring at 100 °C for 45 min. 1-(Piperazin-1-yl)ethanone (220 mg) was added and the mixture was stirred at 120 °C for a further 20 min. 1-(Piperazin-1-yl)ethanone (150 mg) was added and the mixture was stirred at 120 °C for 15 min. The crude product was filtered and purified by reverse phase preparative HPLC (RP-HPLC acidic 1: 30 to 55% B in 20 min, 25 mL/min). Product containing fractions were partitioned between DCM (30 ml) and water (20 ml). The organic layer was separated by filtering through a phase separation tube, dried ($MgSO_4$), filtered and evaporated to give **5** as a pale-cream colored solid (14.5 mg, purity 95%, yield 22%).

LC-MS: Rt = 1.04 min; MS m/z $[M+H]^+$ 628.5, m/z $[M-H]^-$ 626.4; UPLC-MS 8; ^1H NMR (400 MHz, Methanol-d_4) δ 10.37 (s, 1H), 7.59 (d, J = 8.4 Hz, 2H), 7.35 (d, J = 8.5 Hz, 2H), 5.16 (s, 2H), 4.52 (d, J = 12.8 Hz, 1H), 3.93 (d, J = 12.9 Hz, 1H), 3.64 (t, J = 11.8 Hz, 1H), 3.52 (m, 9H), 3.40 (m, 1H), 2.88-2.72 (m, 3H), 2.62 (s, 3H), 2.17 (s, 3H), 1.49 (s, 9H); ^{13}C-NMR (100 MHz, Methanol-d_4): δ 170.5, 165.3, 164.5, 155.1, 154.5, 151.0, 150.7, 136.9, 129.3, 128.7, 122.5, 121.2, 80.3, 50.2, 49.9, 49.7, 47.0, 45.4, 42.5, 27.4, 20.0, 13.0; HRMS (m/z): $[M+H]^+$ calcd for $C_{29}H_{39}ClN_9O_5$, 628.27572; found, 628.27562.

2-(2,6-bis(4-acetylpiperazin-1-yl)-5-methyl-7-oxo-[1,2,4]triazolo[1,5-*a*]pyrimidin-4(7*H*)-yl)-*N*-(4-(trifluoromethyl)phenyl-2,6-t_2)acetamide, **6**

Step 1: 1-(4-(5-amino-4H-1,2,4-triazol-3-yl)piperazin-1-yl)ethan-1-one

1-Acetylpiperazine (67.0 g, 512 mmol) and dimethyl cyanocarbonimidodithioate (83.0 g, 512 mmol) were dissoved in acetonitrile (1.11 L), and the dark yellow solution was stirred at reflux for 3 hours. The reaction mixture was cooled to RT, and hydrazine hydrate (46.6 g, 45.3 mL, 512 mmol) was added dropwise over 15 min while keeping the temperature between 25 and 30 °C with the help of an ice bath (exothermic reaction). The colorless suspension was stirred at RT for 1 hour and then at 40 °C for another 1 hour. After cooling to RT, the precipitate was collected by filtration and washed with acetonitrile (500 mL) and dried under vacuum overnight at 40 °C to give the title compound as a colorless powder (108 g, purity 95%, yield 95%). LC-MS: Rt = 0.54 min; MS m/z $[M+H]^+$ 211.1, m/z $[M-H]^-$ 209.1; UPLC-MS 7.

Step 2: ethyl 2-(4-acetylpiperazin-1-yl)-3-oxobutanoate

To a yellow solution of 1-acetylpiperazine (105 g, 808 mmol) in toluene (808 mL) was added ethyl 2-chloro-3-oxobutanoate (70.0 g, 58.8 mL, 404 mmol). The solution was stirred at 100 °C for 2 hours. The RM was filtered through Hyflo and the residue was washed with toluene. The filtrate was evaporated. The brown oil was stirred in DCM (200 mL) for 1 hour, filtered and the residue was washed with DCM. The filtrate was concentrated under reduced pressure. The crude product was purified by column chromatography (RediSep Column: Silica 330 g, eluent EtOAc). The product containing fractions were combined, concentrated, and dried under HV to give the title compound as a yellow resin (85.0 g, purity 90%, yield 74%). LC-MS: Rt = 0.60/0.87 min; MS m/z $[M+H]^+$ 257.2, m/z $[M-H]^-$ 255.1; UPLC-MS 8.

Step 3: N-(2,6-dibromo-4-(trifluoromethyl)phenyl)-2-iodoacetamide

A mixture of 2-iodoacetyl chloride (1.28 g, 562 µL, 6.27 mmol) and 2,6-dibromo-4-(trifluoromethyl)aniline (200 mg, 627 µmol) was stirred under an argon atmosphere for 3 hours at RT. Ethyl acetate (25 mL) was added, and the organic phase was washed with aq sat $NaHCO_3$ and brine, dried over Na_2SO_4 and concentrated under reduced pressure. The crude product was purified by column chromatography (silica column, eluent heptane:EtOAc 100:0 to 0:100). Product containing fractions were combined and concentrated to give the title compound as a colorless solid (130 mg, purity 98%, yield 42%). LC-MS: Rt = 1.05 min; MS m/z $[M-H]^-$ 483.7/485.7; UPLC-MS 8.

Step 4: 1,1'-((5-methyl-7-oxo-4,7-dihydro-[1,2,4]triazolo[1,5-a]pyrimidine-2,6-diyl)bis(piperazine-4,1-diyl))bis(ethan-1-one)

1-(4-(5-Amino-4H-1,2,4-triazol-3-yl)piperazin-1-yl)ethan-1-one (820 mg, 3.90 mmol) and ethyl 2-(4-acetylpiperazin-1-yl)-3-oxobutanoate (1.00 g, 3.90 mmol) were dissolved in AcOH (10 mL) and the mixture was stirred at 120 °C for 5 hours and then concentrated under reduced pressure. Ethyl acetate (30 mL) was added, and the organic phase was washed with aq sat

Na_2CO_3 and brine. As no product could be detected in the organic phase, the aqueous layers were combined, acidified with citric acid, and extracted with DCM (3 x 10 mL). The organic layers were dried over Na_2SO_4 and concentrated under reduced pressure. The crude product was purified by column chromatography (silica column, eluent DCM:MeOH 100:0 to 90:10). Product containing fractions were combined and concentrated to give the title compound as a colorless foam (351 mg, purity 85%, yield 20%). LC-MS: Rt = 0.50 min; MS m/z $[M+H]^+$ 403.6, m/z $[M-H]^-$ 401.6; UPLC-MS 8.

Step 5: 2-(2,6-bis(4-acetylpiperazin-1-yl)-5-methyl-7-oxo-[1,2,4]triazolo[1,5-a]pyrimidin-4(7H)-yl)-N-(2,6-dibromo-4-(trifluoromethyl)phenyl)acetamide

N-(2,6-dibromo-4-(trifluoromethyl)phenyl)-2-iodoacetamide (63.9 mg, 162 µmol) and K_2CO_3 (51.5 mg, 373 µmol) were added to a solution of 1,1'-((5-methyl-7-oxo-4,7-dihydro-[1,2,4]triazolo[1,5-a]pyrimidine-2,6-diyl)bis(piperazine-4,1-diyl))bis(ethan-1-one) (50.0 mg, 124 µmol) in DMF (1 mL) and the mixture was stirred at 80 °C for 6 hours. N-(2,6-dibromo-4-(trifluoromethyl)phenyl)-2-iodoacetamide (24.6 mg, 62.2 µmol) was added, and stirring was continued for 24 hours at 60 °C. After cooling to RT, ethyl acetate (20 mL) was added, and the organic phase was washed with aq sat $NaHCO_3$ and brine, dried over Na_2SO_4 and concentrated under reduced pressure. The crude product was purified by column chromatography (silica column, eluent DCM:MeOH 100:0 to 90:10). Product containing fractions were combined and concentrated to give the title compound as a colorless solid (41.8 mg, purity 98%, yield 43%). LC-MS: Rt = 0.88 min; MS m/z $[M+H]^+$ 760.1/762.2, m/z $[M-H]^-$ 758.1/760.0; UPLC-MS 8.

Step 6: 2-(2,6-bis(4-acetylpiperazin-1-yl)-5-methyl-7-oxo-[1,2,4]triazolo[1,5-a]pyrimidin-4(7H)-yl)-N-(4-(trifluoromethyl)phenyl-2,6-t2)acetamide

4.94 mg (6.49 µmol) of 2-(2,6-bis(4-acetylpiperazin-1-yl)-5-methyl-7-oxo-[1,2,4]triazolo[1,5-a]pyrimidin-4(7H)-yl)-N-(2,6-dibromo-4-(trifluoromethyl)phenyl)acetamide, 9.80 mg Pd/C (10%) and 6 µL (4.38 mg, 43.3 µmol) triethylamine were suspended in 0.3 ml of DMF. The suspension was degassed three times at the high vacuum manifold and stirred under an atmosphere of tritium gas (7.1 Ci, 676 mbar initial pressure) for 44 min at RT (end pressure was 522 mbar, the pressure in the reaction vessel was constant). The solvent was removed in vacuo, and labile tritium was exchanged by adding 0.3 ml of methanol, stirring the solution, and removing the solvent again under vacuum. This process was repeated two times. Finally, the well dried solid was extracted with 5 ml of ethanol and the suspension was filtered through a 0.2 µm nylon membrane (Macherey-Nagel Polyamide syringe filter CHRO-MAFIL® Xtra PA-20/25), obtaining a clear and colourless solution. The RCP of the crude product was determined to 96% using the following HPLC system: Waters Sunfire C18, 5 µm, 4.6 x 250

mm; solvents A: water + 0.05% TFA, B: acetonitrile + 0.05% TFA; 0 min 10% B; 10 min 95% B; 14.5 min 95% B; 15 min 10% B; 254 nm; 1.0 ml/min; 30 °C.

The crude product (269 mCi) was concentrated at the rotatory evaporator and the residue was dissolved in 0.15 ml of ethanol. Purification of the crude product was carried out using the following conditions: Waters Sunfire C18, 5 µm, 10 x 250 mm; solvents A: water + 0.1% TFA, B: acetonitrile + 0.1% TFA; isocratic 42% B; 254 nm; 4.7 ml/min; 25 °C. The target compound eluted at 8.3 min.

The desired product was isolated from the HPLC solvent mixture by solid phase extraction. Therefore, the HPLC solution was neutralized with an aqueous solution of $NaHCO_3$ and the volume of the fractions were partially reduced at the rotary evaporator. Then, the product was extracted with a Phenomenex StrataX cartridge (33 µm Polymeric Reversed Phase, 100 mg, 3 ml) which was eluted with 5 ml of ethanol. The extracted product with an activity of 196.6 mCi (7.27 GBq) showed an RCP of >99% and the SA (determined by MS) was determined to 54.8 Ci/mmol (2.03 TBq/mmol). RP-HPLC acidic 2: Rt = 8.8 min (UV, radio detector).

Intermediates

Intermediate AF: tert-butyl 4-(2-(3,6-dihydro-2H-pyran-4-yl)-5-ethyl-7-oxo-4,7-dihydro-[1,2,4]triazolo[1,5-a]pyrimidin-6-yl)piperazine-1-carboxylate

To a stirred solution of tert-butyl 4-(2-bromo-5-ethyl-7-oxo-4,7-dihydro-[1,2,4]triazolo[1,5-a]pyrimidin-6-yl)piperazine-1-carboxylate (Intermediate X) (15.0 g, 35.1 mmol) in 1,4-dioxane (150 mL) and water (50 mL) was added 2-(3,6-dihydro-2H-pyran-4-yl)-4,4,5,5-tetramethyl-1,3,2-dioxaborolane (11.1 g, 52.7 mmol) and Na_2CO_3 (7.44 g, 70.2 mmol). The RM was degassed with nitrogen for 15 minutes. Pd(dppf)Cl_2.DCM (1.43 g, 1.76 mmol) was added and the RM was stirred at 100 °C for 14 hours. Water (300 mL) was added and the RM was extracted with 10% MeOH in DCM (2 x 500 mL). The organic layer was washed with brine (300 mL), dried over Na_2SO_4 and concentrated under reduced pressure. The crude product was purified by column chromatography (silica gel column: silica 40 g, eluent DCM:MeOH 100:0 to 97:3). The product containing fractions were combined, concentrated under vacuum and dried under HV to give the title compound (10.8 g, purity 97%, yield 69%).

LC-MS: Rt = 0.96 min; MS m/z $[M+H]^+$ 431.4, m/z $[M-H]^-$ 429.3; UPLC-MS 3

^{1}H NMR (400 MHz, DMSO-d_6) δ 13.00 (s, br, 1H), 6.81 (m, 1H), 4.28 (m, 2H), 3.92 (m, 2H), 3.82 (m, 2H), 3.37 (m, 2H), 2.89 (m, 2H), 2.76 (m, 2H), 2.62 (m, 2H), 2.51 (m, 2H), 1.43 (s, 9H), 1.19 (t, J = 7.3 Hz, 3H).

Intermediate AK: N-(2-chloro-4-(trifluoromethyl)phenyl)-2-(2-(3,6-dihydro-2H-pyran-4-yl)-5-ethyl-7-oxo-6-(piperazin-1-yl)-[1,2,4]triazolo[1,5-a]pyrimidin-4(7H)-yl)acetamide

Step 1: tert-butyl 4-(4-(2-((2-chloro-4-(trifluoromethyl)phenyl)amino)-2-oxoethyl)-2-(3,6-dihydro-2H-pyran-4-yl)-5-ethyl-7-oxo-4,7-dihydro-[1,2,4]triazolo[1,5-a]pyrimidin-6-yl)piperazine-1-carboxylate

Tert-butyl 4-(2-(3,6-dihydro-2H-pyran-4-yl)-5-ethyl-7-oxo-4,7-dihydro-[1,2,4]triazolo[1,5-a]pyrimidin-6-yl)piperazine-1-carboxylate (Intermediate AF) (6.50 g, 15.1 mmol) and N-(2-chloro-4-(trifluoromethyl)phenyl)-2-iodoacetamide (Intermediate DL) (6.04 g,16.6 mmol) were mixed in DMF (72 mL) at 0 °C. DIPEA (7.91 mL, 45.3 mmol) was added and the RM was stirred at 45 °C for 3.5 hours. The RM was cooled to RT. Water (70 mL) was added and the suspension was stirred at RT overnight. The suspension was sonicated for 25 minutes and filtered. The cake was washed with a small amount of water and dried. The filtrate was filtered again. The second filtrate was extracted with EtOAc (2 x 400 mL), washed with brine (2 x 50 mL), dried through a phase separator and concentrated under reduced pressure. The 2 cakes were adsorbed onto Isolute and purified by column chromatography (RediSep column: silica 220 g, eluent DCM:DCM/MeOH (1/1) 100:0 to 80:20). The pure product containing fractions were combined and concentrated under reduced pressure. The beige solid foam was dissolved in Et_2O and the resulting crystals were sonicated. The suspension was left standing overnight, filtered, washed with a small amount of Et_2O and dried under HV to give cake 1 as a white solid. The impure fractions were combined and concentrated under reduced pressure. Then they were combined with the concentrated organic layer from the extraction and purified by column chromatography again (RediSep column: silica 120 g Gold, eluent DCM:DCM/MeOH (1/1) 100:0 to 85:15). The product containing fractions were combined, concentrated under reduced pressure, and dried under HV. The beige solid foam was crystallized out of Et_2O to give cake 2 as a white solid. Cake 1 and cake 2 were combined to give the title compound (6.41 g, purity 99%, yield 63%).

LC-MS: Rt = 1.33 min; MS m/z $[M+H-Boc]^+$ 566.0/568.0, m/z $[M+H]^+$ 666.0/668.0, m/z $[M-H]^-$ 664.1/666.1; UPLC-MS 1

Step 2: N-(2-chloro-4-(trifluoromethyl)phenyl)-2-(2-(3,6-dihydro-2H-pyran-4-yl)-5-ethyl-7-oxo-6-(piperazin-1-yl)-[1,2,4]triazolo[1,5-a]pyrimidin-4(7H)-yl)acetamide

Tert-butyl 4-(4-(2-((2-chloro-4-(trifluoromethyl)phenyl)amino)-2-oxoethyl)-2-(3,6-dihydro-2H-pyran-4-yl)-5-ethyl-7-oxo-4,7-dihydro-[1,2,4]triazolo[1,5-a]pyrimidin-6-yl)piperazine-1-carboxylate (6.41 g, 9.62 mmol) was dissolved in DCM (70 mL) and TFA (11.1 mL, 144 mmol) was added. The RM was stirred at RT for 1 hour. The RM was concentrated under reduced pressure. The residue was dissolved in DCM and concentrated under reduced pressure again. This was performed three times. The resulting oil was dried under HV to result in a pale rose solid foam. The foam was suspended in Et_2O and sonicated. The suspension was filtered, washed with Et_2O and dried under HV to give the title compound as a white solid (6.68 g, purity 81%, yield 99%).

LC-MS: Rt = 0.78 min; MS m/z $[M+H]^+$ 566.4/568.4, m/z $[M-H]^-$ 564.2/566.2; UPLC-MS 1

Intermediate AP: 2-(2-(3,6-dihydro-2H-pyran-4-yl)-5-ethyl-7-oxo-6-(piperazin-1-yl)-[1,2,4]triazolo[1,5-a]pyrimidin-4(7H)-yl)-N-(2-methyl-4-(trifluoromethyl)phenyl)acetamide

Step 1: tert-butyl 4-(2-bromo-5-ethyl-4-(2-((2-methyl-4-(trifluoromethyl)phenyl)amino)-2-oxoethyl)-7-oxo-4,7-dihydro-[1,2,4]triazolo[1,5-a]pyrimidin-6-yl)piperazine-1-carboxylate

To a stirred solution of tert-butyl 4-(2-bromo-5-ethyl-7-oxo-4,7-dihydro-[1,2,4]triazolo[1,5-a]pyrimidin-6-yl)piperazine-1-carboxylate (Intermediate X) (15.0 g, 33.3 mmol) and 2-iodo-N-(2-methyl-4-(trifluoromethyl)phenyl)acetamide (Intermediate DV) (13.2 g, 38.4 mmol) in 1,4-dioxane (200 mL) was added DIPEA (17.5 mL, 100 mmol) at RT and the RM was stirred at 80 °C for 15 minutes. The RM was concentrated under reduced pressure. The crude product was diluted with EtOAc and water, extracted once with EtOAc and the organic layer was washed with brine, dried over Na_2SO_4 and concentrated under reduced pressure. The crude product was purified by column chromatography (eluent heptane:EtOAc 70:30 to 0:100). The product containing fractions were combined, concentrated and triturated in Et_2O to give the title compound as a white solid (19.7 g, purity 98%, yield 90%).

LC-MS: Rt = 1.31 min; MS m/z $[M+H-Boc]^+$ 542.2, m/z $[M-H]^-$ 640.5; UPLC-MS 1

Step 2: tert-butyl 4-(2-(3,6-dihydro-2H-pyran-4-yl)-5-ethyl-4-(2-((2-methyl-4-(trifluoromethyl)phenyl)amino)-2-oxoethyl)-7-oxo-4,7-dihydro-[1,2,4]triazolo[1,5-a]pyrimidin-6-yl)piperazine-1-carboxylate

To a stirred solution of tert-butyl 4-(2-bromo-5-ethyl-4-(2-((2-methyl-4-(trifluoromethyl)phenyl)amino)-2-oxoethyl)-7-oxo-4,7-dihydro-[1,2,4]triazolo[1,5-a]pyrimidin-6-yl)piperazine-1-carboxylate (11.2 g, 17.4 mmol), K_3PO_4 (11.1 g, 52.2 mmol) and XPhos Pd G3 (736 mg, 870 µmol) in 1,4-dioxane (100 mL) and water (50 mL) was added dropwise at 80 °C a solution of 2-(3,6-dihydro-2H-pyran-4-yl)-4,4,5,5-tetramethyl-1,3,2-dioxaborolane (4.75 g, 22.6 mmol) in 1,4-dioxane (50 mL) and the RM was stirred at 80 °C for 30 minutes. The RM was diluted with DCM and water, extracted twice with DCM and the combined organic extracts were washed with brine, dried over Na_2SO_4, concentrated under reduced pressure and triturated in Et_2O to give the title compound as a white solid (9.83 g, purity 99%, yield 87%).

LC-MS: Rt = 1.26 min; MS m/z $[M+H-Boc]^+$ 546.4, m/z $[M-H]^-$ 644.3; UPLC-MS 1

Step 3: 2-(2-(3,6-dihydro-2H-pyran-4-yl)-5-ethyl-7-oxo-6-(piperazin-1-yl)-[1,2,4]triazolo[1,5-a]pyrimidin-4(7H)-yl)-N-(2-methyl-4-(trifluoromethyl)phenyl)acetamide

HCl 4N in 1,4-dioxane (30.0 mL, 120 mmoL) was added to tert-butyl 4-(2-(3,6-dihydro-2H-pyran-4-yl)-5-ethyl-4-(2-((2-methyl-4-(trifluoromethyl)phenyl)amino)-2-oxoethyl)-7-oxo-4,7-dihydro-[1,2,4]triazolo[1,5-a]pyrimidin-6-yl)piperazine-1-carboxylate (9.83 g, 15.2 mmol) and the RM was stirred at 0 °C for 2 hours. The RM was diluted with DCM and $NaHCO_3$, extracted three times with 10% MeOH in DCM and the combined organic layers were dried over Na_2SO_4,

concentrated under reduced pressure and dried to give the title compound as a white solid (8.29 g, purity 99%, yield 99%).
LC-MS: Rt = 0.75 min; MS m/z $[M+H]^+$ 546.3, m/z $[M-H]^-$ 544.4; UPLC-MS 1

Intermediate BM: tert-butyl 4-(2-bromo-5-methyl-7-oxo-4,7-dihydro-[1,2,4]triazolo[1,5-a]pyrimidin-6-yl)piperazine-1-carboxylate
3-Bromo-1H-1,2,4-triazol-5-amine (Intermediate ER) (4.00 g, 24.5 mmol), tert-butyl 4-(1-ethoxy-1,3-dioxobutan-2-yl)piperazine-1-carboxylate (Intermediate EP) (8.49 g, 27.0 mmol) and H_3PO_4 (2.97 g, 25.8 mmol) were mixed in EtOH (25 mL) and stirred at reflux for 18 hours. The RM was cooled to RT, DIPEA (12.9 mL, 73.6 mmol) and Boc_2O (1.71 mL, 7.36 mmol) were added, and the RM was stirred at RT for 1 hour. The RM was quenched with aq NH_4Cl, diluted with DCM, extracted twice with DCM, dried over Na_2SO_4, concentrated and dried. The crude product was crystallized from DCM and TBME to give the title compound (6.94 g, purity 99%, yield 68%).
LC-MS: Rt = 0.87 min; MS m/z $[M+H]^+$ 413.1, m/z $[M-H]^-$ 411.0; UPLC-MS 4

Intermediate BN: 2-(2-(3,6-dihydro-2H-pyran-4-yl)-5-methyl-7-oxo-6-(piperazin-1-yl)-[1,2,4]triazolo[1,5-a]pyrimidin-4(7H)-yl)-N-(4-(trifluoromethyl)phenyl)acetamide
Step 1: tert-butyl 4-(2-bromo-5-methyl-7-oxo-4-(2-oxo-2-((4-(trifluoromethyl)phenyl)amino)ethyl)-4,7-dihydro-[1,2,4]triazolo[1,5-a]pyrimidin-6-yl)piperazine-1-carboxylate
Tert-butyl 4-(2-bromo-5-methyl-7-oxo-4,7-dihydro-[1,2,4]triazolo[1,5-a]pyrimidin-6-yl)piperazine-1-carboxylate (Intermediate BM) (7.38 g, 17.9 mmol), 2-bromo-N-(4-(trifluoromethyl)phenyl)acetamide (6.04 g, 21.4 mmol) and DIPEA (9.36 mL, 53.6 mmol) were dissolved in DMF (50 mL) and the RM was stirred at 80 °C for 2 hours. The RM was cooled to RT, diluted with DCM, and the organic phase was extracted with aq sat $NaHCO_3$ and brine, dried over Na_2SO_4 and concentrated under reduced pressure. The crude product was purified by column chromatography (eluent heptane:EtOAc/MeOH (9/1) 100:0 to 30:70). The product containing fractions were combined and concentrated under reduced pressure and then crystallized from TBME to give the title compound (7.75 g, purity 98%, yield 69%).
LC-MS: Rt = 1.15 min; MS m/z $[M+H]^+$ 614.0, m/z $[M-H]^-$ 612.0; UPLC-MS 4
Step 2: tert-butyl 4-(2-(3,6-dihydro-2H-pyran-4-yl)-5-methyl-7-oxo-4-(2-oxo-2-((4-(trifluoro methyl)phenyl)amino)ethyl)-4,7-dihydro-[1,2,4]triazolo[1,5-a]pyrimidin-6-yl)piperazine-1-carboxylate
Tert-butyl 4-(2-bromo-5-methyl-7-oxo-4-(2-oxo-2-((4-(trifluoromethyl)phenyl)amino)ethyl)-4,7-dihydro-[1,2,4]triazolo[1,5-a]pyrimidin-6-yl)piperazine-1-carboxylate (7.70 g, 12.5 mmol), 2-(3,6-dihydro-2H-pyran-4-yl)-4,4,5,5-tetramethyl-1,3,2-dioxaborolane (3.95 g, 18.8 mmol),

Tert-butyl 4-(2-bromo-5-methyl-7-oxo-4-(2-oxo-2-((4-(trifluoromethyl)phenyl)amino)ethyl)-4,7-dihydro-[1,2,4]triazolo[1,5-a]pyrimidin-6-yl)piperazine-1-carboxylate (7.70 g, 12.5 mmol), 2-(3,6-dihydro-2H-pyran-4-yl)-4,4,5,5-tetramethyl-1,3,2-dioxaborolane (3.95 g, 18.8 mmol), K_3PO_4 1.5N (20.9 mL, 31.3 mmol) and XPhos Pd G3 (1.06 g, 1.25 mmol) were dissolved in 1,4-dioxane (50 mL). The RM was stirred at 90 °C for 1 hour and after cooling diluted with EtOAc. The organic phase was washed with aq sat $NaHCO_3$ and brine, dried over Na_2SO_4 and concentrated under reduced pressure. This material was dissolved in DCM/MeOH (1:1) and ISOLUTE® Si-Thiol (258 mg) was added. After stirring for 30 minutes, the mixture was filtered and concentrated. The crude product was crystallized from DCM and TBME to give the title compound (5.93 g, purity 98%, yield 75%).

LC-MS: Rt = 1.13 min; MS m/z $[M+H]^+$ 618.2, m/z $[M-H]^-$ 616.1; UPLC-MS 4

<u>Step 3:</u> 2-(2-(3,6-dihydro-2H-pyran-4-yl)-5-methyl-7-oxo-6-(piperazin-1-yl)-[1,2,4]triazolo[1,5-a]pyrimidin-4(7H)-yl)-N-(4-(trifluoromethyl)phenyl)acetamide

Tert-butyl 4-(2-(3,6-dihydro-2H-pyran-4-yl)-5-methyl-7-oxo-4-(2-oxo-2-((4-(trifluoromethyl) phenyl)amino)ethyl)-4,7-dihydro-[1,2,4]triazolo[1,5-a]pyrimidin-6-yl)piperazine-1-carboxylate (5.95 g, 9.63 mmol) was dissolved in DCM (50 mL) and TFA (22.3 mL, 289 mmol) was added. The RM was stirred at RT for 1 hour and then concentrated under reduced pressure. Toluene was added and removed again, and this procedure was repeated. The residue was dissolved in EtOAc and washed with aq sat $NaHCO_3$ and brine. During the extraction the product crystallized, and the solids were collected and dried to give the title compound (5.08 g, purity 98%, yield 99%).

LC-MS: Rt = 0.84 min; MS m/z $[M+H]^+$ 518.2, m/z $[M-H]^-$ 516.0; UPLC-MS 4

<u>Intermediate CO:</u> *tert*-butyl 4-(6-bromo-4-(2-((4-chlorophenyl)amino)-2-oxoethyl)-5-methyl-7-oxo-4,7-dihydro-[1,2,4]triazolo[1,5-*a*]pyrimidin-2-yl)piperazine-1-carboxylate

To *tert*-butyl 4-(4-(2-((4-chlorophenyl)amino)-2-oxoethyl)-5-methyl-7-oxo-4,7-dihydro-[1,2,4]triazolo[1,5-*a*]pyrimidin-2-yl)piperazine-1-carboxylate (Intermediate CP) (11.4 g, 19.9 mmol) in AcOH (200 mL) was added portionwise over 5 min NBS (3.89 g, 21.9 mmol) and the resulting mixture was stirred at RT for 15 min. LCMS indicated approx. 90-95% conversion to the desired product. Allowed to stir for a further 10 min, then diluted with water (100 ml), stirred for 5 min, filtered and washed with water (30 mL). Dried on filter pad, followed by 72 h at 40 °C under vacuum to give Intermediate CO as a colourless solid (8.96 g, purity 90%, yield 66%).

LC-MS: Rt = 1.12 min; MS m/z $[M+H]^+$ 580.2, m/z $[M-H]^-$ 578.3; UPLC-MS 8

<u>Intermediate CP:</u> *tert*-butyl 4-(4-(2-((4-chlorophenyl)amino)-2-oxoethyl)-5-methyl-7-oxo-4,7-dihydro-[1,2,4]triazolo[1,5-*a*]pyrimidin-2-yl)piperazine-1-carboxylate

To *tert*-butyl 4-(5-methyl-7-oxo-4,7-dihydro-[1,2,4]triazolo[1,5-*a*]pyrimidin-2-yl)piperazine-1-carboxylate (20.1 g, 60.2 mmol), *N*-(4-chlorophenyl)-2-iodoacetamide (19.6 g, 66.2 mmol) and K_2CO_3 (9.81 g, 71.0 mmol) was added DMF (200 mL) and the resulting mixture was heated to 50 °C for 50 min. Added *N*-(4-chlorophenyl)-2-iodoacetamide (1.81 g) and K_2CO_3 (980 mg) and continued stirring at 50 °C for 10 min. Then added *N*-(4-chlorophenyl)-2-iodoacetamide (1.05 g) and K_2CO_3 (630 mg) and continued at 50 °C for 20 min. While stirring, diluted with water (300 ml) and allowed to cool to RT with stirring for 10 min. Filtered and the filter pad washed with diethyl ether (200 ml), dried to give Intermediate CP as a colourless solid (31.8 g, purity 98%, 90% yield).
LC-MS: Rt = 1.01 min; MS m/z $[M+H]^+$ 502.1, m/z $[M-H]^-$ 500.3; UPLC-MS 8

Intermediate CY: 5-(benzyloxy)-6-methylpyrimidine-4-carboxylic acid
Step 1: 5-(benzyloxy)-4,6-dichloropyrimidine
To a stirred solution of 4,6-dichloropyrimidin-5-ol (29.5 g, 179 mmol) in DMF (100 mL) was added K_2CO_3 (32.1 g, 232 mmol) then (bromomethyl)benzene (23.4 mL, 197 mmol) at RT and the RM was stirred at RT for 3 hours. The RM was diluted with EtOAc/water, extracted once with EtOAc and the organic layer was washed with brine, dried over Na_2SO_4 and concentrated under reduced pressure. The crude product was purified by column chromatography (RediSep column: silica 330 g, eluent heptane:EtOAc 100:0 to 87:13). The product containing fractions were combined and concentrated under reduced pressure to afford the title compound as a white solid (45.1 g, purity 99%, yield 99%).
LC-MS: Rt = 1.18 min; no mass observed; UPLC-MS 1
Step 2: 5-(benzyloxy)-4-chloro-6-methylpyrimidine
To a stirred solution of 5-(benzyloxy)-4,6-dichloropyrimidine (32.5 g, 127 mmol), K_3PO_4 (81.0 g, 382 mmol) and Pd(dppf)Cl_2.DCM (5.20 g, 6.36 mmol) in toluene (350 mL) and water (100 mL) was added methyl boronic acid (9.17 g, 153 mmol) in 1,4-dioxane (45 mL) at 105 °C and the reaction was stirred at 105 °C for 18 hours. Methyl boronic acid (9.17 g, 153 mmol) was added again and the RM was stirred at 105 °C for 8 hours. Methyl boronic acid (9.17 g, 153 mmol) was added again and the RM was stirred at 105 °C for 4 hours. The RM was diluted with EtOAc/water, extracted twice with EtOAc and the combined organic layers were washed with water and brine, dried over Na_2SO_4 and concentrated under reduced pressure. The crude product was purified by column chromatography (RediSep column: silica 330 g, eluent heptane:EtOAc 100:0 to 77:23). The product containing fractions were combined and concentrated under reduced pressure to give the title compound as a yellow oil (21.4 g, purity 99%, yield 72%).
LC-MS: Rt = 1.03 min; MS m/z $[M+H]^+$ 235.2/237.2; UPLC-MS 1
Step 3: methyl 5-(benzyloxy)-6-methylpyrimidine-4-carboxylate

A solution of 5-(benzyloxy)-4-chloro-6-methylpyrimidine (25.9 g, 110 mmol), $Pd(dppf)Cl_2$.DCM (4.51 g, 5.52 mmol) and Et_3N (30.8 mL, 221 mmol) in MeOH (25 mL) was stirred at 50 °C under 10 bar of CO for 40 hours.The RM was filtered through celite and concentrated under reduced pressure. The residue was triturated with DCM and the solid was filtered off. The filtrate was purified by column chromatography (eluent heptane:EtOAc 100:0 to 60:40). The product containing fractions were combined and concentrated under reduced pressure to give the title compound as a white solid (13.0 g, purity 99%, yield 46%).

LC-MS: Rt = 0.81 min; MS m/z $[M+H]^+$ 259.1; UPLC-MS 1

Step 4: 5-(benzyloxy)-6-methylpyrimidine-4-carboxylic acid

To a stirred solution of methyl 5-(benzyloxy)-6-methylpyrimidine-4-carboxylate (22.9 g, 88.0 mmol) in THF (100 mL) and MeOH (100 mL) was added NaOH 2N in water (100 mL, 200 mmol) at RT and the RM was stirred at RT for 5 minutes. THF and MeOH were removed under reduced pressure, then the resulting aqueous residue was acidified to pH 3 with 2 N HCl and the mixture was filtered to give the title compound as a white solid (21.6 g, purity 99%, yield 99%).

LC-MS: Rt = 0.43 min; MS m/z $[M+H]^+$ 245.2, m/z $[M-H]^-$ 243.1; UPLC-MS 1

Intermediate DL: N-(2-chloro-4-(trifluoromethyl)phenyl)-2-iodoacetamide

Step 1: 2-chloro-N-(2-chloro-4-(trifluoromethyl)phenyl)acetamide

2-Chloro-4-(trifluoromethyl)aniline (18.5 g, 95.0 mmol) was dissolved in DCM (180 mL) at 0 °C. A solution of 2-chloroacetyl chloride (10.7 g, 95.0 mmol) in DCM (40 mL) was added dropwise over 15 minutes. After 30 minutes at 0 °C the RM was warmed to RT. The white suspension was stirred at RT overnight. The suspension was filtered and washed with DCM. The filtrate was concentrated under reduced pressure and dried under HV to give the title compound as a white solid (15.8 g, purity 99%, yield 62%).

LC-MS: Rt = 1.14 min; MS m/z $[M-H]^-$ 270.1/272.1/274.0; UPLC-MS 1

Step 2: N-(2-chloro-4-(trifluoromethyl)phenyl)-2-iodoacetamide

2-Chloro-N-(2-chloro-4-(trifluoromethyl)phenyl)acetamide (15.8 g, 58.2 mmol) was dissolved in acetone (215 mL), KI (10.6 g, 64.0 mmol) was added and the RM was stirred at reflux for 2.25 hours. The RM was cooled to RT and the suspension was filtered. The cake was washed with acetone and DCM. The filtrate was concentrated under reduced pressure and dried under HV to give the title compound (21.9 g, purity 97%, yield 99%).

LC-MS: Rt = 1.13 min; MS m/z $[M-H]^-$ 362.0/364.0; UPLC-MS 1

Intermediate DV: 2-iodo-N-(2-methyl-4-(trifluoromethyl)phenyl)acetamide

Step 1: 2-chloro-N-(2-methyl-4-(trifluoromethyl)phenyl)acetamide

2-Methyl-4-(trifluoromethyl)aniline (10.0 g, 56.0 mmol) was dissolved in DCM (93 mL) at 0 °C. Then 2-chloroacetyl chloride (4.72 mL, 58.7 mmol) was added, followed by Et_3N (17.1 mL, 123 mmol) (violet solution). After 30 minutes was the RM warmed to RT and it was stirred at RT for 1.2 hours. The RM was extracted with DCM (3 x 200 mL) and with water (2 x 60 mL). The organic layer was dried through a phase separator and concentrated under reduced pressure. The crude product was suspended in a small amount of DCM and filtered. The cake was washed with a small amount of DCM and Et_2O. The cake was dried under reduced pressure overnight. The filtrate was concentrated under reduced pressure. The residue was adsorbed onto Isolute and purified by column chromatography (RediSep column: silica 80 g, eluent heptane:EtOAc 50:50 to 0:100. The product containing fractions were combined and concentrated under reduced pressure and combined with the cake to give the title compound (11.8 g, purity 99%, yield 83%).

LC-MS: Rt = 1.01 min; MS m/z $[M-H]^-$ 250.1/252.1; UPLC-MS 4

Step 2: 2-iodo-N-(2-methyl-4-(trifluoromethyl)phenyl)acetamide

2-Chloro-N-(2-methyl-4-(trifluoromethyl)phenyl)acetamide (11.8 g, 46.8 mmol) was dissolved in acetone (173 mL) and KI (10.1 g, 60.9 mmol) was added. The RM was stirred at reflux for 2.5 hours. The RM was cooled to RT and the suspension was filtered. The cake was washed with acetone and DCM. The filtrate was concentrated under reduced pressure to give a beige solid. The solid was suspended in DCM and filtered to give the title compound as beige solid (15.8 g, purity 99%, yield 97%).

LC-MS: Rt = 1.04 min; MS m/z $[M-H]^-$ 342.0; UPLC-MS 4

Intermediate EH: tert-butyl 4-(1-methoxy-1,3-dioxopentan-2-yl)piperazine-1-carboxylate

Step 1: methyl 2-chloro-3-oxopentanoate

To a solution of methyl 3-oxopentanoate (10.4 kg, 80.0 mol) in DCM (67 L) was added SO_2Cl_2 (14.0 kg, 104 mol) at RT over 2.5 hours. The reaction was allowed to warm to RT and stirred for 16 hours. The RM was concentrated under reduced pressure and the residue was dissolved in DCM (20 L) and washed with water (10 L), brine (10 L), dried over Na_2SO_4 and filtered. The filtrate was concentrated under reduced pressure to give the title compound as a light-yellow liquid (14.4 kg, purity 73%, yield 80%).

^1H NMR (400 MHz, $CDCl_3$-*d*) δ 4.65 (s, 1H), 3.68 (s, 3H), 2.59 (m, 2H), 0.96 (t, 3H)

Step 2: tert-butyl 4-(1-methoxy-1,3-dioxopentan-2-yl)piperazine-1-carboxylate

To a solution of methyl 2-chloro-3-oxopentanoate (12.1 kg, 53.7 mol) in dry ACN (53 L) was added Et_3N (22.3 L, 161 mol) over 1.5 hours, followed by dropwise addition of tert-butyl piperazine-1-carboxylate (10.0 kg, 53.7 mol) in ACN (50 L) over 2.5 hours. The reaction was stirred at 60 °C for 16 hours. The RM was filtered and washed with EtOAc (10 L). The filtrate was then concentrated under reduced pressure and the residue was dissolved in EtOAc (45

L) and washed with water (45 L), dried over Na_2SO_4, and filtered. The filtrate was concentrated under reduced pressure and the residue was purified by column chromatography (silica column 150 mm x 800 mm x 70 mm, eluent heptane:EtOAc 100:0 to 90:10) to give the title compound (16.1 kg, purity 93%, yield 89%).
HPLC: Rt = 3.68/5.51 min; HPLC 4

Intermediate EP: tert-butyl 4-(1-ethoxy-1,3-dioxobutan-2-yl)piperazine-1-carboxylate
To a stirred solution of tert-butyl piperazine-1-carboxylate (150 g, 805 mmoL) in ACN (1.5 L) at RT was added K_2CO_3 (223 g, 1.61 mol) and the RM was stirred for 15 minutes. Then ethyl 2-chloro-3-oxobutanoate (112 mL, 809 mmol) was added slowly at the same temperature. The resulting RM was stirred at RT for 16 hours. The RM was filtered through celite pad. The celite pad was washed with EtOAc (2 L). The combined organic layers were concentrated under reduced pressure to get crude residue. The residue was dissolved in EtOAc (3 L) and then washed with ice cold water, brine, dried over Na_2SO_4 and concentrated under reduced pressure to get crude product as pale brown liquid. The crude product was purified by column chromatography (silica gel, 60-120 mesh, eluent petroleum ether:EtOAc 100:0 to 85:15). The pure product containing fractions were combined and concentrated under reduced pressure to give the title compound as a liquid. The impure fractions were combined and concentrated under reduced pressure. Then they were purified again by column chromatography (silica gel, 60-120 mesh, eluent petroleum ether:EtOAc 100:0 to 85:15). The pure product containing fractions were combined and concentrated under reduced pressure to give the title compound as a liquid. Both liquids were mixed, dissolved in DCM and concentrated under reduced pressure to get the title compound as a brown liquid. The liquid was again dissolved in DCM, concentrated under reduced pressure. This process was repeated three times and then the material was dried under vacuum to give the title compound as a brown liquid (230 g, purity 95%, yield 57%).
HPLC: Rt = 11.76 min; HPLC 6

Intermediate X: tert-butyl 4-(2-bromo-5-ethyl-7-oxo-4,7-dihydro-[1,2,4]triazolo[1,5-a]pyrimidin-6-yl)piperazine-1-carboxylate
3-Bromo-1H-1,2,4-triazol-5-amine (82.6 g, 507 mmol) and tert-butyl 4-(1-methoxy-1,3-dioxopentan-2-yl) piperazine-1-carboxylate (Intermediate EH) (175 g, 557 mmol) were mixed in EtOH (465 mL). H_3PO_4 (49.7 g, 507 mmol) was added. The mixture was stirred at 80 °C for 12 hours under nitrogen. The mixture was concentrated *in vacuo* to remove EtOH, then quenched by addition of aq sat $NaHCO_3$ (1 L) and extracted with DCM (3 x 1 L). The combined organic layers were washed with brine (3 x 1 L), dried over Na_2SO_4, filtered and concentrated under reduced pressure to give a residue. The residue was purified by column

chromatography (silica column, eluent DCM:MeOH 1:0 to 10:1). The product containing fractions were combined and concentrated under reduced pressure to give the title compound as a yellow solid (79.0 g, purity 98%, yield 36%).
LC-MS: Rt = 0.91 min; MS m/z $[M+H-Boc]^+$ 327.1/329.1, m/z $[M+H]^+$ 427.2/429.2, m/z $[M-H]^-$ 425.2/427.2; UPLC-MS 1
LC-MS: Rt = 4.53 min; MS m/z $[M+H-Boc]^+$ 327.1/329.1, m/z $[M-H]^-$ 425.2/427.2; UPLC-MS 2
^{1}H NMR (400 MHz, DMSO-d_6) δ 13.27 (s, 1H), 3.91 (m, 2H), 3.31 (m, 2H), 2.88 (m, 2H), 2.75 (m, 2H), 2.61 (m, 2H), 1.42 (s, 9H), 1.17 (t, J = 7.4 Hz, 3H)

Sodium salt formation:
The compound was suspended in tert-butanol. NaOH 0.1M (1 eq) was added. The mixture was stirred / sonicated at RT. If the suspension turned into a clear solution it was lyophilized. If the suspension was still turbid, water was added, and the resulting solution was lyophilized. If no change happened, NaOH 0.1M up to 2 eq in total was added until a clear solution was observed, which was then lyophilized. If the NMR of the resulting solid still contained tert-butanol, the solid was dissolved in a small amount of water and lyophilized again. The final sodium salts were obtained as colorless powders. The amorphous state was confirmed by XRPD.

Supplementary Table 1. Proliferation inhibition of HRO761 for a panel of cell lines

Cell line	4 day assay GI_{50} [nM]	CFA 10-14 day assay GI_{50} [nM]	Doubling time [h]
RL952	144	16	40
HEC6	365	16	44
SNU407	440	25	35
SNUC2A	542	25	47
LS411N	257	30	22
SW48	58	31	28
SNUC4	145	35	26
JHUEM1	946	35	38
CL34	231	39	37
TGBC11TKB	178	44	70
Ishikawa	228	51	21
HCT116	143	59	18
HEC265	10000	61	35
CW2	112	68	20
LOVO	1245	73	29
KM12	1382	100	26
HEC108	1462	104	33
HCT116-WRN-C727A cl.2	687	121	22
LS180	3497	131	29
HEC59	10000	133	35
OVK18	10000	150	30
RKO	2906	243	24
IM95	2006	255	32
HEC151	10000	303	23
SNUC5	7236	418	26
LIM2405	1533	452	24
IGROV1	2755	473	33
JHUEM2	10000	929	28
TOV21G	10000	1311	32
SNU520	10000	1743	60
2313287	10000	2077	41
OC314	10000	4626	27
SNGM	10000	7293	36
DLD-1 (HCT15)	10000	10000	20
CAL33	10000	10000	25
EN	10000	10000	44
HEC1A	10000	10000	31
HEC1B	10000	10000	35
HEC251	10000	10000	51
LS513	10000	10000	33
NUGC3	10000	10000	25
RKO-WRN-C727S/C727S cl.1	10000	10000	23
SNU1	10000	10000	25

Supplementary Table 2. Antibodies used for WRN degradation ELISA.

	Primary antibody	Secondary antibody	Tertiary antibody
total WRN	Rabbit anti WRN (Novus #NB100-471) 1/250	mouse anti WRN (Cell Signaling Technology #4666) 1/250	Goat anti mouse-HRP antibody (Cell Signaling Technology #7076) 1/1000

Supplementary Table 3. TaqMan probes used for RTqPCR.

Target	Description
CDKN1A p21	Hs.PT.40874346g
GDF15	Hs.PT.58.40089589
KIF20A	Hs.PT.58.19381842
CENPA	Hs.PT.58.45530067
BTG2	Hs.PT.58.4596312
actin Beta	Hs.PT.39a.22214847

Supplementary Table 4. Antibodies used for immunohistochemical staining.

Antibody	Clone	Brand, catalog number	Used dilution	Epitope Retrieval
Anti WRN (EPR6392) Rabbit mAb	EPR6392	Abcam, ab124673	1/3000	ER2
pCHK2 (Thr68) Rabbit mAb	20E3	CST, 9718	1/50	ER1

Supplementary Table 5. Antibodies coupled with specific Opal dyes used for tumor content (PanCK) and tumor cells proliferation (Ki67) staining.

Order	Rabbit antibody	Clone	Brand, catalog number	Opal pairing [nm]
1	Phospho-Histone H2A.X (Ser139)	20E3	CST, 9718	570
2	pCHK2 (Thr68)	C13C1	CST, 2197	520
3	p21 Waf1/Cip1	12D1	CST, 2947	620
4	Pan Cytokeratin	KRT/1877R	Abcam, ab234297	690
5	Ki67	SP6	Neomarkers, RM9106	780

Supplementary Table 6. Cell lines name, growth media used and cell line origin.

Cell line	Medium composition and reference
JHUEM1 [1]	DMEM / HAM's F12 (BioConcept 1-26F08-I), 15% fetal bovine serum (Corning #35-15-CV), 2 mM Glutamine (BioConcept #5-10K00-H), 1 mM sodium pyruvate (BioConcept #5-60F00-H), 1x non-essential amino acids (BioConcept #5-13K00-H), 10 mM HEPES (BioConcept #5-31F00-H), 1% Penicillin 100 IU.ml-1 / Streptomycin 100 µg.ml-1 (BioConcept #4-01F00-H)
CAL33 [2], CL34 [2]	DMEM High glucose (BioConcept 1-26F01-I), 10% fetal bovine serum (Corning #35-15-CV), 4 mM Glutamine (BioConcept #5-10K00-H), 1 mM sodium pyruvate (BioConcept #5-60F00-H), 1x non-essential amino acids (BioConcept #5-13K00-H), 1% Penicillin 100 IU.ml-1 / Streptomycin 100 µg.ml-1 (BioConcept #4-01F00-H)
IM95 [3]	DMEM High glucose (BioConcept 1-26F01-I), 10% fetal bovine serum (Corning #35-15-CV), 4 mM Glutamine (BioConcept #5-10K00-H), 1 mM sodium pyruvate (BioConcept #5-60F00-H), 1x non-essential amino acids (BioConcept #5-13K00-H), 1% Penicillin 100 IU.ml-1 / Streptomycin 100 µg.ml-1 (BioConcept #4-01F00-H), 10µg/ml human insulin (Sigma #I9278)
HEC108 [4], HEC6 [3], ISHIKAWA [5], LS180 [6], OVK18 [1], RKO [6]	EMEM (BioConcept 1-31S01-I), 10% fetal bovine serum (Corning #35-15-CV), 4 mM Glutamine (BioConcept #5-10K00-H), 1 mM sodium pyruvate (BioConcept #5-60F00-H), 1x non-essential amino acids (BioConcept #5-13K00-H), 1% Penicillin 100 IU.ml-1 / Streptomycin 100 µg.ml-1 (BioConcept #4-01F00-H)
HEC151 [3], HEC265 [3], HEC59 [3]	EMEM (BioConcept 1-31S01-I), 15% fetal bovine serum (Corning #35-15-CV), 4 mM Glutamine (BioConcept #5-10K00-H), 1 mM sodium pyruvate (BioConcept #5-60F00-H), 1x non-essential amino acids (BioConcept #5-13K00-H), 1% Penicillin 100 IU.ml-1 / Streptomycin 100 µg.ml-1 (BioConcept #4-01F00-H)
SNGM [3]	HAM's F12 (BioConcept #1-14F01-I), 20% fetal bovine serum (Corning #35-15-CV), 4 mM Glutamine (BioConcept #5-10K00-H), 1 mM sodium pyruvate (BioConcept #5-60F00-H), 1% Penicillin 100 IU.ml-1 / Streptomycin 100 µg.ml-1 (BioConcept #4-01F00-H)
HCT116 [6], HCT116-WRN-C727A cl.2 [7], HEC1A [6], HT29 [6]	McCoys 5A (BioConcept 1-18F01-I), 10% fetal bovine serum (Corning #35-15-CV), 2 mM Glutamine (BioConcept #5-10K00-H), 1% Penicillin 100 IU.ml-1 / Streptomycin 100 µg.ml-1 (BioConcept #4-01F00-H)
LOVO [3]	RPMI 1640 (BioConcept 1-41F01-I) / Ham'sF12K (BioConcept 1-14S50-I), 2% fetal bovine serum (Corning #35-15-CV), 2 mM Glutamine (BioConcept #5-10K00-H), 1 mM sodium pyruvate (BioConcept #5-60F00-H), 10 mM HEPES (BioConcept #5-31F00-H), 1% Penicillin 100 IU.ml-1 / Streptomycin 100 µg.ml-1 (BioConcept #4-01F00-H)
MFE319 [2]	RPMI 1640 (BioConcept 1-41F01-I) / MEM (Invitrogen Catalog # 12571-063), 10% fetal bovine serum (Corning #35-15-CV), 2 mM Glutamine (BioConcept #5-10K00-H), 1 mM sodium pyruvate (BioConcept #5-60F00-H), 10 mM HEPES (BioConcept #5-31F00-H), 1% Penicillin 100 IU.ml-1 / Streptomycin 100 µg.ml-1 (BioConcept #4-01F00-H)
MFE296 [2]	RPMI 1640 (BioConcept 1-41F01-I) / MEM (Invitrogen Catalog # 12571-063), 20% fetal bovine serum (Corning #35-15-CV), 2 mM Glutamine (BioConcept #5-10K00-H), 1 mM sodium pyruvate (BioConcept #5-60F00-H), 10 mM HEPES (BioConcept #5-31F00-H), 1% Penicillin 100 IU.ml-1 / Streptomycin 100 µg.ml-1 (BioConcept #4-01F00-H)
2313287 [2], COLO684 [1], CW2 [2], DLD-1 (HCT15) [6], KM12 [4], LIM2405 [9], LNCAP cl.FGC [6], LS411N [6], LS513 [6], MKN7 [4], NUGC3 [3], SNU1 [6], SNU407 [10], SNUC2A [6], SNUC4 [10], SNUC5 [10], SW48 [4], TCCPAN2 [3], TOV21G [6]	RPMI 1640 (BioConcept 1-41F01-I), 10% fetal bovine serum (Corning #35-15-CV), 4 mM Glutamine (BioConcept #5-10K00-H), 1 mM sodium pyruvate (BioConcept #5-60F00-H), 10 mM HEPES (BioConcept #5-31F00-H), 1% Penicillin 100 IU.ml-1 / Streptomycin 100 µg.ml-1 (BioConcept #4-01F00-H)

Footnotes: [1] Riken, [2] DSMZ, [3] HSRRB, [4] CCMC, [5] Sigma, [6] ATCC, [7] lab Cortes-Cross, [8] ECACC, [9] Cell bank Australia, [10] KCLB
