# Supplementary material for: Discovery of WRN inhibitor HRO761 with synthetic lethality in MSI cancers
Source: Nature. 2024 Apr 24;629(8011):443–9. doi: 10.1038/s41586-024-07350-y (PMC11078746; doi:10.1038/s41586-024-07350-y)
Supplement: Supplementary file 2 — Reporting Summary [file 41586_2024_7350_MOESM2_ESM.pdf]

nature portfolio

Corresponding author(s): Marta Cortes-Cros

Last updated by author(s): February 8, 2024

Reporting Summary

Nature Portfolio wishes to improve the reproducibility of the work that we publish. This form provides structure for consistency and transparency in reporting. For further information on Nature Portfolio policies, see our Editorial Policies and the Editorial Policy Checklist.

Please do not complete any field with "not applicable" or n/a. Refer to the help text for what text to use if an item is not relevant to your study.
For final submission: please carefully check your responses for accuracy; you will not be able to make changes later.

Statistics

For all statistical analyses, confirm that the following items are present in the figure legend, table legend, main text, or Methods section.

n/a	Confirmed	
☐	☒	The exact sample size (*n*) for each experimental group/condition, given as a discrete number and unit of measurement
☐	☒	A statement on whether measurements were taken from distinct samples or whether the same sample was measured repeatedly
☐	☒	The statistical test(s) used AND whether they are one- or two-sided<br>*Only common tests should be described solely by name; describe more complex techniques in the Methods section.*
☐	☒	A description of all covariates tested
☐	☒	A description of any assumptions or corrections, such as tests of normality and adjustment for multiple comparisons
☐	☒	A full description of the statistical parameters including central tendency (e.g. means) or other basic estimates (e.g. regression coefficient) AND variation (e.g. standard deviation) or associated estimates of uncertainty (e.g. confidence intervals)
☐	☒	For null hypothesis testing, the test statistic (e.g. *F*, *t*, *r*) with confidence intervals, effect sizes, degrees of freedom and *P* value noted<br>*Give P values as exact values whenever suitable.*
☒	☐	For Bayesian analysis, information on the choice of priors and Markov chain Monte Carlo settings
☒	☐	For hierarchical and complex designs, identification of the appropriate level for tests and full reporting of outcomes
☒	☐	Estimates of effect sizes (e.g. Cohen's *d*, Pearson's *r*), indicating how they were calculated

Our web collection on statistics for biologists contains articles on many of the points above.

Software and code

Policy information about availability of computer code

Data collection

PX-ray diffraction data were collected at the Swiss Light Source using Beamline X10SA.
Luminescence output for biochemical assays was recorded using The PHERAstar FSX Microplate Reader (BMG LabTech).
Proteomics data was collected on the Orbitrap Fusion Lumos LCMS system (Thermo Scientific)
Luminescence quantification for viability assays was recorded using a MPLEX multi-mode plate-reader (TECAN) or a Synergy HT plate-reader.
Long term proliferation assays data images were acquired using Incucyte®S3 live-cell analysis instrument (Sartorius).
RTqPCR data was acquired on the 7900HT Device (Applied Biosystem)
RNASeq data was collected using either the HiSeq 4000 or the NovaSeq 6000 instruments (Illumina).
Immunofluoresce images were captured and analyzed using a PerkinElmer Opera Phenix imager.
FACS data were collected using the on Cytoflex flow cytometer (Beckman Coulter).
Immunoblot images were acquired using a fusion FX device (Witec).
Immunohistochemical analysis and images were acquired using a PhenoImager® HT multispectral imaging system

Data analysis

The data were analyzed using commercially available software as stated in the Methods section, including autoPROC/STARANISO, PHASER , Proteome Discoverer v.2.4, Graphpad Prism v10.1.2, SDS2.4.1, Harmony v.4.9, CytExpert v.2.4, Fusion FX (EvolutionCap) v. 17.0, R v. 4.2.1, DEseq2 R package v.1.36.0, fgsea R package v.1.25.1, Pisces v.0.1.3.1.

For manuscripts utilizing custom algorithms or software that are central to the research but not yet described in published literature, software must be made available to editors and reviewers. We strongly encourage code deposition in a community repository (e.g. GitHub). See the Nature Portfolio guidelines for submitting code & software for further information.

Data

Policy information about availability of data

All manuscripts must include a data availability statement. This statement should provide the following information, where applicable:

- Accession codes, unique identifiers, or web links for publicly available datasets
- A description of any restrictions on data availability
- For clinical datasets or third party data, please ensure that the statement adheres to our policy

nature portfolio

Corresponding author(s): Marta Cortes-Cros

Last updated by author(s): February 8, 2024

Data

Policy information about availability of data

All manuscripts must include a data availability statement. This statement should provide the following information, where applicable:

- Accession codes, unique identifiers, or web links for publicly available datasets
- A description of any restrictions on data availability
- For clinical datasets or third party data, please ensure that the statement adheres to our policy

Atomic coordinates and structure factors for the co-crystal structures have been deposited in the protein data bank with accession codes 8pfp (WRN.ATPγS), 8pfl (WRN.4) and 8pfo (WRN.2).
Synthesis of compounds 2-5, including 1H and 13C NMR data in the "Supplementary Information" section.
All raw sequencing data from this study have been deposited to SRA under BioProject IDs: PRJNA995921 and PRJNA995923.
The mass spectrometry proteomics data have been deposited to the ProteomeXchange Consortium via the PRIDE 56 partner repository with the dataset identifier PXD044202
Atomic coordinates and structure factors for the co-crystal structures have been deposited in the Protein Data bank with accession codes 8pfp (WRN.ATPγS), 8pfl (WRN.3) and 8pfo (WRN.HRO761).
Synthesis of compounds 2-6, including 1H and 13C NMR data are described in the "Supplementary Information" section.
All raw sequencing data from this study have been deposited to SRA under bioproject ids PRJNA995921 and PRJNA995923 at the following location:
- https://www.ncbi.nlm.nih.gov/bioproject/PRJNA995921
- https://www.ncbi.nlm.nih.gov/bioproject/PRJNA995923.

The mass spectrometry proteomics data have been deposited to the ProteomeXchange Consortium via the PRIDE 56 partner repository with the dataset identifier PXD044202 (Project Webpage: http://www.ebi.ac.uk/pride/archive/projects/PXD044202; FTP Download: https://ftp.pride.ebi.ac.uk/pride/data/archive/2024/01/PXD044202).

Research involving human participants, their data, or biological material

Policy information about studies with human participants or human data. See also policy information about sex, gender (identity/presentation), and sexual orientation and race, ethnicity and racism.

Reporting on sex and gender	not applicable
Reporting on race, ethnicity, or other socially relevant groupings	not applicable
Population characteristics	not applicable
Recruitment	not applicable
Ethics oversight	not applicable

Note that full information on the approval of the study protocol must also be provided in the manuscript.

Field-specific reporting

Please select the one below that is the best fit for your research. If you are not sure, read the appropriate sections before making your selection.

[x] Life sciences   [ ] Behavioural & social sciences   [ ] Ecological, evolutionary & environmental sciences

For a reference copy of the document with all sections, see nature.com/documents/nr-reporting-summary-flat.pdf

Life sciences study design

All studies must disclose on these points even when the disclosure is negative.

Sample size

For efficacy experiment as single agent or in combination, mice were randomized into groups (n=5-7) for a mean tumor size of 150-200 mm3. Sample size depends on the tumor cell line being used. Log10 transformation may be used to stabilize the variance as variability in tumor volume measurements often increases with tumor volume. In the least variable cell lines (e.g. SW48) group sizes of 4-6 are sufficient to provide 80% power of detecting a 30% decrease in tumor growth between a compound treated and vehicle group in a one-sided statistical test conducted at the 5% significance level.
For PD experiment in SW48 tumor-bearing mice, animals were randomized (n=3) and tumor samples were collected at 0, 1, 4, 8 and 24h on the first day and then collected 4h post last treatment on day 3, 8, 10, 14 (including a 24h time point), 17 and 21. The lowest number for a PK/PD experiment for a realistic statistical analysis is 3 animals per time-point, and ideally, at least 5 time points are needed for accuracy in determining, for example, AUC (area under the curve) and the rate of elimination from blood.

Data exclusions	At the time of the randomization, mice with tumors too small (<50 mm3) or too large (>300 mm3) were excluded. No mice were excluded from any analysis.
Replication	Each in vivo experiment in SW48 model presented in the paper was repeated independently at least twice.
Randomization	Tumor-bearing mice were randomized between vehicle and treatment groups with an INDIGO software.
Blinding	The investigators were not blinded during data collection and analysis.

Behavioural & social sciences study design

All studies must disclose on these points even when the disclosure is negative.

Study description	
Research sample	
Sampling strategy	
Data collection	
Timing	
Data exclusions	
Non-participation	
Randomization	

Ecological, evolutionary & environmental sciences study design

All studies must disclose on these points even when the disclosure is negative.

Study description	
Research sample	
Sampling strategy	
Data collection	
Timing and spatial scale	
Data exclusions	
Reproducibility	
Randomization	
Blinding	

Did the study involve field work? ☐ Yes ☐ No

Field work, collection and transport

Field conditions	
Location	
Access & import/export	
Disturbance	

nature portfolio | reporting summary

Reporting for specific materials, systems and methods

We require information from authors about some types of materials, experimental systems and methods used in many studies. Here, indicate whether each material, system or method listed is relevant to your study. If you are not sure if a list item applies to your research, read the appropriate section before selecting a response.

Materials & experimental systems

n/a	Involved in the study	
☐	☒	Antibodies
☐	☒	Eukaryotic cell lines
☒	☐	Palaeontology and archaeology
☐	☒	Animals and other organisms
☒	☐	Clinical data
☒	☐	Dual use research of concern
☒	☐	Plants

Methods

n/a	Involved in the study	
☒	☐	ChIP-seq
☐	☒	Flow cytometry
☒	☐	MRI-based neuroimaging

Antibodies

Antibodies used

Antibody name (Company, Catalog #, dilution if appropriate):

Rabbit anti WRN (Novus, #NB100-471, 1:250)
Mouse anti WRN (Cell Signaling Technology, #4666, 1:250)
Goat anti mouse-HRP antibody (Cell Signaling Technology, #7076, 1:1000)
Anti-ATM (Cell Signaling Technology, #2873, 1:1000),
Anti-Phosphor-ATM ser1981 (Abcam, #ab81292, 1:1000),
Anti-CHK2 (Cell Signaling Technology, #3440, 1:1000),
Anti-Phospho-CHK2 thr68 (Cell Signaling Technology, #2197, 1:1000),
Anti-p53 (Calbiochem, #OP43, 1:2000),
Anti-p21 (Cell Signaling Technology, #2947, 1:2000),
Anti-WRN (Millipor,e #MABD34, 1:1000),
Anti-Actin (Chemicon, #MAB1501, 1:10000),
Anti-Phospho-H2AX ser139 (Cell Signaling Technology, #9718, 1:1000),
Anti-Phospho-CHK1 ser345 (Cell Signaling Technology, #2348, 1:1000),
Anti-Phospho-KAP1 ser824 (Cell Signaling Technology, #4127, 1:1000),
Anti-Phospho-ATR thr1989 (Cell Signaling Technology, #30632, 1:1000),
Anti-MDM2 (Calbiochem, #OP46, 1:1000),
Anti-Tubulin (Sigma, #T9026, 1:10000),
Anti-PUMA (Cell Signaling Technology, #12450, 1:1000),
Goat anti-rabbit (Cell Signaling Technology, #7074, 1:5000),
Goat anti-mouse (Cell Signaling Technology, #7076, 1:5000).
Anti gH2AX (Millipore, #05-636)
Goat anti mouse Alexa Fluor 488-conjugated antibody (Invitrogen, #A11001)
Anti-phospho-Histone 3 ser10 conjugated to PE (Cell Signalling Technology,#5764)
Anti WRN (EPR6392) (Abcam, #ab124673)
Anti-p21 Waf1/Cip1 (Cell Signaling Technology, # 2947)
Anti-Pan Cytokeratin KRT/1877R (Abcam, #ab234297)
Anti-Ki67 SP6 (Neomarkers, #RM9106).
Anti-SP1 (Cell Signaling Technology #9389, 1:1000)
Anti-phospho-Histone H3 (Cell Signaling Technology #9706, 1:1000)
Anti-Cyclin E (Upstate #06-134, 1:1000)
Anti- Cyclin A (Sigma #C4710, 1:1000)
Anti- Cyclin B1 (Cell Signaling Technology #°55506, 1:1000)

Validation

Antibodies critical for novel conclusions were validated by elimination of signals upon KD experiments and/or by induction of signal in functional assays and/or by use of cell lines selected to have very high and very low expression at the RNA level. All antibodies were used in the system under study (assay and species) according to the profile of manufacturer as listed above.

Eukaryotic cell lines

Policy information about cell lines and Sex and Gender in Research

Cell line source(s) — All cell lines are part of the original Cancer Cell Line Encyclopedia (CCLE). Cell line media and origin are described in SI Table 5. The HCT116 TP53-/- and the RKO C727S KI cells were supplied by Horizon Discovery.

Authentication — All cell lines were fingerprinted by SNP arrays except for the HCT116 TP53-/- cells were bought from Horizon Discovery (Cat# D 104-001) and RKO C727S KI which were generated by Horizon Discovery.

Mycoplasma contamination — Cell lines were tested negative for mycoplasma.

Commonly misidentified lines (See ICLAC register) — Identity of the cells were confirmed by SNP arrays except where specified.

Palaeontology and Archaeology

Specimen provenance

Specimen deposition

Dating methods

☐ Tick this box to confirm that the raw and calibrated dates are available in the paper or in Supplementary Information.

Ethics oversight

Note that full information on the approval of the study protocol must also be provided in the manuscript.

Animals and other research organisms

Policy information about studies involving animals; ARRIVE guidelines recommended for reporting animal research, and Sex and Gender in Research

Laboratory animals — Details on the mice used in this study have been included in the Methods section of this manuscript. 6–7-week-old female athymic nude (Crl:NU(NCr)-Foxn1nu) or SCID-BEIGE (CB17.Cg-PrkdcscidLystbg-J/Crl) mice were purchased from Charles River. All animals had access to food and water ad libitum. They were housed in a specific pathogen-free facility in IVC racks (NexGen Edge mouse 500, Allentown) with a 12 h light/12 h dark cycle.

Wild animals — This study did not not involve wild animals.

Reporting on sex — All mice were female.

Field-collected samples — This study did not involve samples collected from the field.

Ethics oversight — All animal studies were conducted in accordance with ethics and procedures covered by permit BS-2275 and BS-1975 issued and approved by the Kantonales Veterinäramt Basel-Stadt and in strict adherence to guidelines of the Eidgenössisches Tierschutzgesetz and the Eidgenössische Tierschutzverordnung, Switzerland. All animal studies were approved by the internal ethics committee.

Note that full information on the approval of the study protocol must also be provided in the manuscript.

Clinical data

Policy information about clinical studies

All manuscripts should comply with the ICMJE guidelines for publication of clinical research and a completed CONSORT checklist must be included with all submissions.

Clinical trial registration

Study protocol

Data collection

Outcomes

Dual use research of concern

Policy information about dual use research of concern

Hazards

Could the accidental, deliberate or reckless misuse of agents or technologies generated in the work, or the application of information presented in the manuscript, pose a threat to:

No	Yes	
X	☐	Public health
X	☐	National security
X	☐	Crops and/or livestock
X	☐	Ecosystems
X	☐	Any other significant area

Experiments of concern

Does the work involve any of these experiments of concern:

No	Yes	
X	☐	Demonstrate how to render a vaccine ineffective
X	☐	Confer resistance to therapeutically useful antibiotics or antiviral agents
X	☐	Enhance the virulence of a pathogen or render a nonpathogen virulent
X	☐	Increase transmissibility of a pathogen
X	☐	Alter the host range of a pathogen
X	☐	Enable evasion of diagnostic/detection modalities
X	☐	Enable the weaponization of a biological agent or toxin
X	☐	Any other potentially harmful combination of experiments and agents

Plants

Seed stocks

Novel plant genotypes

Authentication

ChIP-seq

Data deposition

☐ Confirm that both raw and final processed data have been deposited in a public database such as GEO.

☐ Confirm that you have deposited or provided access to graph files (e.g. BED files) for the called peaks.

Data access links
May remain private before publication.

Files in database submission

Genome browser session
(e.g. UCSC)

Methodology

Replicates

Sequencing depth

Antibodies

Peak calling parameters

Data quality

Software

Flow Cytometry

Plots

Confirm that:

- [x] The axis labels state the marker and fluorochrome used (e.g. CD4-FITC).
- [x] The axis scales are clearly visible. Include numbers along axes only for bottom left plot of group (a 'group' is an analysis of identical markers).
- [x] All plots are contour plots with outliers or pseudocolor plots.
- [x] A numerical value for number of cells or percentage (with statistics) is provided.

Methodology

Sample preparation	2×10e6 HCT116 cells were treated with either DMSO or HRO761 at 10 µM for 24 hours. Cells were harvested by trypsin and washed once with PBS. Cells were pelleted and fixed in 100 µL 4% formaldehyde (Sigma ref.47608) for 15 min at RT. Cells were washed by centrifugation with excess of 1X PBS. Pellets were resuspended in 100 µL 1X PBS, then 900 µL of ice-cold methanol (Sigma ref.34860) was added drop-by-drop while vortexing. Cells were incubated 10 min on ice. Cells were washed by centrifugation with excess of 1X PBS, pellets were resuspended in 100 µL antibody phospho-Histone 3 ser10 conjugated to PE (Cell Signalling Technology ref.5764) diluted 1/50 in 0.5% BSA PBS buffer and incubated for 1h at RT protected from the light. After 2 washes by centrifugation with excess of 1X PBS, cells were stained with 3 µM final concentration of DAPI solution (ThermoScientific ref.62248) for 1 to 5 min protected from the light. Cells were washed 2 times by centrifugation with excess of 1X PBS.
Instrument	Beckman Coulter Cytoflex S flow cytometer
Software	Data were collected and analyzed with CytExpert 2.4 software.
Cell population abundance	10,000 cells from each sample were analyzed from each experiment
Gating strategy	As described in the Methods section, debris and dead cells were excluded based on forward scatter-area (FSC-A) and side scatter-area (SSC-A) profiles. Subsequently, singlets were identified based on FSC-A and forward scatter-height (FSC-H) profiles. These singlets were then analyzed for DAPI (DNA content) and PE (phospho-Histone 3 ser10) staining intensities. Data were analyzed to show the percentage of nuclei in the G1, S, and G2/M phases using DAPI channel and the percentage of cells specifically in G2 or M phase using PE channel.

- [x] Tick this box to confirm that a figure exemplifying the gating strategy is provided in the Supplementary Information.

Magnetic resonance imaging

Experimental design

Design type

Design specifications

Behavioral performance measures

Imaging type(s)

Field strength

Sequence & imaging parameters

Area of acquisition

Diffusion MRI ☐ Used ☐ Not used

Preprocessing

Preprocessing software

Normalization

Normalization template

Noise and artifact removal

Volume censoring

Statistical modeling & inference

Model type and settings

Effect(s) tested

Specify type of analysis: ☐ Whole brain ☐ ROI-based ☐ Both

Statistic type for inference

(See Eklund et al. 2016)

Correction

Models & analysis

n/a	Involved in the study
☐	☐ Functional and/or effective connectivity
☐	☐ Graph analysis
☐	☐ Multivariate modeling or predictive analysis

Functional and/or effective connectivity

Graph analysis

Multivariate modeling and predictive analysis

This checklist template is licensed under a Creative Commons Attribution 4.0 International License, which permits use, sharing, adaptation, distribution and reproduction in any medium or format, as long as you give appropriate credit to the original author(s) and the source, provide a link to the Creative Commons license, and indicate if changes were made. The images or other third party material in this article are included in the article's Creative Commons license, unless indicated otherwise in a credit line to the material. If material is not included in the article's Creative Commons license and your intended use is not permitted by statutory regulation or exceeds the permitted use, you will need to obtain permission directly from the copyright holder. To view a copy of this license, visit http://creativecommons.org/licenses/by/4.0/

April 2023

#
