# Supplementary material for: Discovery of WRN inhibitor HRO761 with synthetic lethality in MSI cancers
Source: Nature. 2024 Apr 24;629(8011):443–9. doi: 10.1038/s41586-024-07350-y (PMC11078746; doi:10.1038/s41586-024-07350-y)
Supplement: Supplementary file 3 — Peer Review File [file 41586_2024_7350_MOESM3_ESM.pdf]

# Peer Review File

**Manuscript Title:** Discovery of allosteric WRN inhibitor HRO761 demonstrating synthetic lethality in MSI cancers

## Editorial Notes:

**Redactions – Third Party Material**

Parts of this Peer Review File have been redacted as indicated to remove third-party material.

## Reviewer Comments & Author Rebuttals

**Reviewer Reports on the Initial Version:**

Referees' comments:

Referee #1 (Remarks to the Author):

In this paper, Ferretti et al report the discovery of the first-in-class clinical small molecule inhibitor of WRN, HRO761. The authors show that HRO761 binds non-covalently to an allosteric site on WRN, resulting in inhibited WRN function in in vitro biochemical assays. When applied to cells, HRO761 selectively impair the viability of MSI but not MSS cells. The mechanism is complex, since HRO761 treatment also led to proteasome-mediated degradation of WRN. The authors further show that loss of WRN induced by HRO761 causes DNA damage accumulation, checkpoint arrest and death of susceptible MSI cells, recapitulating effects that have previously been reported when WRN was depleted by other means. Finally, they demonstrate that the efficacy of HRO761 is not dependent on p53, is extended to in vivo situations (with CDX models) and that low doses of HRO761 can be combined with irinotecan to achieve synergistic activity. Overall, this paper introduces a novel compound that should be of broad interest to the public and the medical field. However, apart from that, this study does not offer any real new insight on the biological basis of WRN dependency beyond what has already been described. Furthermore, the mechanism by which HRO761 inhibits WRN function in vivo has not been clearly established, because it was shown that HRO761 is cytotoxic only in those cell types where it induces WRN degradation, even though in vitro HRO761 was shown to inhibit WRN activity. Due to its lack of additional clear and novel data with regards to WRN's function in MSI cells in vitro and in vivo, this manuscript, in its current form, may be better suited to be published in a journal with a more directly clinical focus. Additionally, while it is appreciated that there is likely to a race to report the first clinical WRN inhibitor, the drafting of this report is somewhat rushed, not least due to a general lack of detailed description in many figure legends and a degree of sloppiness in their presentation.

Major points

If DNA damage initiates the degradation of WRN, are MSI cells far more sensitive to classic alkylating chemotherapy agents, or topoisomerase inhibitors, compared to MSS cells? If not, do these agents not lead to WRN degradation like they cite? A lot of their mechanism rests on this assumption being true.

Related to this, in cells treated with Compound 4 and ATMi, the authors show that WRN is no longer degraded (Extended Data Fig 3C). This suggests that DNA damage initiated by loss of WRN ultimately leads to the protein's degradation. Does cotreatment of cells with HRO761 and ATMi lead to better

cell survival compared to treatment with HRO761 on its own? If not, then it seems that the Compound's effect is primarily via more short-term WRN inhibition and the degradation phenotype observed is then a secondary effect that is not driving cell death. The authors should be aware of a recent paper in which rapid WRN degradation does lead to synthetic lethality in MSI (https://www.biorxiv.org/content/10.1101/2023.07.28.550976v1).

Fig 2b: Are there 8 MSS cell lines plotted, as indicated in the legend? It looks like 2 cell lines in the plot.

Fig 2, panel c. The authors used the Protein Stabilization assay to show that binding of HRO761 to the target protein is comparable in MSI and MSS cells. This, together with genetic dependency uncovered by DRIVE, led the authors to conclude that HRO761 acts by selectively inhibiting WRN. Although certainly suggestive, to definitively show that this is the case the authors should test HRO761 in WRN-deficient cells complemented with exogenous WRN. Additionally, what are the units for PS50 on the y-axis?

Fig 2, panel e. Why do HRO761-treated MSI cells show diminished levels of WRN, when based on the protein stabilization assay HRO761 appeared to stabilize WRN? Also, in panel e it was shown that p21 was significantly induced in four MSI cell lines after HRO761 while in Extended Fig 1e, RKO cells depleted for WRN showed no induction of p21 (at least based on the zoomed out IHC image). At first glance, this appears to be internally inconsistent.

Fig 2, panel F (and in text). What method are they using to measure protein levels? How are they plotting three cell lines in one Volcano plot (explain if they are taking the average value for each protein). This experiment needs to be better explained in the figure legend. What is on the x-axis, what is on the y-axis?

Fig 2, panel g. I assume that the data is presented as a heatmap to show that hypersensitivity to HRO761 in CFA correlates with reduced WRN protein level. However, the way the data is presented is not self-explanatory and the legend is somewhat confusing. What is Max/Average/Min and what does it refer to?

Fig 2, panel h and Extended Fig 4. What is the rationale for using analogs of HRO761 rather than HRO761 itself in these experiments? In panel h, there is no real point in testing DLD1 WRN-KO cells, since DLD1 is known to be refractory to WRN inhibition anyway.

Extended Fig 3. Panel a, the figure legend states that cells were treated with camptothecin. However, the plot indicated that cells were treated with Eto, a commonly used abbreviation for etoposide. In panels b and c, it was shown that WRN degradation is mediated by ATM and the proteasome. It would be nice if the authors could test whether proteasome/ATM inhibition could partially rescue MSI cells from WRN inhibition. This may have important therapeutic implications since proteasome/ATM inhibitors are being developed/used as anti-cancer therapies.

Extended Fig 4. Panel a, it was stated that mice were treated once and sampled 4 h post last treatment. This statement is a bit unclear, does it mean mice were treated for 4 h and immediately

sacrificed? That seems unlikely since many of the effects in cell lines took hours to days to manifest. What are the tumor cells used in panel a-c? In panel d, is 4% Reg short for 4% regression? At what dose does the WRN inhibitor become toxic?

Fig 3 and Extended Fig 5. The authors reported that HRO761 induces ATM activation within 1 h of treatment, while WRN degradation began after 8 h (extended fig 5). They also reported that the treatment induced G2 arrest by 24 h in Fig 3d, but earlier time points have not been investigated. Is it possible that G2 arrest caused/promoted WRN degradation? As it stands, although likely, it is not completely certain that HRO761 binding itself induces WRN degradation. Since treatment with CPT also was reported to degrade WRN (which should be confirmed), I wonder if WRN degradation simply reflects a general feature of WRN protein instability in G2, especially if the arrest is prolonged? The authors should test another treatment condition that induces G2 arrest in their cells and assess whether WRN degradation also occurs in that context. It is not sufficient to say that WRN degradation occurs only in WRN-sensitive cells because in the insensitive lines HRO761 treatment also does not lead to G2 arrest. In Fig 3a, why was HRO761 labeled as RJT779; is that another name for the compound? In Extended Fig 5, it seems that in addition to WRN, treatment with HRO761 also induced time-dependent degradation of CHK1 (and CHK2). Thus, I'm wondering if (1) this property is also shared with CPT treatment, (2) what might be the cause of this phenomenon (hyper-instability of DDR factors in G2) and (3) can lethality really be attributed solely to WRN loss (being as CHK1 is an essential gene)?

Fig. 4, panel a, treatment with HRO761 initially led to tumor regression, but tumor growth appeared to re-start towards the end of the experiment. Is this due to acquisition of resistance mechanisms?

Fig 4C: Can the authors comment on why does KAP1 phosphorylation, a marker of DNA damage, decrease after Day 14 of HRO761 administration, even when pATM levels remain high and WRN levels remain low? The authors write that the DDR remains activated for 21 days, but this doesn't seem to be accurate and should be further discussed (lines 154-156).

Fig. 4, panel e, does the sensitivity of different MSI cell lines to HRO761 correlate with their genetic dependency on WRN? If so, it will be good to show it. If not, what might be the reason for such discrepancy? In CDXs that are less sensitive to HRO761, was WRN degradation induced at a lower level than in highly sensitive CDXs? Related to this issue, Extended data Table 2 shows that the GI50 values of HRO761 in several MSI cell lines known to be highly susceptible to genetic loss of WRN (e.g. KM12, OVK18 and RKO) are much higher than even HCT116 C727A, in which WRN binding by HRO761 should be severely impaired. This is a bit surprising, given that other experiments showed equivalent binding of HRO761 to WRN independently of MS status or cell type. What might be the explanation for the relative lack of HRO761 activity in these MSI lines that are known to be WRN-dependent?

Fig. 4, panel f, the data seems to indicate that after drug washout, MSI cells recover from HRO761. Is HRO761-induced WRN inhibition reversible? This is something that needs to be carefully examined and will have significant impact on dosing.

Extended Fig 7, panel b, are these SW48 cells? If so, why was there no reduction in WRN protein

level observed at 24 h, while WRN was clearly degraded as shown in Fig 4c (60 mg/kg, day 1)? In general, when multiple cell line models are used in the same figure (e.g. SW48, HCT116 etc), it is good practice to label them accordingly (if not in the figures themselves then at least in the figure legends).

Fig. 4 and Extended Fig 7. What is the proposed mechanism for the synergy observed between HRO761 and irinotecan? Based on Extended Fig 7g, it seems that perhaps DNA damage load is increased in cells dually treated with HRO761 and irinotecan. Is this because both HRO761 and irinotecan (inferred from CPT data) could induce partial WRN degradation, and when combined, simply induces more complete WRN degradation? In this regard, it would be interesting to test whether HRO761 synergizes with other drugs frequently used in treating colorectal cancers, such as platinum agents or 5-FU?

Minor comments

Page 1, line 38, abbreviation (MMR) has already been introduced earlier (on line 35)

Page 3, lines 87-88, the authors wrote “To better reflect physiological conditions, we drove our potency optimization with a biochemical WRN ATPase assay with high ATP and low ssDNA concentrations”. Ideally, the authors should include some supplemental info on this assay showing that it indeed measures WRN activity, perhaps benchmarking against one of the previously published experimental WRN inhibitors. Or, if the data is not being published, they should indicate that in the text.

Fig 1. Panel b, there is no explanation of the different colored domains. Is the inhibitor colored in red? Is panel c a zoom-in view of panel b, and if so, what region does it depict? Panels a, c, d, e are not clearly indicated.

Extended Fig 1. Panel a lacks a description of color coding. Panel c, the legend should include a description of the timing of the WB experiment. Panel d, there is no description of statistical significance; for shWRN + WRN K577A, the difference between the black and blue lines (if significant) are much smaller than that shown in the shWRN cohort. Does this indicate that the helicase-dead mutant may still retain some function in vivo? Information should ideally be provided in the legend or as part of the plot on the number of mice used for each condition.

Extended Fig 1. Panel b shows clearly that re-introduction of WT or nuclease-dead WRN into WRN depleted cells rescues lethality. Surprisingly, in panel c, the levels of gH2AX did not appear to decrease in the rescued cells. Moreover, pATM, pChk2, pATR, pChk1 and p53 seemed to be comparable between WRN-depleted cells and cells that were reconstituted with WT WRN, while for cells complemented with E84A mutant, the levels of these DDR markers showed some decrease. These results are a bit confusing, how does exogenous WRN rescue lethality in WRN depleted cells without affecting DNA damage accumulation?

Extended Fig 2. Panels c and d, all of the y-axes have legends that look different in terms of presentation. Part of the legend is obscured in d, left panel.

Extended Fig 7. Panel d, it is not clear whether the “white line” at the bottom is the scale bar.

Finally, it would be advisable to make the fonts bigger for most of the figures. As things stand right now, it’s almost impossible to read certain plots. An additional Extended Figure showing the full blots should also be appended at the end.

Referee #2 (Remarks to the Author):

I commend the authors on a brilliant piece of drug discovery. WRN is an important and challenging target for oncology that has bedeviled much of the pharmaceutical industry. The authors’ patent, which came out earlier in the year, caused quite a stir. Here, they describe in detail their original findings on the first WRN inhibitor (HRO761) to have suitable properties for in vitro cellular pharmacology and in vivo oral administration. With this compound, they have convincingly demonstrated the pharmacological utility and potential for patients with MSI cancer, which was predicted from genetic screens for synthetic lethality ~10 years ago. Their biological findings are most interesting, especially with regards to target degradation. Given the high interest and strong validation for WRN, I believe this manuscript would engage the broader Nature audience and am fully supportive for publication in either Nature or Nature Medicine predicated on addressing the comments below. The authors have omitted some important data and I strongly advise them to include these for acceptance. I hope the authors will take these comments positively and with enthusiasm as they are intended to further improve this excellent manuscript and make for a more clear and rigorous data presentation and broaden the impact. Congratulations on your impressive work and I sincerely hope this will come to fruition in the clinic!

------------------------------------------------------------------------------------------------------------------------------------

Abstract lines 20-21: “… of the first potent and selective WRN helicase inhibitor…”: given that another potent and selective WRN inhibitor (H3B-968) has been published (Parker et al, Biochemistry 2023), please amend the phrase to “…first potent and selective clinical stage WRN helicase inhibitor…”. H3B-968, while potent in biochemical assays, was limited from a properties point of view and not suitable for cellular based assays or in vivo studies. Therefore, keep the emphasis on “clinical stage”, which is sufficiently remarkable and an exciting breakthrough for patients with MSI cancers. With this subtle wording change, you showcase your work without taking away from others.

Clarifying the patient numbers affected by MSI and how many are unresponsive to checkpoint inhibitors may better represent the significance of the author’s findings. For instance, main text line 42: “… a significant fraction of patients …”: please check into the patient numbers to be more precise: “an estimated xx-yy number of patients…” and include appropriate citations.

Main text line 64-65: “extensive hit finding activities…”. It’s not appropriate to title a paper “Discovery of HRO761…” and only have one sentence describing the discovery. Absent any of the underlying discovery data package, the title would need to change to “Characterization of HRO761…”. The pharmaceutical industry is excited about WRN and other challenging helicase targets and the broader Nature audience will be eager to learn from your groundbreaking work and experience. Therefore, in keeping with your title, this section needs to be significantly elaborated. Given the wordcount constraints, this may be provided in the supplementary section. This section should contain a multi-panel figure with the following components:

(a) Screening funnel, including primary HTS assay, secondary assays/counter screens, number of compounds screened (and what libraries whether proprietary or otherwise) and number of hits passing your statistical thresholds at each stage, and please report the % activity of your hit in the primary assays. (How much did the primary hit stand out above the noise?)
(b) Key biophysical data of the primary screening hit. What were the key data that led you to identify this singleton and what gave you confidence that it was a real hit and not a non-specific binder/aggregator. Please include a reasonable subset of supporting DSF / NMR / ITC / SPR / mass spec (HDX / ASMS / etc) data.
(c) What were the key biochemical data that support the MOA? Did the compound show ATP-competition / DNA competition or both (see comment below for lines 69-72)?
(d) What was your first crystal structure from the series that enabled structure-based drug design?
(e) Please provide a table with 5-10 compounds with negative SAR points that validate the interactions observed in the crystal structure. E.g. changing an R-group that disrupts some H-bond interaction reduces the compound potency x-fold.
(f) I would love to see some discussion around how you elaborated your different R-groups, but concede this may be more appropriate in a medchem focused follow up paper. Do we have any assurance from the authors that such a paper is underway? Are they able to share this draft?

Lines 69-72 and extended figure 2. Two things I’d like to see included in your supplement. (1) From my understanding of your mechanistic data, the compound is competitive with respect to ATP and non-competitive with respect to DNA. Your Linweaver Burke plots tell a slightly different story of mixed inhibition for both. I’d suggest adding a table here reporting the IC50 value of the compound under conditions of [ATP] ~ KM and [ATP] ~100X KM. (high and low ATP). Under these conditions, we should see a clear shift in IC50 for an ATP competitive compound. Please also include data showing IC50 of compound with high and low DNA substrate concentrations. For a compound that is non-competitive with respect to DNA, we don’t expect to see a shift in IC50. This may be a more intuitive way to present your MOA data given many readers may not readily recall the meaning of y-intercepts and slopes on a Linweaver Burke plot. (2) To further support your structural interpretation of MOA, I’d like to see additional data for 5 ITC experiments, which should be very easy to perform: First, WRN and titrate compound. Second, WRN and titrate DNA. Third, pre-form the WRN-DNA complex and titrate compound. Fourth, pre-form the WRN-compound complex and titrate DNA. Fifth, WRN and titrate ATP-gammaS (not sure if this last titration is technically feasible given the higher KD). These titration experiments will help prove if your compound binds in the presence of DNA and if DNA binds in the presence of compound. These are very easy experiments and will be highly interesting MOA data to support a non-competitive model. It will also be noteworthy to understand what is the entropic cost of rearranging your D1 and D2 domains upon compound

binding.

Figure 1: The focus of the paper is HR0761. Please include in Figure 1 a summary table with the key target compound profile (TCP) data including MW/cLogD/PSA/solubility/biochemical and cellular activity/selectivity against WRN vs BLM, selectivity in one representative MSI vs one representative MSS cell line, mouse and human liver microsome, mouse and rat or dog or monkey PK including CL, Vss, AUC, and %F. These are the most critical summary data which should be highlighted in the first publication of a clinical stage compound.

Figure 1B: Please indicate in your figure, using a different color, what are the hinge residues and please explain the color scheme in the legend. Is the hinge supposed to be the yellow residues and what are the three mysterious yellow balls?

Figure 1C: This should be one of the most exciting figures in the paper and I would be more than happy to assist the authors if they send me the coordinate file! Otherwise, these are my requests for improvement. (1) With the current color scheme, the compound is camouflaged and difficult to see. The compound is the most important part of the figure, so this should be in a complimentary or brighter color relative to the protein while the protein should be in a more neutral color (not bright blue). (2) The chosen view is unclear. One should never showcase a view where an alpha helix is completely blocking the object you're trying to observe (the compound). I would request the authors to pivot the view ~90 degrees so this helix is more behind/above the compound with the core sitting above Cys727 (this view will also make the rationale for your beautiful target engagement knock-in data for C727A/S at lines 98-99 much more clear). From this view, it will be also easier to highlight how the compound acts like a molecular zipper employing a fascinating H-bond network with several Arg residues from D1/D2 to stabilize this novel conformation! This new view will place the obstructing helix in their current view behind or above their compound and not in front of their compound. Please also cite Parker et al 2023, which proposed the existence of an allosteric pocket around C727, although they were unable to elucidate the impressive conformational changes identified here. Finally, please reorient the 2D-representation of Figure 1A to match the pose in the new view. (3) the authors make some objects semi-transparent and use depth cueing. This makes it hard to tell if something is in front or in back. Use depth cue but avoid semi-transparent rendering. (4) If some residues are unavoidably obstructing a clear view of the compound, crop them out (while minimizing cropping as much as possible) and make a note of this in the legend. (5) far too many interactions are shown, which makes the figure incredibly busy and hard to decipher. Please show only the most critical interactions mentioned in the text including the key Arg-interactions with the compound forming the molecular zipper. (6) None of the residues is labeled. Please label the key Arg residues (711, 854, 857) and Cys 727 in a reasonable and unobtrusive font and color scheme so as not to distract from the compound.

Figure 1 D-E, these can be moved to supplementary to make room for requested TCP table above.

Figure 2A, please label the Y-axis as % growth. This plot seems pixelated. Please make a better figure and not a low-resolution snapshot. I would highly recommend displaying two curves in this figure, one representing MSI cell line and one for MSS. This will nicely illustrate the selectivity of your compound and the window. Please remove the red-dashed lines that show the GI50.

Figure 3F, similar comments to 2A. Please provide meaningful label on Y-axis and make higher quality. The lines are too thin, aesthetically needs improvement. Recommend printing all figures and inspecting their visual quality.

Main text line 79: please show SPR summary table with data illustrating the on/off rates of your lead and final compound. Please include the sensograms and the data fit to a 1:1 kinetic model and heterogeneous ligand model. How do these data fit and is there any circumstantial evidence for two conformational states for WRN?

Extended data figure 1b: You're showing the exonuclease domain as a hexamer. I recommend showing it as a monomer to match your schematic. Otherwise, please explain the hexamer in the legend because this won't be obvious to anyone except the specialists who've read John Tainer's paper.

Extended data figure 1b-d): for the K577A mutant, please mention this is the walker motif and critical for g-phosphate interactions. Would also be nice to relate your genetic functional data to the compound pharmacologic data if possible. In other words, can you tell from the crystal structure does the compound push K577 out of position? Can you show comparison of K577 in a figure with your ATP-gS and compound bound structures?

Main text line 88: it's unclear what is meant by high ATP and low SS-DNA. Is high ATP 10-fold above KM? Is low SS-DNA 10-fold below KM? Please clarify either in text or in methods.

Main text line 104-107 and extended data table 2: Isn't it likely that the lack of GI50 in some cell lines (or weaker GI50) is probably due to a slower doubling time for those particular lines? Probably the less active line only doubles once or twice in 4 days and so doesn't cross your 50% threshold. The more cell divisions that occur, the more likely the cells will show a quantitative GI50 response given the MOA for WRN inhibition. It might be interesting to look at the raw CTG values for your T0 and the untreated control and use that to calculate the number of cell doublings and see if there is any correlation between GI50 sensitivity and number of cell doublings. The simplest explanation for your CFA assay being more sensitive is because you have more cell doublings after 14 days.

Main text lines 40-41 & 168-170: I believe one of the significant implications for a WRN inhibitor and MSI patients is (as alluded in lines 40-41 of the intro) the potential for combination of WRN inhibitor with checkpoint inhibitors. Therefore, while the irinotecan data are interesting and have a clear mechanistic rationale, I'd strongly suggest also adding in the combination data with a checkpoint inhibitor, which may be more impactful from a clinical perspective. Can the authors provide any supporting data for checkpoint combo efficacy (for instance in a syngeneic MSI model system or whatever model system may be convenient)?

Main text line 177: "…and body weight was observed." This sentence seems incomplete.

Main text lines 152 and 177. The discussion around tolerability of WRN inhibition is very thin. Two sentences referencing body weight change is not sufficient for a Nature paper. I believe the authors should provide more information here. (A full toxicology study is required for IND package!) Can they

please include their data for broad kinase panel, CYP inhibition, herg, ion channel, etc. Is there any indication of any on target dose limiting tox? Did they determine a MTD for their compound and what was their efficacious dose relative to that MTD? Can they please make some estimate of their therapeutic index (understanding all the caveats in such an estimation)? Again, some of this discussion can be in supplementary section given the wordcount constraints.

Line 220: I think it's worth mentioning that the heparin HP column is to remove DNA that co-fractionates with WRN.

Lines 251-261 appears to be a copy paste error / duplication of lines 240-250.

Line 278: can you please mention something about the elution profile? Does it elute as a monomer / dimer …?

Line 297-316. Can you please mention the reported values you found for the KM with respect to ATP substrate and KM with respect to your DNA substrate? It's good to know from your HTS screen and assay conditions where your assay conditions are relative to KM.

Methods summary 201->end. I found these very clear, instructive, and well written.

Figure 4b and extended Figure 4e, can you please add a dotted line in your PK plots to indicate where you are modeling your anticipated GI90 coverage (corrected for protein plasma binding as appropriate)?

Referee #3 (Remarks to the Author):

Summary of Key Results: The authors describe new WRN-interacting compounds that inhibit WRN ATPase activity in vitro and show biological activity in cell-based assays and an in vivo xenograft model. Their findings lead credence to existing findings that WRN is synthetic lethal with microsatellite instable cancers.

Originality and Significance: While WRN helicase inhibitors have been described previously, this is the first report of their application to cancer cells with microsatellite instability, building upon findings by multiple groups that cancer cells with microsatellite instability rely on WRN for proliferation and DNA damage suppression.

Data & Methodology: For the most part Methods are described; however, I found lacking a clear description of the primary screen or hit optimization, as well as WRN depletion by RNA interference and some other more minor experimental details for the methodologies used.

Appropriate use of Statistics and Treatment of Uncertainties: Fine.

Conclusions: Robustness, Validity, Reliability: As outlined in my critical comments for authors, some issues (major and minor) are sub-optimal and overall take away some strength and robustness from

the conclusions being made.

Suggested Improvements: See Critical Comments where I have provided a detailed critique.

Clarity and Context: I found some sections of the manuscript to perfunctory in the language and interpretation of data. I found some errors in the figures. I found some deficiencies in the experiments performed-see Critical Comments for a detailed critique.

Critical Comments:
The authors write: "Several distinct chemical series have been published as WRN inhibitors, but no clinical drug candidate has been disclosed so far 26,27,28" They should mention at least 1 or 2 of the papers published in PNAS PMID: 21220316 , Canc Res PMID: 23867477 , Nat Commun PMID: 34772932 so that it can be appreciated that similar phenotypes (DNA damage induction, inhibition of cell proliferation, WRN protein degradation, etc), as well as additional ones (e.g., DNA replication) to those observed with the latest described inhibitor were previously reported for other characterized WRN helicase inhibitors.

The authors write: "Extensive hit finding activities resulted in a single validated hit 1 65 (ZINC21803075) whose optimization culminated in clinical candidate HRO761 (2, Fig. 1a)." It is unclear to me from inspection of Fig. 1a if a compound library was tested for small molecules that co-crystallized with WRN, or what precisely was the primary screen? Also in Fig. 1a, please define in the figure legend or the Methods what is meant by "hit optimization"? How exactly was this achieved?

The authors write: "Overlay of the D2 domains of ATPγS and HRO761 bound WRN shows that the hydroxy pyrimidine moiety of HRO761 mimics the γ-phosphate of ATP and recapitulates coordination of the hydrolytic water by the catalytic residue Gln850 (Fig. 1e)." I am confused and must be missing something here. Figure 1 in the files that were provided in the on-line submission only has a single panel letter: "b" I surmise that "a" is at the top left of the figure. However, there are no panel letters 1c through 1e.

The authors write: "To better reflect physiological conditions, we drove our potency optimization with a biochemical WRN ATPase assay with high ATP and low ssDNA concentrations. In this WRN ATPase assay, HRO761 has an IC50 of 100 nM. The inhibition of WRN helicase by HRO761 at the biochemical level translated into an antiproliferative effect in MSI colorectal SW48 cells, with a GI50 of 40 nM in a 4-day proliferation assay (Fig. 2a). Moreover, treatment with HRO761 showed that WRN helicase inhibition is sufficient to impair viability of MSI cancer cell models with GI50 values in the range of 50-1000 nM, while there was no effect in MSS cells in a 10-to-14-day clonogenic assay." The term "helicase" is written but in this context the term "helicase" is inappropriate as it might mislead a less informed reader that HR0761 was tested for inhibition of WRN helicase activity and I do not see experimental data in a figure that shows helicase data. Moreover, WRN has three catalytic activities: ATPase, helicase, and exonuclease. So, to write "helicase" here or elsewhere (e.g., Extended Data Figure 2) is misleading.

Were any biochemical WRN helicase assays performed with the compounds described in this study?

How did the IC50 values for ATPase inhibition compare with WRN helicase inhibition? Did the compounds adversely affect WRN exonuclease activity in biochemical assays?

It would be informative to have biochemically tested WRN (full-length) with the site-specific C727A and C727S site-specific mutations for their sensitivity to the small molecules described in this study as assayed by ATPase, helicase and exonuclease assays. If the compound is negatively affected for its physical interaction with WRN by the C727 mutations, then one would imagine the compounds would fail to inhibit WRN catalytic activities, following the authors' logic.

Either the figure or the figure legend for Fig. 2c is wrong. Fig. 2c shows y-axis PS50 and the x-axis GI50 [nM). However, the figure legend states: "c. Protein stabilization 50 (PS50) in the x-axis versus growth inhibition 50% (GI50) values in the y-axis, for HRO761 in a panel of MSI cells in red and in MSS cell in blue in a 4-day viability assay." Thus, the figure legend and figure itself do not match.

Figure 2c; The authors should use a different color other than pink for HCT116 cells with a C727A knock-in mutation at the WRN gene (PS50 = 675 nM), and RKO cells with a C727S knock-in mutation (PS50 = 8789 nM) because these too closely look like the red dots for the other data. Moreover, which pink dot corresponds to RKO cells with C727S knock-in mutation and HCT116 cells with C727A knock-in mutation? Why were different mutations tested (C727S versus C727A? Did the choice have something to do with soluble expression in the respective cell line or some other reason? They two displayed 10-fold difference in PS50 values—why is that?

The authors write: "Consistent with the selective viability effects of HRO761 in MSI cells, treatment with HRO761 led to activation of the DNA damage response (DDR) also selectively in cell lines sensitive to WRN inhibition, but not in cells that are insensitive to WRN inhibition or WRN knockdown." Unless I am missing it, I do not see any experimental data in the manuscript or description in the Methods pertaining to RNA interference for "WRN knockdown".

The authors write: "WRN has been described to be subject to post-translational modifications leading to ubiquitination and subsequent degradation after DNA damage and replication stress 30,31." References 30 and 31 describe the role of ubiquitin-proteasome and camptothecin treatment, respectively, in WRN stability. Several papers in the literature have provided compelling evidence that WRN acetylation plays a role in WRN stability/function, and one presents evidence that the effect can be modulated by a DNA damaging agent (mitomycin C). Have the authors considered WRN acetylation in the effects of their described compounds on WRN stability?

HR0761 was tested in vivo in SW48 cell-derived xenografts. SW48 cells were originally isolated from the large intestine of a colorectal cancer patient. Please provide a rationale for why this particular human cancer xenograft model was used. Could the human cancer cell xenograft model used contribute to differences from the other study mentioned in terms of dependence on p53 status?

It is very important to clearly convey throughout the manuscript for the biochemical assays that a WRN helicase core domain fragment, rather than full-length WRN, was used for tests with compounds. I recommend that throughout the manuscript where biochemical assays with WRN were performed that it be written "WRN(517-1238)" so as to no confuse the reader. Was full-length

WRN ever tested for inhibition of its catalytic activities by the small molecules described in this study?

I found it confusing that the WRN inhibitor(s) stabilize WRN but presumably as the authors contend DNA damage response elicited by the same compounds causes their destabilization. Did they test a cell line in which a specific DNA damage signaling molecule was deficient if the WRN inhibitor compound showed an attenuated ability to cause WRN destabilization in cells? Or can a DNA damage signaling pathways be pharmacologically inhibited to attenuate the WRN inhibitor compound-induced destabilization of WRN? What is the direct evidence that DNA damage signaling plays a role in the WRN inhibitor-induced WRN protein destabilization?

Figure 2e lacks a label to indicate what “-/+” refers to.

**Author Rebuttals to Initial Comments:**

**HRO761, an allosteric, first in class clinical WRN inhibitor, demonstrates synthetic lethality in MSI cancers**

Ferretti et al.

Referees' comments:

**Referee #1 (Remarks to the Author):**

**Major points**

If DNA damage initiates the degradation of WRN, are MSI cells far more sensitive to classic alkylating chemotherapy agents, or topoisomerase inhibitors, compared to MSS cells? If not, do these agents not lead to WRN degradation like they cite? A lot of their mechanism rests on this assumption being true.

➔ A manuscript published almost simultaneously with our submission ("Microsatellite instability states serve as predictive biomarkers for tumors chemotherapy sensitivity", Ye T., et al., iScience 26, 107045, July 21, 2023) analyzed the sensitivity of MSI vs MSS cells to chemotherapy agents, and they found that multiple MSI tumors were more sensitive to some of these agents compared to MSS cells, for example, etoposide and camptothecin. This is therefore consistent with our data showing that these agents also degrade WRN in MSS cells. The main difference is that HRO761 has no effect on MSS cells (including no WRN degradation) consistent with the synthetic lethality of WRN inhibition in MSI cells, and any of effect seen on MSS cells viability with the DNA damaging agents should not be due to WRN degradation since genetic screens have shown the synthetic lethality of WRN knockdown or knockout only in MSI with no effect in MSS cells.

**[REDACTED]**

Related to this, in cells treated with Compound 4 and ATMi, the authors show that WRN is no longer degraded (Extended Data Fig 3C). This suggests that DNA damage initiated by loss of WRN ultimately leads to the protein's degradation. Does cotreatment of cells with HRO761 and ATMi lead to better cell survival compared to treatment with HRO761 on its own? If not, then it seems that the Compound's effect is primarily via more short-term WRN inhibition and the degradation phenotype observed is then a secondary effect that is not driving cell death. The authors should be aware of a recent paper in which rapid WRN degradation does lead to synthetic lethality in MSI (https://www.biorxiv.org/content/10.1101/2023.07.28.550976v1).

➔ Reviewer #1 is correct and indeed cotreatment of cells with HRO761 and an ATMi leads to better cell survival compared to HRO761 on its own. Below please see additional data that we have incorporated in the manuscript in Extended Data Figures 3f and 3g

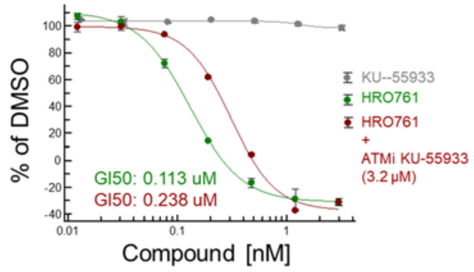


A shift in $GI_{50}$ is observed upon addition of ATM inhibitor KU-55933 in combination with HRO761 compared to HRO761 alone, consistent with an increased viability of the cells (4-day assay).

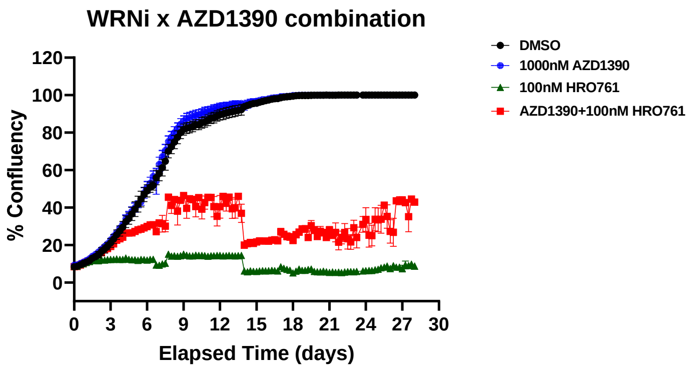


While treatment with HRO761 at 100 nM leads to an almost complete cell growth inhibition, addition of ATM inhibitor AZD1390 leads to better survival over time.

➔ We would like to apologize for the omission of the manuscript mentioned by the reviewer (https://www.biorxiv.org/content/10.1101/2023.07.28.550976v1) and point out that its publication on July 30th coincided with our submission. We have now cited it.

Fig 2b: Are there 8 MSS cell lines plotted, as indicated in the legend? It looks like 2 cell lines in the plot.

➔ We changed the plot to show all MSS cells which were overlapping.

Fig 2, panel c. The authors used the Protein Stabilization assay to show that binding of HRO761 to the target protein is comparable in MSI and MSS cells. This, together with genetic dependency uncovered by DRIVE, led the authors to conclude that HRO761 acts by selectively inhibiting WRN. Although certainly suggestive, to definitively show that this is the case the authors should **test** HRO761 in WRN-deficient cells complemented with exogenous WRN. Additionally, what are the units for PS50 on the y-axis?

➔ We have changed the y-axis to $PS_{50}$ [nM], apologies for the omission. We also show in Figure 2, panel c that the binding to WRN by HRO761 is decreased or abolished by the introduction of knock-in mutants where C727 of WRN has been replaced by either A (C727A) in HCT116, or to S (C727S) in RKO cells. The loss of binding correlates with loss of DDR pathway modulation as well as anti-proliferative effects as shown in a new panel we added to Extended Data Figure 3, panel b (below). This suggests that HRO761 is selectively inhibiting WRN in cells.

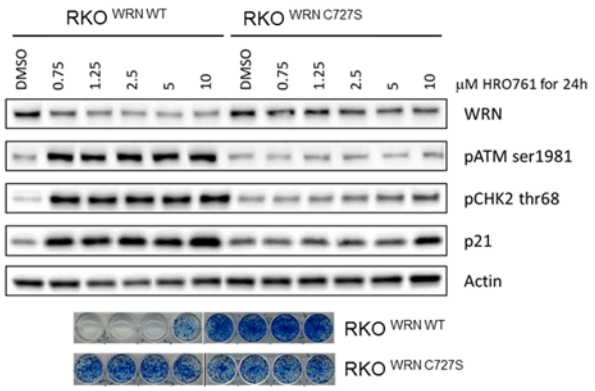


➔ Regarding the last point of the reviewer, as MSI cells do not tolerate WRN depletion, we would like to ask the reviewer to please specify how he would design this experiment.

Fig 2, panel e. Why do HRO761-treated MSI cells show diminished levels of WRN, when based on the protein stabilization assay HRO761 appeared to stabilize WRN? Also, in panel e it was shown that p21 was significantly induced in four MSI cell lines after HRO761 while in Extended Fig 1e, RKO cells depleted for WRN showed no induction of p21 (at least based on the zoomed out IHC image). At first glance, this appears to be internally inconsistent.

➔ We would like to clarify that the $PS_{50}$ assay is from protein extracts as described in the Methods section, and it measures stabilization of the WRN protein when bound to HRO761. Treatment of cells, on the other hand, leads to WRN degradation as shown in Figure 2, panel d (new version) and further confirmed at the protein by proteomics in Figure 2, panel f.

➔ In Extended Figure 1e, there is weak p21 induction as measured by IHC (score of +1), stronger at week1 than at week 2, but this is rescued with WRN WT re-introduction. We have included both time points for IHC for completeness as well as quantification for all 3 IHC markers in two new figure panels f and g (Extended Data Figure 1f and 1g, example for p21 below).

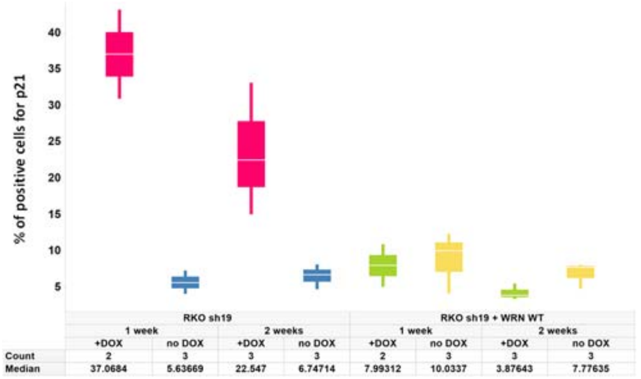


Fig 2, panel F (and in text). What method are they using to measure protein levels? How are they plotting three cell lines in one Volcano plot (explain if they are taking the average value for each protein). This experiment needs to be better explained in the figure legend. What is on the x-axis, what is on the y-axis?

➔ As described in the methods section, peptide/protein quantification was performed using tandem mass tags (TMT), an isobaric multiplexed chemical labeling method which is the de facto

standard for mass spec-based quantification. We have adapted the legend and clarified the axis labels.  We thank the reviewer for pointing out those omissions.

Fig 2, panel g. I assume that the data is presented as a heatmap to show that hypersensitivity to HRO761 in CFA correlates with reduced WRN protein level. However, the way the data is presented is not self-explanatory and the legend is somewhat confusing. What is Max/Average/Min and what does it refer to?

➔ We have changed the legend to clarify the data shown in this panel.

Fig 2, panel h and Extended Fig 4. What is the rationale for using analogs of HRO761 rather than HRO761 itself in these experiments? In panel h, there is no real point in testing DLD1 WRN-KO cells, since DLD1 is known to be refractory to WRN inhibition anyway.

➔ The RNASeq experiments were done with earlier analogs to ensure during the optimization process that the WRN inhibitors were indeed inhibiting WRN and having effects only in MSI and WRN sensitive cells. This is also the reason for including the DLD1 WRN-KO cells, to ensure that the WRN inhibitor had no off target effect unrelated to WRN inhibition since they do not have any WRN.

Extended Fig 3. Panel a, the figure legend states that cells were treated with camptothecin. However, the plot indicated that cells were treated with Eto, a commonly used abbreviation for etoposide. In panels b and c, it was shown that WRN degradation is mediated by ATM and the proteasome. It would be nice if the authors could test whether proteasome/ATM inhibition could partially rescue MSI cells from WRN inhibition. This may have important therapeutic implications since proteasome/ATM inhibitors are being developed/used as anti-cancer therapies.

➔ We apologize for the typo in this figure. We have changed the legend to etoposide (now Extended Data Figure 3c). Regarding proteasome rescue, we would like to point out that proteasome inhibition is very toxic to the cells and therefore this experiment was technically challenging as the cells were dying from the proteasome inhibitor alone. However, ATM inhibition did partially rescue the cells from HRO761 inhibition as mentioned in the first point.

Extended Fig 4. Panel a, it was stated that mice were treated once and sampled 4 h post last treatment. This statement is a bit unclear, does it mean mice were treated for 4 h and immediately sacrificed? That seems unlikely since many of the effects in cell lines took hours to days to manifest. What are the tumor cells used in panel a-c? In panel d, is 4% Reg short for 4% regression? At what dose does the WRN inhibitor become toxic?

➔ Mice were treated, and then sacrificed 4 hours post oral treatment. Tumors were from the SW48 cell line. We have modified the legend to clarify. In Extended Figure 7 panel c one can observe that the response to WRN inhibition is very quick even *in vivo*, with pATM and pChk2 modulation as early as 1 hour and p21 induction at maximum levels at 4 hours post oral treatment.
➔ We did not observe toxicity in mice at pharmacologically achievable doses, consistent with the clean profile of the compound and the synthetic lethality in MSI cells only.

Fig 3 and Extended Fig 5. The authors reported that HRO761 induces ATM activation within 1 h of treatment, while WRN degradation began after 8 h (extended fig 5). They also reported that the treatment induced G2 arrest by 24 h in Fig 3d, but earlier time points have not been investigated. Is it possible that G2 arrest caused/promoted WRN degradation? As it stands, although likely, it is not completely certain that HRO761 binding itself induces WRN degradation. Since treatment with CPT also was reported to degrade WRN (which should be confirmed), I wonder if WRN degradation simply reflects a general feature of WRN protein instability in G2, especially if the arrest is prolonged? The authors should test another treatment condition that induces G2 arrest in their cells and assess whether WRN degradation also occurs in that context. It is not sufficient to say that WRN degradation occurs only in WRN-sensitive cells because in the insensitive lines HRO761 treatment also does not lead to G2 arrest

- ➔ HRO761 binding itself does not lead to degradation, this is shown in Figure 2c and 2d where we show that HRO761 binds to cells like HT29, LS510 and CAL33 (we have modified the graph in Figure 2c to point out these lines), yet there is no WRN degradation (Figure 2d).
- ➔ We have tested nocodazole treatment after which cells arrest at the G2/M phase of the cell cycle and showed that there is no effect on WRN levels (please see below). Additionally, we have performed a cell cycle synchronization (with double thymidine block followed by release) and looked at WRN levels at different phases of the cell cycle and there is no modulation of WRN levels at the different phases of the cell cycle. Therefore, data suggests that WRN degradation is not due to the arrest of cells in G2. We have now added a panel to Extended Figure 3 (panel h) where we show this data:

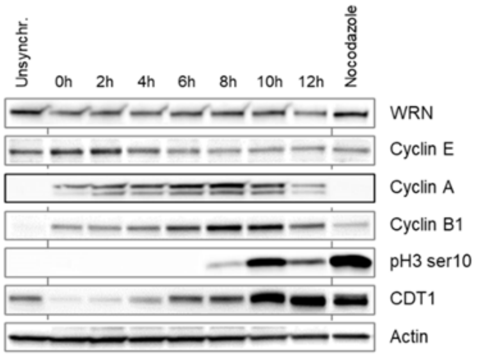


In Fig 3a, why was HRO761 labeled as RJT779; is that another name for the compound? In Extended Fig 5, it seems that in addition to WRN, treatment with HRO761 also induced time-dependent degradation of CHK1 (and CHK2). Thus, I'm wondering if (1) this property is also shared with CPT treatment, (2) what might be the cause of this phenomenon (hyper-instability of DDR factors in G2) and (3) can lethality really be attributed solely to WRN loss (being as CHK1 is an essential gene)?

- ➔ We have modified Figure 3a and apologize for the typo.
- ➔ CHK1 and CHK2 degradation occur at high HRO761 concentrations. At concentrations where there is no CHK1 and CHK2 degradation (i.e., 300 nM HRO761 and below) there is still growth inhibition. This argues against lethality at these concentrations being due to CHK1 or CHK2 loss. At the very high concentrations of HRO761 this possibility cannot be excluded.
- ➔ We have added a new Extended Data Figure 3i where we show that while etoposide leads to WRN degradation in SW48 and HCT116 cells there is no CHK1 or CHK2 degradation (below) and Camptothecin treatment in SW48 also leads to WRN degradation in SW48 cells with no CHK1 or CHK2 degradation. This might be cell line dependent as Camptothecin treatment in HCT116 cells leads to degradation of all 3 proteins, however of CHK1 and CHK2 at higher concentrations than WRN.

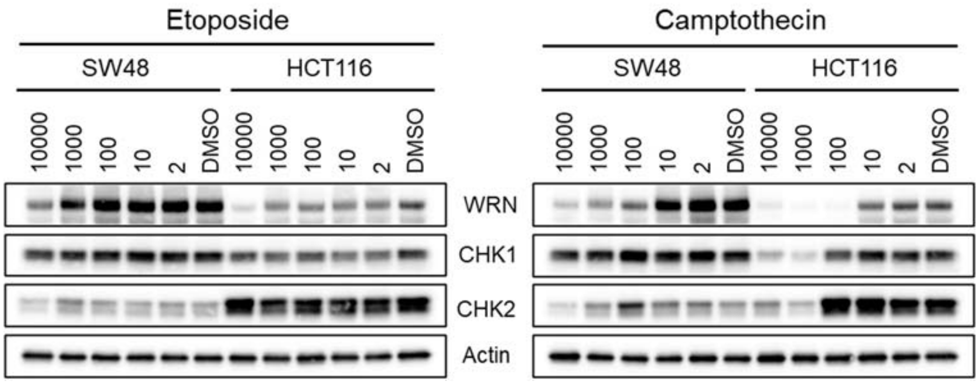


Fig. 4, panel a, treatment with HRO761 initially led to tumor regression, but tumor growth appeared to re-start towards the end of the experiment. Is this due to acquisition of resistance mechanisms?

- ➔ We have internal evidence to show that this is an on target, acquired mechanism of resistance and this data will be published in a later publication.

Fig 4C: Can the authors comment on why does KAP1 phosphorylation, a marker of DNA damage, decrease after Day 14 of HRO761 administration, even when pATM levels remain high and WRN levels remain low? The authors write that the DDR remains activated for 21 days, but this doesn't seem to be accurate and should be further discussed (lines 154-156).

- ➔ We have observed that as the tumors lose viability and start to regress, DDR activation starts to be shut down (e.g., pCHK2, pKAP1). It is true that only pATM remains activated by 21 days while the other markers decrease after 14 days. We have changed the text to accurately describe the findings.

Fig. 4, panel e, does the sensitivity of different MSI cell lines to HRO761 correlate with their genetic dependency on WRN? If so, it will be good to show it. If not, what might be the reason for such discrepancy? In CDXs that are less sensitive to HRO761, was WRN degradation induced at a lower level than in highly sensitive CDXs? Related to this issue, Extended Data Table 2 shows that the GI50 values of HRO761 in several MSI cell lines known to be highly susceptible to genetic loss of WRN (e.g., KM12, OVK18 and RKO) are much higher than even HCT116 C727A, in which WRN binding by HRO761 should be severely impaired. This is a bit surprising, given that other experiments showed equivalent binding of HRO761 to WRN independently of MS status or cell type. What might be the explanation for the relative lack of HRO761 activity in these MSI lines that are known to be WRN-dependent?

- ➔ Figure 2b shows the correlation between sensitivity of MSI cells to HRO761 and the correlation to their genetic dependency on WRN. The cell lines that the reviewer points out are sensitive to HRO761 inhibition in the longer CFA assay (RKO $GI_{50}$=243 nM, KM12 $GI_{50}$=100, OVK18 $GI_{50}$= 150 nM), as shown in the second column of Extended Data Table 2. In the graph depicting the *in vivo* efficacy experiments in Figure 4e, indeed the OVK18 and RKO appear insensitive. This is due to 2 reasons. Firstly, we are limited with the dosing in preclinical models, and we wanted to test all models at similar HRO761 doses of either 60 or 120 mg/kg. In these two models (OVK18 and RKO) we did not treat at doses higher than 120 mg/kg which are not efficacious. Secondly, more potent WRN inhibitors are efficacious in these models. We can share this data with the reviewer if necessary, but this data will be beyond the scope of the manuscript and confidential.

Fig. 4, panel f, the data seems to indicate that after drug washout, MSI cells recover from HRO761. Is HRO761-induced WRN inhibition reversible? This is something that needs to be carefully examined and will have significant impact on dosing.

- ➔ In Figure 4f there is no washout, cells treated with 100 nM HRO761 do become resistant while cells treated with 100 nM HRO761 and 40 nM irinotecan do not regrow except for a small subset of cells between day 30 and 35.

Extended Fig 7, panel b, are these SW48 cells? If so, why was there no reduction in WRN protein level observed at 24 h, while WRN was clearly degraded as shown in Fig 4c (60 mg/kg, day 1)? In general, when multiple cell line models are used in the same figure (e.g. SW48, HCT116 etc), it is good practice to label them accordingly (if not in the figures themselves then at least in the figure legends).

- ➔ We have clearly annotated the cell lines used for each figure. Apologies it if was not clear.
- ➔ We would like to clarify that in Figure 4c animals have been treated twice and tumors harvested 4 hours post second dose (this is indicated in the legend as 24+4 hours). In Extended Data figure 7c there was only one dose given to animals. This means that we need at least two doses to induce WRN degradation and the longer mice are treated the more degradation is observed.

Fig. 4 and Extended Fig 7. What is the proposed mechanism for the synergy observed between HRO761 and irinotecan? Based on Extended Fig 7g, it seems that perhaps DNA damage load is increased in cells dually treated with HRO761 and irinotecan. Is this because both HRO761 and irinotecan (inferred from CPT data) could induce partial WRN degradation, and when combined, simply induces more complete WRN degradation? In this regard, it would be interesting to test whether HRO761 synergizes with other drugs frequently used in treating colorectal cancers, such as platinum agents or 5-FU?

- ➔ Irinotecan is used as a standard of care in metastatic colorectal cancer, the indication where HRO761 will be used in the first in human (FiH) trial. We therefore wanted to test the potential combination of WRN inhibition with the SoC. In fact, combination of HRO761 with Irinotecan will be tested in one of the arms of the FIH clinical trial currently ongoing. The combination of Irinotecan + HRO761 has been validated in several CRC cell models. We have also tested 5-FU and platinum agents as combination partners for HRO761 treatment. These studies have shown some model -dependent combination benefit, but less striking than Irinotecan.
- ➔ As the reviewer well points, our data suggests that the mechanism underlying the combination with Irinotecan relies on the increased accumulation of DNA damage caused by the treatment with both compounds, as shown in Extended Data Figure 7g.
- ➔ We don't believe that increased WRN degradation induced by both compounds in MSI cells is the determinant for sensitivity, rather a consequence of the activation of a DNA damage response. WRN degradation is a described event downstream of DDR activation, independent of MSI status, while sensitivity to this combination is only observed in MSI.

Minor comments

Page 1, line 38, abbreviation (MMR) has already been introduced earlier (on line 35)

- ➔ We have corrected this, apologies and thank you for the suggestion.

Page 3, lines 87-88, the authors wrote "To better reflect physiological conditions, we drove our potency optimization with a biochemical WRN ATPase assay with high ATP and low ssDNA concentrations". Ideally, the authors should include some supplemental info on this assay showing that it indeed measures WRN activity, perhaps benchmarking against one of the previously published experimental WRN inhibitors. Or, if the data is not being published, they should indicate that in the text.

- ➔ We apologize for not spelling out clearly that our ATPase inhibitors also inhibit the helicase. In our hands, inhibition of the ATPase coincided with inhibition of DNA unwinding, i.e., the helicase activity. The revised manuscript mentions this, and we have described and added data for the unwinding assay in the Extended Data Figure 2c. We have also added information on the ATP $K_M$ in the ADPGlo assay method. This assay was the main one used for optimizing our series (the method is described). It was performed at 300 μM ATP corresponding to 20-fold $K_M$. In the

initial steps, as our chemical series displays a mixed ATP competitive mechanism, the same ATPase assay was performed at 15 μM corresponding to the ATP $K_M$. The hit discovery and optimization steps will be described in more detail in manuscripts in preparation.

Fig 1. Panel b, there is no explanation of the different colored domains. Is the inhibitor colored in red? Is panel c a zoom-in view of panel b, and if so, what region does it depict? Panels a, c, d, e are not clearly indicated.

➔ Figure 1 has been completely revised and the domains are labelled in the new figure.

Extended Fig 1. Panel a lacks a description of color coding. Panel c, the legend should include a description of the timing of the WB experiment. Panel d, there is no description of statistical significance; for shWRN + WRN K577A, the difference between the black and blue lines (if significant) are much smaller than that shown in the shWRN cohort. Does this indicate that the helicase-dead mutant may still retain some function in vivo? Information should ideally be provided in the legend or as part of the plot on the number of mice used for each condition.

➔ Suggested changes to Extended Data Figure 1 (panels a, c, d and now e) have been made. Regarding the shWRN + WRN K577A *in vivo*, we thank the reviewer for the careful observation regarding the significance and the possibility that the helicase-dead mutant may retain some function *in vivo*. Indeed, we observed that there was an increase in WRN levels between day 7 and day 14 in the WRN K577A mutant (now Extended Data Figure 1i, below)

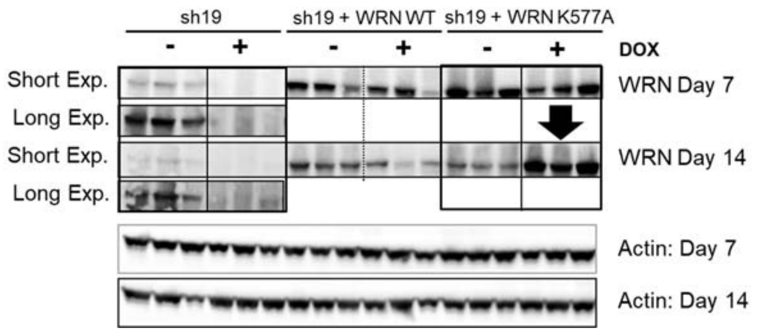


➔ Since the immunoblot could not differentiate between endogenous WRN and exogenous, K577A mutant WRN, we measured the transcript levels of both in tumors, and found that it was the K577A WRN mutant that was selectively upregulated in the tumors after 2 weeks of dox induction removing WRN WT (now Extended Data Figure 1j, below):

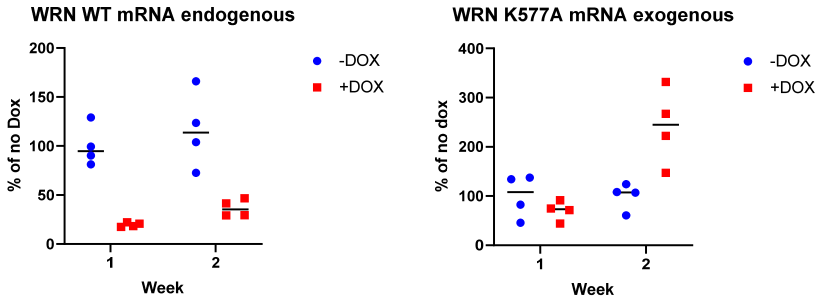


We therefore measured the enzymatic activity from both WT and K577A helicase domain proteins in an ATPase assay and found that the K577A WRN mutant is not completely dead but retains ~1-2 % of activity (now Extended Data Figure 1k, below). This explains why these tumors which upregulate the expression of the K577A WRN protein have enough helicase activity for their survival.

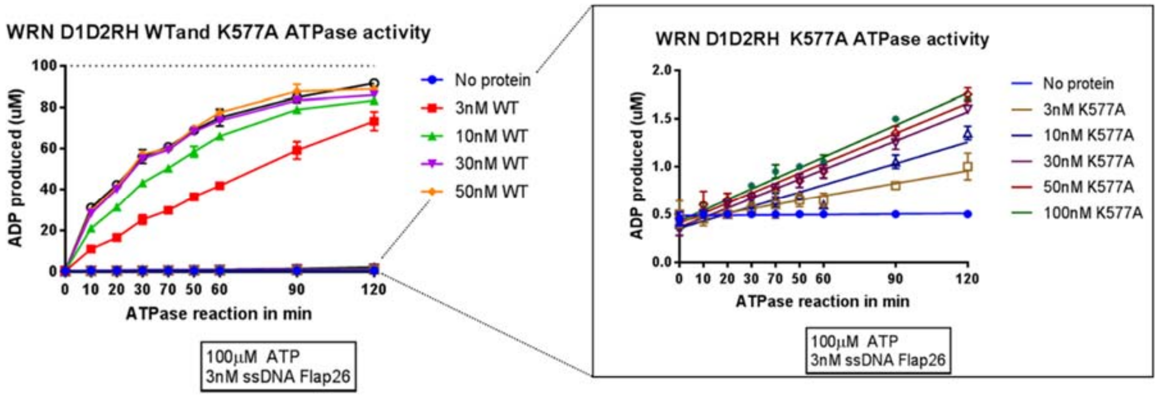


Extended Fig 1. Panel b shows clearly that re-introduction of WT or nuclease-dead WRN into WRN depleted cells rescues lethality. Surprisingly, in panel c, the levels of gH2AX did not appear to decrease in the rescued cells. Moreover, pATM, pChk2, pATR, pChk1 and p53 seemed to be comparable between WRN-depleted cells and cells that were reconstituted with WT WRN, while for cells complemented with E84A mutant, the levels of these DDR markers showed some decrease. These results are a bit confusing, how does exogenous WRN rescue lethality in WRN depleted cells without affecting DNA damage accumulation?

➔ We thank the reviewer for the careful examination of the data. As the reviewer might be aware, these genetic knockout and rescue experiments are technically challenging. The cells undergo DNA damage when WRN is knocked down, and the damage remains for some time even though cells with a WRN WT cDNA can recover and proliferate over time. But it is difficult to harvest enough cells from *in vitro* treatment. *In vivo* we observe the rescue of the DNA damage and proliferation in the shRNA WRN + WRN WT rescue, while there is only a partial rescue when the WRN K577A helicase dead mutant is introduced. We have added an additional panel to Extended Figure 1, panel h to show this (below):

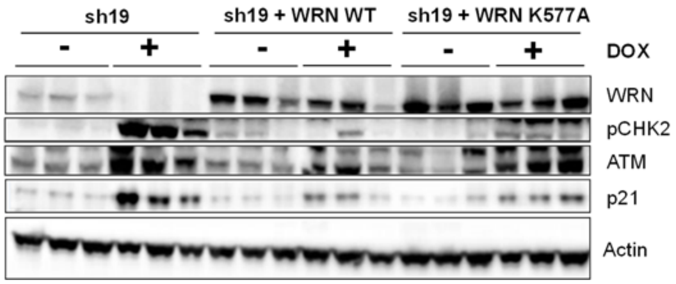


Extended Fig 2. Panels c and d, all of the y-axes have legends that look different in terms of presentation. Part of the legend is obscured in d, left panel.

➔ We thank the reviewer for pointing this out and have made the according changes.

Extended Fig 7. Panel d, it is not clear whether the “white line” at the bottom is the scale bar.

➔ We can make these changes, apologies if it was not clear.

Finally, it would be advisable to make the fonts bigger for most of the figures. As things stand right now, it’s almost impossible to read certain plots. An additional Extended Figure showing the full blots should also be appended at the end.

- ➔ We have modified the fonts and made our best effort to make the plots readable and we apologize for the quality of the images but we had to compress them for the initial submission due to size limitations.
- ➔ Regarding the full blots, we would like to ask for which blots the reviewer would like to see the full image, but also alert that we divide the membrane in several pieces to be able to blot for different markers so we do not have full blots for most of the experiments. We would like to ask the reviewer for the reason to ask for these blots.

**Referee #2 (Remarks to the Author):**

Abstract lines 20-21: "… of the first potent and selective WRN helicase inhibitor…": given that another potent and selective WRN inhibitor (H3B-968) has been published (Parker et al, Biochemistry 2023), please amend the phrase to "…first potent and selective clinical stage WRN helicase inhibitor…". H3B-968, while potent in biochemical assays, was limited from a properties point of view and not suitable for cellular based assays or in vivo studies. Therefore, keep the emphasis on "clinical stage", which is sufficiently remarkable and an exciting breakthrough for patients with MSI cancers. With this subtle wording change, you showcase your work without taking away from others.

- ➔ We agree with the reviewer and wording has been adapted to clinical stage as requested. Parker et al, Biochemistry is cited as ref. 28.

Clarifying the patient numbers affected by MSI and how many are unresponsive to checkpoint inhibitors may better represent the significance of the author's findings. For instance, main text line 42: "… a significant fraction of patients …": please check into the patient numbers to be more precise: "an estimated xx-yy number of patients…" and include appropriate citations.

- ➔ We have incorporated the suggested changes.

Main text line 64-65: "extensive hit finding activities…". It's not appropriate to title a paper "Discovery of HRO761…" and only have one sentence describing the discovery. Absent any of the underlying discovery data package, the title would need to change to "Characterization of HRO761…". The pharmaceutical industry is excited about WRN and other challenging helicase targets and the broader Nature audience will be eager to learn from your groundbreaking work and experience. Therefore, in keeping with your title, this section needs to be significantly elaborated. Given the wordcount constraints, this may be provided in the supplementary section. This section should contain a multi-panel figure with the following components:

- ➔ We have changed the title of the manuscript to "HRO761, an allosteric, first in class clinical WRN inhibitor, demonstrates synthetic lethality in MSI cancers" in line with the reviewer's comments. The hit finding and lead optimization represents a massive effort that will be described in several manuscripts currently in preparation. A first publication has just been submitted describing the challenges that we had to overcome in screening and showing that the initial WRN inhibitors from literature cannot be considered as specific WRN probes. This publication could be shared to the reviewer upon request.

(a) Screening funnel, including primary HTS assay, secondary assays/counter screens, number of compounds screened (and what libraries whether proprietary or otherwise) and number of hits passing your statistical thresholds at each stage, and please report the % activity of your hit in the primary assays. (How much did the primary hit stand out above the noise?)

→ Hit **1** was identified from several iterative screening campaigns. This multi-year effort consisted of 1) characterization of artefact propensity for efficient triaging of artefacts, 2) identification of a more sensitive assay format from the analysis of the MoA of covalent hits and screening compound libraries (in the

range of 1 million compounds), triaging and biophysical validation of **1**. A manuscript covering (1) has been submitted and manuscript (2) is currently in preparation.

(b) Key biophysical data of the primary screening hit. What were the key data that led you to identify this singleton and what gave you confidence that it was a real hit and not a non-specific binder/aggregator. *Please include a reasonable subset of supporting DSF / NMR / ITC / SPR / mass spec (HDX / ASMS / etc) data.*

→ Hit **1** was validated by DSF, SPR, $^{13}C$ HSQC (NMR) and a co-xtal structure of the close 3-Cl-4-methylaniline analogue (CID: 46269349) was obtained. Details will be revealed in manuscript 2 mentioned in the bullet above.

(c) What were the key biochemical data that support the MOA? Did the compound show ATP-competition / DNA competition or both (see comment below for lines 69-72)?

→ We do agree that the Lineweaver-Burk plots demonstrate a mixed competitive or non-competitive MOA for both ATP and DNA. We could demonstrate that HRO761 (and all derivatives in the series) are mixed ATP competitors and DNA noncompetitive (see further details below)

(d) What was your first crystal structure from the series that enabled structure-based drug design?

→ See comment (b) above.

(e) Please provide a table with 5-10 compounds with negative SAR points that validate the interactions observed in the crystal structure. E.g., changing an R-group that disrupts some H-bond interaction reduces the compound potency x-fold.

→ We feel that this is beyond the scope of this manuscript and this information will be published in the upcoming J. Med. Chem. manuscript.

(f) I would love to see some discussion around how you elaborated your different R-groups, but concede this may be more appropriate in a medchem focused follow up paper. Do we have any assurance from the authors that such a paper is underway? Are they able to share this draft?

→ As stated above, this will indeed be addressed in a J. Med. Chem. paper under preparation.

Lines 69-72 and extended figure 2. Two things I'd like to see included in your supplement. (1) From my understanding of your mechanistic data, the compound is competitive with respect to ATP and non-competitive with respect to DNA. Your Linweaver Burke plots tell a slightly different story of mixed inhibition for both. I'd suggest adding a table here reporting the IC50 value of the compound under conditions of [ATP] ~ KM and [ATP] ~100X KM. (high and low ATP). Under these conditions, we should see a clear shift in IC50 for an ATP competitive compound. Please also include data showing IC50 of compound with high and low DNA substrate concentrations. For a compound that is non-competitive with respect to DNA, we don't expect to see a shift in IC50. This may be a more intuitive way to present your MOA data given many readers may not readily recall the meaning of y-intercepts and slopes on a Linweaver Burke plot. (2) To further support your structural interpretation of MOA, I'd like to see additional data for 5 ITC experiments, which should be very easy to perform: First, WRN and titrate compound. Second, WRN and titrate DNA. Third, pre-form the WRN-DNA complex and titrate compound. Fourth, pre-form the WRN-compound complex and titrate DNA. Fifth, WRN and titrate ATP-gammaS (not sure if this last titration is technically feasible given the higher KD). These titration experiments will help prove if your compound binds in the presence of DNA and if DNA binds in the presence of compound. These are very easy experiments and will be highly interesting MOA data to support a non-competitive model. It will also be noteworthy to understand what the entropic cost is of rearranging your D1 and D2 domains upon compound binding.

- **Mixed ATP competitive mechanism:**

1) The reviewer is correct that $IC_{50}$ should shift with ATP concentration. However, as the $IC_{50}$ of HRO761 and close analogues is already at the sensitivity limit of the assay, the shift is less pronounced than anticipated (e.g., HRO761 at $K_M$ ATP: 18 nM *vs* at 20x $K_M$ ATP: 100 nM).
2) The initial hit to lead optimization was performed with the ATPase assay as described in the publication but using an ATP concentration at ATP = $K_M$ = 15 μM on WRN (D1D2RH) protein construct ($K_M$ in good agreement with literature data). With the improvement of potency over time, we increased the concentration of [ATP] to 300 μM (highest possible [ATP] in the ADPglo assay allowing us to keep a robust assay). This change in the protocol was driven by the ATP competition that we could see for all derivatives our lead series.
3) To further confirm some level of competition with ATP, we set up a HTRF assay with biotinylated-WRN (D1D2RH) and Cy5-ATPγS. All derivatives of HRO761 displayed strong inhibition properties in this assay (HRO761 itself displayed an $IC_{50}$ below the assay sensitivity limit) demonstrating the competitive MoA regarding ATP through an allosteric binding pocket as shown by X-Ray.
4)

| | **Cy5-ATPγS binding assay in TR-FRET - $IC_{50}$ μM** |
|---|---|
| **1** | 3.3 |
| **2** | 0.009 |
| **3** | 0.010 |
| **4** | 0.008 |
| **5** | 0.013 |

**Cy5-ATPgS binding assay using TR-FRET:** 9.8 µL avi-tagged WRN D1D2RH in 30 mM Tris-HCl pH7.5, 1 uM $MgCl_2$, 30 mM NaCl, 0.02% BSA, 0.1% Pluronic F127 were incubated with 200 nL compound for 45 min at room temperature in white 384-well plates (Greiner Bio-One, Cat. No. 781207). 10 µL Cy5-labelled ATPγS (EDA-ATPγS-Cy5, Jena Bioscience, Cat. No. NU-1609-CY5) and Europium-labelled streptavidin (LANCE Eu-W1024 Streptavidin, Perklin Elmer, Cat. No. AD0063) were added. Final concentrations in assay plate were 15 nM WRN, 10 nM ATPγS-Cy5, 2 nM Eu-Streptavidin. With the TECAN infinite M1000 PRO, Eu was excited at 340 nm and the fluorescence emission was measured at 620 nm (F620) and 665 nm (F665) after 30 min. A ratio of F665 over F620 was then calculated to determine the FRET efficiency in each well.

5) With this knowledge and the Lineweaver-Burk plots performed with the ADPglo assay, we can conclude that the MoA for HRO761 is mixed ATP competitive.
6)

➢ **DNA noncompetitive mechanism:**

1. Despite extensive efforts, we were not able to identify suitable ITC conditions, due to the poor stability of the WRN protein constructs (aggregates detected in the ITC device leading to highly variable results). We appreciate the ITC protocol described in Parker et al, Biochemistry 2023 performed on the full length WRN protein which we could only produce in small quantities that did not allow ITC experiments.
2. After several assays developed to answer this key question (WRN/DNA binding assay using FP, ATPase assay at low/high DNA, Cy5-compound probe in TR-FRET with/without DNA), the best results were obtained with a radioactive compound probe binding assay ("[$^3$H]-BWQ" / HRO761 derivative). Saturation experiments showed nice specific binding properties to WRN (D1D2RH) protein construct, and the same results were obtained in presence of saturating concentrations of ssDNA ("FLAP26" – see below) or dsDNA.

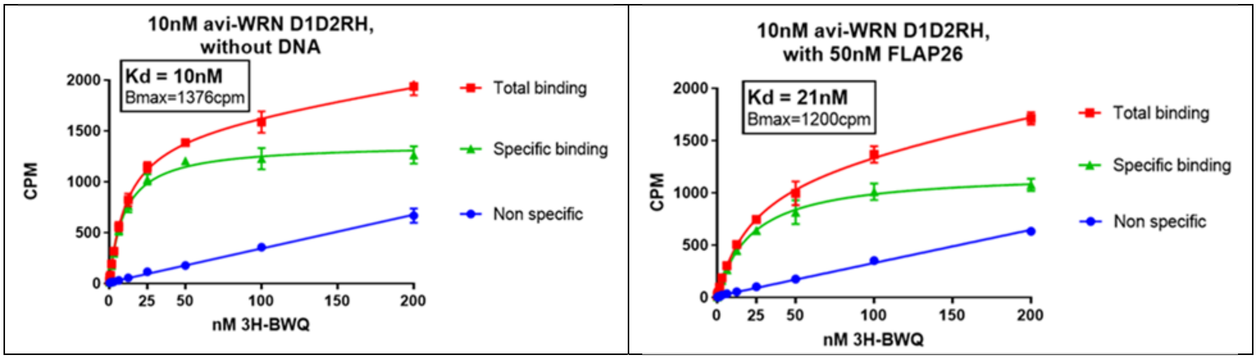


All these assay results will be included in the next publication under preparation.

Figure 1: The focus of the paper is HR0761. Please include in Figure 1 a summary table with the key target compound profile (TCP) data including MW/cLogD/PSA/solubility/biochemical and cellular activity/selectivity against WRN vs BLM, selectivity in one representative MSI vs one representative MSS cell line, mouse and human liver microsome, mouse and rat or dog or monkey PK including CL, Vss, AUC, and %F. These are the most critical summary data which should be highlighted in the first publication of a clinical stage compound.

➔ We have added a summary table with key data of HRO761 as Extended Data Table 1.

Figure 1B: Please indicate in your figure, using a different color, what are the hinge residues and please explain the color scheme in the legend. Is the hinge supposed to be the yellow residues and what are the three mysterious yellow balls?

➔ Fig 1b has been changed accordingly.

Figure 1C: This should be one of the most exciting figures in the paper and I would be more than happy to assist the authors if they send me the coordinate file! Otherwise, these are my requests for improvement. (1) With the current color scheme, the compound is camouflaged and difficult to see. The compound is the most important part of the figure, so this should be in a complimentary or brighter color relative to the protein while the protein should be in a more neutral color (not bright blue). (2) The chosen view is unclear. One should never showcase a view where an alpha helix is completely blocking the object you're trying to observe (the compound). I would request the authors to pivot the view ~90 degrees so this helix is more behind/above the compound with the core sitting above Cys727 (this view will also make the rationale for your beautiful target engagement knock-in data for C727A/S at lines 98-99 much more clear). From this view, it will be also easier to highlight how the compound acts like a molecular zipper employing a fascinating H-bond network with several Arg residues from D1/D2 to stabilize this novel conformation! This new view will place the obstructing helix in their current view behind or above their compound and not in front of their compound. Please also cite Parker et al 2023, which proposed the existence of an allosteric pocket around C727, although they were unable to elucidate the impressive conformational changes identified here. Finally, please reorient the 2D-representation of Figure 1A to match the pose in the new view. (3) the authors make some objects semi-transparent and use depth cueing. This makes it hard to tell if something is in front or in back. Use depth cue but avoid semi-transparent rendering. (4) If some residues are unavoidably obstructing a clear view of the compound, crop them out (while minimizing cropping as much as possible) and make a note of this in the legend. (5) far too many interactions are shown, which makes the figure incredibly busy and hard to decipher. Please show only the most critical interactions mentioned in the text including the key Arg-interactions with the compound forming the molecular zipper. (6) None of the residues is labeled. Please label the key Arg residues (711, 854, 857) and Cys 727 in a reasonable and unobtrusive font and color scheme so as not to distract from the compound.

- ➔ We thank the reviewer for his offer of assistance. Fig. 1 has been simplified to focus on key interactions and further details will be shown in the J. Med. Chem. paper. We feel that the citation of the hypothesis of an allosteric pocket from Parker et al 2023 requested by the reviewer is more appropriate in our hit finding paper which will include a co-crystal structure of a FBS and covalent hit binding Cys727.

Figure 1 D-E, these can be moved to supplementary to make room for requested TCP table above.

- ➔ We feel that the new Figure 1d is more appropriately placed in the main manuscript to support the MoA discussion. As request, we have included a TCP table as Extended Data Table 1.

Figure 2A, please label the Y-axis as % growth. This plot seems pixelated. Please make a better figure and not a low-resolution snapshot. I would highly recommend displaying two curves in this figure, one representing MSI cell line and one for MSS. This will nicely illustrate the selectivity of your compound and the window. Please remove the red-dashed lines that show the GI50.

- ➔ We have made the suggested changes.

Figure 3F, similar comments to 2A. Please provide meaningful label on Y-axis and make higher quality. The lines are too thin, aesthetically needs improvement. Recommend printing all figures and inspecting their visual quality.

- ➔ We have made the suggested changes.

Main text line 79: please show SPR summary table with data illustrating the on/off rates of your lead and final compound. Please include the sensograms and the data fit to a 1:1 kinetic model and heterogeneous ligand model. How do these data fit and is there any circumstantial evidence for two conformational states for WRN?

- ➔ Despite extensive efforts, we were not able to identify robust SPR assay conditions. We used SPR assays on WRN D1D2RH or WRN D1D2 protein constructs as part of the hit validation steps. SPR was useful for yes/no answer before getting better validation of compound binding by 2D-NMR. However, SPR assay variability was too high to consider using these assays for SAR or kinetic parameter assessment. Kinetic assessment was better investigated by a kinetic probe competition assay (kPCA)

Extended Data figure 1b: You're showing the exonuclease domain as a hexamer. I recommend showing it as a monomer to match your schematic. Otherwise, please explain the hexamer in the legend because this won't be obvious to anyone except the specialists who've read John Tainer's paper.

- ➔ We thank the reviewer for the suggestion and have adapted the figure accordingly.

Extended Data figure 1b-d): for the K577A mutant, please mention this is the walker motif and critical for g-phosphate interactions. Would also be nice to relate your genetic functional data to the compound pharmacologic data if possible. In other words, can you tell from the crystal structure does the compound push K577 out of position? Can you show comparison of K577 in a figure with your ATP-gS and compound bound structures?

- ➔ As suggested by the reviewer, Figure 1d shows the effect of compound binding on the Walker motif. The displacement of the Walker motif from the D1D2 interface to a solvent exposed region explains the low residual activity of ATP to HRO761-bound WRN.

Main text line 88: it's unclear what is meant by high ATP and low SS-DNA. Is high ATP 10-fold above KM? Is low SS-DNA 10-fold below KM? Please clarify either in text or in methods.

➔ Concentrations are stated in the methods, and we have included $K_M$s in the revised manuscript. For WRN D1D2RH, ATP $K_M$ was 15 µM and the "High [ATP]" ATPase assay condition was at [ATP]=300 µM = 20-fold $K_M$. The text has been updated.

Main text line 104-107 and Extended Data table 2: Isn't it likely that the lack of GI50 in some cell lines (or weaker GI50) is probably due to a slower doubling time for those particular lines? Probably the less active line only doubles once or twice in 4 days and so doesn't cross your 50% threshold. The more cell divisions that occur, the more likely the cells will show a quantitative GI50 response given the MOA for WRN inhibition. It might be interesting to look at the raw CTG values for your T0 and the untreated control and use that to calculate the number of cell doublings and see if there is any correlation between GI50 sensitivity and number of cell doublings. The simplest explanation for your CFA assay being more sensitive is because you have more cell doublings after 14 days.

➔ We agree with the reviewer that the CFA assay's higher sensitivity is due to more cell doublings and therefore DNA damage accumulation. However, we have not seen a correlation between $GI_{50}$ sensitivity and number of cell doublings. We have included an additional column in Extended Data table 2 with the doubling times of the cell lines.

Main text lines 40-41 & 168-170: I believe one of the significant implications for a WRN inhibitor and MSI patients is (as alluded in lines 40-41 of the intro) the potential for combination of WRN inhibitor with checkpoint inhibitors. Therefore, while the irinotecan data are interesting and have a clear mechanistic rationale, I'd strongly suggest also adding in the combination data with a checkpoint inhibitor, which may be more impactful from a clinical perspective. Can the authors provide any supporting data for checkpoint combo efficacy (for instance in a syngeneic MSI model system or whatever model system may be convenient)?

➔ While we acknowledge the relevance of the ICI combination, and this combination is part of our first in human study design, we do not have any data to support this due to the absence of an appropriate syngeneic MSI model.

Main text line 177: "…and body weight was observed." This sentence seems incomplete.

➔ We have amended this sentence. Apologies for the omission.

Main text lines 152 and 177. The discussion around tolerability of WRN inhibition is very thin. Two sentences referencing body weight change is not sufficient for a Nature paper. I believe the authors should provide more information here. (A full toxicology study is required for IND package!) Can they please include their data for broad kinase panel, CYP inhibition, herg, ion channel, etc. Is there any indication of any on target dose limiting tox? Did they determine a MTD for their compound and what was their efficacious dose relative to that MTD? Can they please make some estimate of their therapeutic index (understanding all the caveats in such an estimation)? Again, some of this discussion can be in supplementary section given the wordcount constraints.

➔ The off-target profile is summarized in Extended Data Table 1. We understand the reviewer's keen interest in tox data. At this point, we can only reveal that the high exposure margins and preclinical safety packaged were sufficient to support the FDA and EU approval and the clinical study start.

Line 220: I think it's worth mentioning that the heparin HP column is to remove DNA that co-fractionates with WRN.

➔ The HP column was first used as a standard purification step, not only to remove DNA but we added this comment.

Lines 251-261 appears to be a copy paste error / duplication of lines 240-250.

➔ We will correct this sentence. Apologies for the omission.

Line 278: can you please mention something about the elution profile? Does it elute as a monomer / dimer …?

➔ By size exclusion chromatography we see only one peak for WRN core helicase domain (D1D2) and WRN D1D2RH and experiments using Mass Photometry could clearly determine the expected MW for a monomer.

Line 297-316. Can you please mention the reported values you found for the KM with respect to ATP substrate and KM with respect to your DNA substrate? It's good to know from your HTS screen and assay conditions where your assay conditions are relative to KM.

➔ We added the information that our main ATPase assay was performed at 20-fold ATP $K_M$ and at concentration of DNA = $K_{DNA}$. We used another set of screening assays in the initial steps of hit finding which will be described in a manuscript in preparation as mentioned above.

Methods summary 201->end. I found these very clear, instructive, and well written.

Thank you.

Figure 4b and extended Figure 4e, can you please add a dotted line in your PK plots to indicate where you are modeling your anticipated GI90 coverage (corrected for protein plasma binding as appropriate)?

➔ We have added the $GI_{90,unbound}$ to the PK data on Figure 4b and have additionally included another panel to figure 4b that shows the correlation between efficacy and unbound blood area under the curve ($AUC_{b,u}$) above SW48 unbound $GI_{90}$.

**Referee #3 (Remarks to the Author):**

Data & Methodology: For the most part Methods are described; however, I found lacking a clear description of the primary screen or hit optimization, as well as WRN depletion by RNA interference and some other more minor experimental details for the methodologies used.

➔ The hit finding and lead optimization represents a massive effort that will be described in several manuscripts currently in preparation for submission in J. Med. Chem. The RNA interference data as well as methods are well described in the manuscript.

The authors write: "Several distinct chemical series have been published as WRN inhibitors, but no clinical drug candidate has been disclosed so far 26,27,28" They should mention at least 1 or 2 of the papers published in PNAS PMID: 21220316 , Canc Res PMID: 23867477 , Nat Commun PMID: 34772932 so that it can be appreciated that similar phenotypes (DNA damage induction, inhibition of cell proliferation, WRN protein degradation, etc), as well as additional ones (e.g., DNA replication) to those observed with the latest described inhibitor were previously reported for other characterized WRN helicase inhibitors.

➔ We have included two of the references requested by the reviewer. For reasons that will be detailed in a forthcoming manuscript, we were unable to conclude on differences in cellular phenotypes.

The authors write: "Extensive hit finding activities resulted in a single validated hit 1 65 (ZINC21803075) whose optimization culminated in clinical candidate HRO761 (2, Fig. 1a)." It is unclear to me from inspection of Fig. 1a if a compound library was tested for small molecules that co-crystallized with WRN, or what precisely was the primary screen? Also in Fig. 1a, please define in the figure legend or the Methods what is meant by "hit optimization"? How exactly was this achieved?

- ➔ Hit **1** was identified from several iterative screening campaigns spanning a multi-year effort consisting of 1) characterization of artefact propensity for efficient triaging of artefacts, 2) identification of a more sensitive assay format from the analysis of the MoA of covalent hits and screening compound libraries (in the range of 1 million compounds), triaging and biophysical validation of 1. A manuscript covering 1) has been submitted and manuscript 2) is currently in preparation. The medchem story for the discovery of HRO761 is also in preparation.

The authors write: "Overlay of the D2 domains of ATPγS and HRO761 bound WRN shows that the hydroxy pyrimidine moiety of HRO761 mimics the γ-phosphate of ATP and recapitulates coordination of the hydrolytic water by the catalytic residue Gln850 (Fig. 1e)." I am confused and must be missing something here. Figure 1 in the files that were provided in the on-line submission only has a single panel letter: "b" I surmise that "a" is at the top left of the figure. However, there are no panel letters 1c through 1e.

- ➔ We apologize for the omission and have added the panel letters to the improved Figure 1.

The authors write: "To better reflect physiological conditions, we drove our potency optimization with a biochemical WRN ATPase assay with high ATP and low ssDNA concentrations. In this WRN ATPase assay, HRO761 has an IC50 of 100 nM. The inhibition of WRN helicase by HRO761 at the biochemical level translated into an antiproliferative effect in MSI colorectal SW48 cells, with a GI50 of 40 nM in a 4-day proliferation assay (Fig. 2a). Moreover, treatment with HRO761 showed that WRN helicase inhibition is sufficient to impair viability of MSI cancer cell models with GI50 values in the range of 50-1000 nM, while there was no effect in MSS cells in a 10-to-14-day clonogenic assay." The term "helicase" is written but in this context the term "helicase" is inappropriate as it might mislead a less informed reader that HR0761 was tested for inhibition of WRN helicase activity and I do not see experimental data in a figure that shows helicase data. Moreover, WRN has three catalytic activities: ATPase, helicase, and exonuclease. So, to write "helicase" here or elsewhere (e.g., Extended Data Figure 2) is misleading.

Were any biochemical WRN helicase assays performed with the compounds described in this study? How did the IC50 values for ATPase inhibition compare with WRN helicase inhibition? Did the compounds adversely affect WRN exonuclease activity in biochemical assays?

- ➔ We apologize for not spelling out clearly that our ATPase inhibitors also inhibit the helicase. In our hands, inhibition of the ATPase coincided with inhibition of DNA unwinding, i.e., the helicase activity. The revised manuscript mentions this, and we have described the unwinding assay in the Methods and added data in the Extended Data Figure 2.

It would be informative to have biochemically tested WRN (full-length) with the site-specific C727A and C727S site-specific mutations for their sensitivity to the small molecules described in this study as assayed by ATPase, helicase and exonuclease assays. If the compound is negatively affected for its physical interaction with WRN by the C727 mutations, then one would imagine the compounds would fail to inhibit WRN catalytic activities, following the authors' logic.

It is very important to clearly convey throughout the manuscript for the biochemical assays that a WRN helicase core domain fragment, rather than full-length WRN, was used for tests with compounds. I recommend that throughout the manuscript where biochemical assays with WRN were performed that it be written "WRN(517-1238)" so as to no confuse the reader. Was full-length WRN ever tested for inhibition of its catalytic activities by the small molecules described in this study?

- ➔ In our hands, there were no significant differences between full length WRN and smaller constructs (i.e., even the minimal D1D2 construct is fully active). We thank the reviewer for pointing this out and we have clarified the use of constructs in the revised manuscript. We have included full length and C727S/A biochemical data in the Extended Data Figure 2.

Either the figure or the figure legend for Fig. 2c is wrong. Fig. 2c shows y-axis PS50 and the x-axis GI50 [nM). However, the figure legend states: “c. Protein stabilization 50 (PS50) in the x-axis versus growth inhibition 50% (GI50) values in the y-axis, for HRO761 in a panel of MSI cells in red and in MSS cell in blue in a 4-day viability assay.” Thus, the figure legend and figure itself do not match.

- ➔ We apologize for the inconsistency and have made the suggested changes.

Figure 2c; The authors should use a different color other than pink for HCT116 cells with a C727A knock-in mutation at the WRN gene (PS50 = 675 nM), and RKO cells with a C727S knock-in mutation (PS50 = 8789 nM) because these too closely look like the red dots for the other data. Moreover, which pink dot corresponds to RKO cells with C727S knock-in mutation and HCT116 cells with C727A knock-in mutation? Why were different mutations tested (C727S versus C727A? Did the choice have something to do with soluble expression in the respective cell line or some other reason? They two displayed 10-fold difference in PS50 values—why is that?

- ➔ We have incorporated the suggested changes and added details on the mutants to the legend and added biochemical data to explain the difference in potency in the different C727 mutants in Extended Data Figure 2

The authors write: “Consistent with the selective viability effects of HRO761 in MSI cells, treatment with HRO761 led to activation of the DNA damage response (DDR) also selectively in cell lines sensitive to WRN inhibition, but not in cells that are insensitive to WRN inhibition or WRN knockdown.” Unless I am missing it, I do not see any experimental data in the manuscript or description in the Methods pertaining to RNA interference for “WRN knockdown”.

- ➔ The data mentioned with shRNA knockdown of WRN is shown in Extended Figure 1. Methods are described under the sub-section “Generation of inducible shRNA constructs, viral production, and infection”.

The authors write: “WRN has been described to be subject to post-translational modifications leading to ubiquitination and subsequent degradation after DNA damage and replication stress 30,31.” References 30 and 31 describe the role of ubiquitin-proteasome and camptothecin treatment, respectively, in WRN stability. Several papers in the literature have provided compelling evidence that WRN acetylation plays a role in WRN stability/function, and one presents evidence that the effect can be modulated by a DNA damaging agent (mitomycin C). Have the authors considered WRN acetylation in the effects of their described compounds on WRN stability?

- ➔ We have tested acetylation and found that HRO761 treatment did not lead to acetylation of WRN as tested with an anti-acetylated lysine antibody (CST catalog #9441) with or without immunoprecipitation of WRN.

HR0761 was tested in vivo in SW48 cell-derived xenografts. SW48 cells were originally isolated from the large intestine of a colorectal cancer patient. Please provide a rationale for why this particular human cancer xenograft model was used. Could the human cancer cell xenograft model used contribute to differences from the other study mentioned in terms of dependence on p53 status?

- ➔ Please note that SW48 is only one of the models tested, chosen because of its sensitivity to shRNA knockdown of WRN. Figure 4e shows efficacy of HRO761 is a wider panel of xenograft models, both cell line derived and patient derived.

I found it confusing that the WRN inhibitor(s) stabilize WRN but presumably as the authors contend DNA damage response elicited by the same compounds causes their destabilization. Did they test a cell line in which a specific DNA damage signaling molecule was deficient if the WRN inhibitor compound showed an attenuated ability to cause WRN destabilization in cells? Or can a DNA damage signaling pathways be pharmacologically inhibited to attenuate the WRN inhibitor compound-induced destabilization of WRN?

What is the direct evidence that DNA damage signaling plays a role in the WRN inhibitor-induced WRN protein destabilization?

- ➔ The protein stabilization assay is an *in vitro* assay using cell extracts which is used as a surrogate target engagement assay to measure binding of HRO761, as compared with the WRN degradation elicited in cells.

Figure 2e lacks a label to indicate what “-/+” refers to.

- ➔ We apologize for the omission and have added the labels.

**Reviewer Reports on the First Revision:**

Referees' comments:

Referee #1 (Remarks to the Author):

The referee's comments have been adequately addressed, and manuscript has improved, but there are still issues in the text and figures that should be addressed:

The second paragraph in the Main Text section is a run on, or is not easy to understand. At the end of the paragraph the authors site 9 panels at once (Extended Data Fig. 1b-k). The authors should describe their data in more detail. It's not clear which panel in the figure corresponds to which statement in this paragraph.

Figure 4d: Scale bars are missing

Extended Figures 4d-ef are never mentioned in the text. What do these experiments/panels address?

Extended Figure 6a and 6b should be cited separately, and not just as Extended Figure 6.

Extended Figure 7d: needs scale bars. The image resolution is very poor, to the point that it is impossible to understand what is being displayed in this panel.

Are there new data points in Fig. 2C? It looks substantially different from the previous version.

Extended Fig 3b, please indicate the doses of HRO761 used in the CFA.

The legend for Fig 2g is now better explained. However, the color legend is still a bit confusing. I would suggest the authors to change the words "Max" to "high" and "Min" to "low".

In Figure 4e: Why do p53 null HCT116 xenografts have a better response to the drug compared to p53 WT cells?

Referee #2 (Remarks to the Author):

Please see attached PDF - a couple of my comments include graphics that aren't captured here.

In general, I found the authors' changes and rebuttal to be acceptable and I extend my compliments regarding Figure 1 which is vastly improved. My enthusiasm remains that this is a tour-de-force drug-discovery paper nicely integrated with biological MOA in the context of well validated / meaningful / impactful cancer biology.
Major Concern

• I am somewhat disappointed by the authors' insistence to withhold half the drug-discovery storyline (hit-to-lead) and would like to see some additional elaboration of this narrative as previously requested by both referee's 2 & 3. This can be accommodated with minimal effort and without undermining their follow-up publications. (Adding a third corresponding author may help alleviate internal friction over authorships if this is an issue.)
• The authors wish to publish the MOA of a first in class inhibitor in Nature. For this preeminent journal and audience, the data should be shown. Please expand extended Figure 2 with additional panels and include the nice data provided in the rebuttal letter. This is critically important data to support their claims.

Minor concern
• There are several instances where the authors claim in their rebuttal to have made changes to the text but I am unable to find any changes in the revision. They don't provide any track changes to the document. This makes it difficult to confirm if any changes were made. For example:

The wording in the abstract remains unchanged.
Therefore, please carefully examine that the manuscript text conforms to the rebuttal. Provide track changes as necessary.

Referee #3 (Remarks to the Author):

My previous comments on this manuscript addressed points in categories A-H. Here I will add comments pertaining to the revised version of the manuscript.

Feretti et al., HRO761, an allosteric, first in class clinical WRN inhibitor, demonstrates synthetic lethality in MSI cancers

The authors have revised their manuscript addressing some of the deficiencies raised in my original review. They have made figure and text corrections, included the WRN unwinding assay data, included full-length WRN and C727S/A biochemical data, and clarified some points that I had missed in my original review.
However, I found responses to several of my critical comments to be vague and not very instructive. It seems like the authors would want to explain to the readership certain concepts so that they would 1) understand what are the unique aspects of the current work compared to what has been done with WRN inhibitors and 2) comprehend and possibly utilize certain experimental design and approaches. I found that certain concepts still lack definitive explanation in the revised manuscript as it is currently written. See below.

Author Response to my comment that the description of the primary screen or hit optimization is unclear: "The hit finding and lead optimization represents a massive effort that will be described in several manuscripts currently in preparation for submission in J. Med. Chem." This may be the case, however it does not address the point. In my mind, a reader of this manuscript if it was published would have great difficulty in understanding how the primary screen was designed and executed,

and what guiding points were utilized for lead optimization. The response “massive effort that will be described in several manuscripts currently in preparation…” is simply not helpful to this reviewer, and frustrate the readership who are trying to understand the approaches.

Author Response to my comment that the authors place in context other WRN inhibitor papers describing similar phenotypes (acute DNA damage induction, inhibition of cell proliferation, WRN protein degradation, etc), as well as additional ones (e.g., DNA replication) to those observed with the latest inhibitor was vague: “For reasons that will be detailed in a forthcoming manuscript, we were unable to conclude on differences in cellular phenotypes.” The authors are encouraged to discuss concisely the similarities and differences of their findings with those that were previously published, using other small molecular WRN helicase inhibitors. This would allow the readers to appreciate the novelty of this work, as well as prevailing themes for pharmacological inhibition of WRN helicase activity.

Author Response to my inquiry about the details of the primary screen was obscure: “Hit 1 was identified from several iterative screening campaigns spanning a multi-year effort consisting of 1) characterization of artefact propensity for efficient triaging of artefacts, 2) identification of a more sensitive assay format from the analysis of the MoA of covalent hits and screening compound libraries (in the range of 1 million compounds), triaging and biophysical validation of 1. A manuscript covering 1) has been submitted and manuscript 2) is currently in preparation.” The problem with this response is that those who would read this paper would not be able to reproduce the strategy you used for the primary screening or be able to validate the experimental approach because details are lacking. In my mind, this is a significant issue because an important component of publishing a scientific research article is to inform the readership what was done so that it can be reproduced and built upon.

**Author Rebuttals to First Revision:**

Reply to referees' comments, letter from November 17, 2023:

Referee #1 (Remarks to the Author):

The referee's comments have been adequately addressed, and manuscript has improved, but there are still issues in the text and figures that should be addressed:

The second paragraph in the Main Text section is a run on, or is not easy to understand. At the end of the paragraph the authors site 9 panels at once (Extended Data Fig. 1b-k). The authors should describe their data in more detail. It's not clear which panel in the figure corresponds to which statement in this paragraph.

- ➔ We have modified the text to include more detail and included a section in the Supplementary Information (SI) file with a full description of Extended Figure 1 which cannot be included in the main text due to space constraints.

Figure 4d: Scale bars are missing

- ➔ Resolution of the scale bars is suboptimal in the pdf file due to the need to compress images, but they are present and they should be visible in the high resolution file of the figure.

Extended Figures 4d-ef are never mentioned in the text. What do these experiments/panels address?

- ➔ In the revised version where we have included compound optimization data, we have relocated these panels to be Extended Figure 7 and we have mentioned the data in the text. The data demonstrate that although compound **3** (formerly **4**) had the cellular potency similar to HRO761, it had suboptimal physchem properties (as described in the new panel b of Figure 1) and it needed twice daily dosing of 150 mg/kg to achieve tumor stasis, while HRO761 achieves tumor stasis at once daily dosing of 20 mg/kg.

Extended Figure 6a and 6b should be cited separately, and not just as Extended Figure 6.

- ➔ We have added a separate mention to each of the panels.

Extended Figure 7d: needs scale bars. The image resolution is very poor, to the point that it is impossible to understand what is being displayed in this panel.

- ➔ As for Figure 4d, resolution of the scale bars is suboptimal in the pdf file file due to the need to compress images, but they are present, and they should be visible in the high resolution file of the figure.

Are there new data points in Fig. 2C? It looks substantially different from the previous version.

- ➔ Figure 2c was only modified to change the colors of the C727 knock-in mutant lines to highlight these lines more clearly. Figure 2b does have additional data that was generated, and we also staggered the less sensitive lines to not overlap at the 10 µM HRO761 mark. We will provide the raw data in excel format with the final manuscript.

Extended Fig 3b, please indicate the doses of HRO761 used in the CFA.

- ➔ We have now indicated the doses of HRO761 used under the picture of the CFA.

The legend for Fig 2g is now better explained. However, the color legend is still a bit confusing. I would suggest the authors to change the words “Max” to “high” and “Min” to “low”.

➔ We have made the suggested change in legend nomenclature to “high” and “low”.

In Figure 4e: Why do p53 null HCT116 xenografts have a better response to the drug compared to p53 WT cells?

➔ We have analyzed DRIVE data and we do not see any statistically significant differences in responses to WRN genetic knockdown between p53 WT and p53 mutant or null cells as shown in the graph below, where p53 WT cells are in blue and and mutant or null are in red.

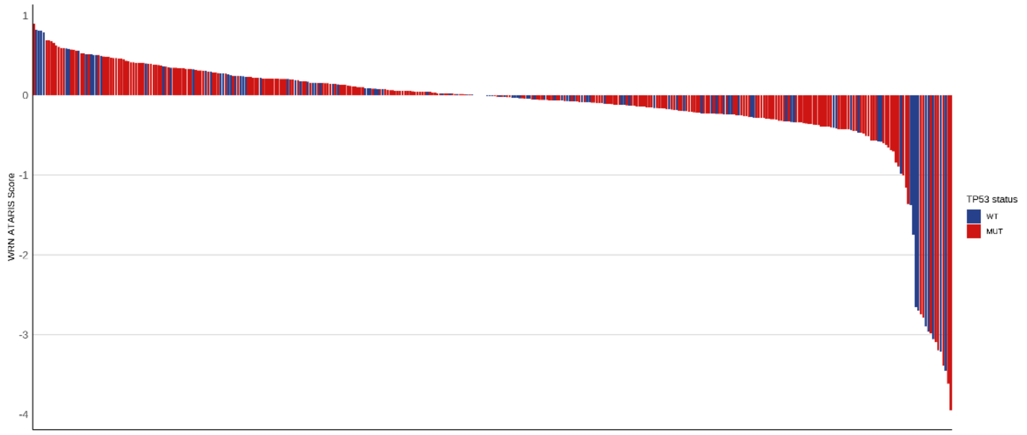


As also shown in Figure 3f, we do not see any difference to HRO761 in viability between the HCT116 WT and p53 null cells *in vitro*. Our explanation for the slightly better response to HRO761 in the HCT116 p53 null tumors *in vivo* is that it could be due to clonal differences. Unfortunately, we did not test additional clones since these were the only ones we had available.

Referee #2 (Remarks to the Author):

Please see attached PDF - a couple of my comments include graphics that aren't captured here.

In general, I found the authors’ changes and rebuttal to be acceptable and I extend my compliments regarding Figure 1 which is vastly improved. My enthusiasm remains that this is a tour-de-force drug-discovery paper nicely integrated with biological MOA in the context of well validated / meaningful / impactful cancer biology.

Major Concern

• I am somewhat disappointed by the authors’ insistence to withhold half the drug-discovery storyline (hit-to-lead) and would like to see some additional elaboration of this narrative as previously requested by both referee’s 2 & 3. This can be accommodated with minimal effort and without undermining their follow-up publications. (Adding a third corresponding author may help alleviate internal friction over authorships if this is an issue.)

➔ We thank the reviewer for her/his enthusiasm and perseverance in describing the drug discovery in more detail. We have expanded the drug-discovery storyline with a schematic representation as well as a description of the hit finding and LO campaigns. Our reluctance to do so in the previous version stems from the level of simplification required due to space limitations but we have tried to supply with the information necessary for understanding of the HF and LO campaigns as well reproducibility for the readers.

• The authors wish to publish the MOA of a first in class inhibitor in Nature. For this preeminent journal and audience, the data should be shown. Please expand extended Figure 2 with additional panels and include the nice data provided in the rebuttal letter. This is critically important data to support their claims.

➔ We have now included the methods and the requested data in the extended Figure 2.

Minor concern

• There are several instances where the authors claim in their rebuttal to have made changes to the text but I am unable to find any changes in the revision. They don't provide any track changes to the document. This makes it difficult to confirm if any changes were made. For example:

The wording in the abstract remains unchanged.
Therefore, please carefully examine that the manuscript text conforms to the rebuttal. Provide track changes as necessary.

➔ We apologize for not having used track changes in the revision. We have carefully checked all rebuttal answers in the text. Given the substantial changes made to the current version, we have not kept the track changes but would like to point to the reviewer a completely new paragraph #3 in the main text with the hit finding and lead optimization strategy. We have also modified the abstract to reflect the additional data added to the manuscript and the title following the reviewer suggestions.

Referee #3 (Remarks to the Author):

My previous comments on this manuscript addressed points in categories A-H. Here I will add comments pertaining to the revised version of the manuscript.

Feretti et al., HRO761, an allosteric, first in class clinical WRN inhibitor, demonstrates synthetic lethality in MSI cancers

The authors have revised their manuscript addressing some of the deficiencies raised in my original review. They have made figure and text corrections, included the WRN unwinding assay data, included full-length WRN and C727S/A biochemical data, and clarified some points that I had missed in my original review.
However, I found responses to several of my critical comments to be vague and not very instructive. It seems like the authors would want to explain to the readership certain concepts so that they would 1) understand what are the unique aspects of the current work compared to what has been done with WRN inhibitors and 2) comprehend and possibly utilize certain experimental design and approaches. I found that certain concepts still lack definitive explanation in the revised manuscript as it is currently written. See below.

Author Response to my comment that the description of the primary screen or hit optimization is unclear: "The hit finding and lead optimization represents a massive effort that will be described in several manuscripts currently in preparation for submission in J. Med. Chem." This may be the case, however it does not address the point. In my mind, a reader of this manuscript if it was published would have great difficulty in understanding how the primary screen was designed and executed, and what guiding points were utilized for lead optimization. The response "massive effort that will be described in several manuscripts currently in preparation…" is simply not helpful to this reviewer, and frustrate the readership who are trying to understand the approaches.

➔ As requested by reviewer 2 and this reviewer, we have added a description of the hit finding and LO campaigns, as well as binding assays used in the hit finding

campaign and additional biochemical data (please see new Figure 1 as well as Extended Data Figure 2).

Author Response to my comment that the authors place in context other WRN inhibitor papers describing similar phenotypes (acute DNA damage induction, inhibition of cell proliferation, WRN protein degradation, etc), as well as additional ones (e.g., DNA replication) to those observed with the latest inhibitor was vague: “For reasons that will be detailed in a forthcoming manuscript, we were unable to conclude on differences in cellular phenotypes.” The authors are encouraged to discuss concisely the similarities and differences of their findings with those that were previously published, using other small molecular WRN helicase inhibitors. This would allow the readers to appreciate the novelty of this work, as well as prevailing themes for pharmacological inhibition of WRN helicase activity.

➔ Given the focus on the discovery and characterization of HRO761 and space constraints, we feel that an in-depth comparison is outside the scope of this paper. We have cited the papers mentioned by reviewer 3 and referenced their covalent mode of action in contrast to the noncovalent MoA of HRO761.

Author Response to my inquiry about the details of the primary screen was obscure: “Hit 1 was identified from several iterative screening campaigns spanning a multi-year effort consisting of 1) characterization of artefact propensity for efficient triaging of artefacts, 2) identification of a more sensitive assay format from the analysis of the MoA of covalent hits and screening compound libraries (in the range of 1 million compounds), triaging and biophysical validation of 1. A manuscript covering 1) has been submitted and manuscript 2) is currently in preparation.” The problem with this response is that those who would read this paper would not be able to reproduce the strategy you used for the primary screening or be able to validate the experimental approach because details are lacking. In my mind, this is a significant issue because an important component of publishing a scientific research article is to inform the readership what was done so that it can be reproduced and built upon.

➔ We have added a description of the hit finding strategy including the methods for the ATP-binding assay not originally included and which was critical for triaging out false positives and identifying the noncovalent hit **1**.

**Reviewer Reports on the Second Revision:**

Referees' comments:

Referee #1 (Remarks to the Author):

The authors have adequately addressed most comments from the reviewers.

Referee #2 (Remarks to the Author):

I find this third revision much improved with the new paragraph on hit finding / optimization and data supporting the MOA. It's interesting that the ATP-competition binding assay identified the non-covalent starting hit. The added competition data is also nice evidence for the proposed MOA with respect to ATP and DNA. I'm supportive for publication. Congratulations again on the beautiful work.

-Nick Larsen

Referee #3 (Remarks to the Author):

The authors have addressed most of the critical points that I raised. The manuscript is much improved in terms of the new information provided and clarity of the communicated information.
